# Lung epithelial and endothelial damage, loss of tissue repair, inhibition of fibrinolysis, and cellular senescence in fatal COVID-19

**DOI:** 10.1126/scitranslmed.abj7790

**Published:** 2021-11-17

**Authors:** Felice D’Agnillo, Kathie-Anne Walters, Yongli Xiao, Zong-Mei Sheng, Kelsey Scherler, Jaekeun Park, Sebastian Gygli, Luz Angela Rosas, Kaitlyn Sadtler, Heather Kalish, Charles A. Blatti, Ruoqing Zhu, Lisa Gatzke, Colleen Bushell, Matthew J. Memoli, Steven J. O’Day, Trevan D. Fischer, Terese C. Hammond, Raymond C. Lee, J. Christian Cash, Matthew E. Powers, Grant E. O’Keefe, Kelly J. Butnor, Amy V. Rapkiewicz, William D. Travis, Scott P. Layne, John C. Kash, Jeffery K. Taubenberger

**Affiliations:** 1 Laboratory of Biochemistry and Vascular Biology, Center for Biologics Evaluation and Research, Food and Drug Administration, Silver Spring, MD, USA.; 2 Institute for Systems Biology, Seattle, WA, USA.; 3 Viral Pathogenesis and Evolution Section, Laboratory of Infectious Diseases, National Institute of Allergy and Infectious Diseases, National Institutes of Health, Bethesda, MD, USA.; 4 Section on Immunoengineering, National Institute of Biomedical Imaging and Bioengineering, National Institutes of Health, Bethesda, MD, USA.; 5 Bioengineering and Physical Sciences Shared Resource, National Institute of Biomedical Imaging and Bioengineering, National Institutes of Health, Bethesda, MD, USA.; 6 National Center for Supercomputing Applications, University of Illinois at Urbana-Champaign, Urbana, IL, USA.; 7 Department of Statistics, University of Illinois at Urbana-Champaign, Urbana, IL, USA.; 8 Clinical Studies Unit, Laboratory of Infectious Diseases, Division of Intramural Research, National Institute of Allergy and Infectious Diseases, National Institutes of Health, Bethesda, MD, USA.; 9 Saint John’s Cancer Institute, Santa Monica, CA, USA.; 10 Division of Cardiothoracic Surgery, USC Keck School of Medicine, Los Angeles, CA, USA.; 11 Department of Surgery, University of Washington, Harborview Medical Center, Seattle, WA, USA.; 12 Department of Pathology and Laboratory Medicine, University of Vermont Medical Center, Burlington, VT, USA.; 13 Department of Pathology, New York University Long Island School of Medicine, Mineola, NY, USA.; 14 Department of Pathology, Memorial Sloan Kettering Cancer Center, New York, NY, USA.

## Abstract

Coronavirus disease 2019 (COVID-19), caused by the severe acute respiratory syndrome coronavirus 2 (SARS-CoV-2), is characterized by respiratory distress, multiorgan dysfunction, and, in some cases, death. The pathological mechanisms underlying COVID-19 respiratory distress and the interplay with aggravating risk factors have not been fully defined. Lung autopsy samples from 18 patients with fatal COVID-19, with symptom onset-to-death times ranging from 3 to 47 days, and antemortem plasma samples from 6 of these cases were evaluated using deep sequencing of SARS-CoV-2 RNA, multiplex plasma protein measurements, and pulmonary gene expression and imaging analyses. Prominent histopathological features in this case series included progressive diffuse alveolar damage with excessive thrombosis and late-onset pulmonary tissue and vascular remodeling. Acute damage at the alveolar-capillary barrier was characterized by the loss of surfactant protein expression with injury to alveolar epithelial cells, endothelial cells, respiratory epithelial basal cells, and defective tissue repair processes. Other key findings included impaired clot fibrinolysis with increased concentrations of plasma and lung plasminogen activator inhibitor-1 and modulation of cellular senescence markers, including p21 and sirtuin-1, in both lung epithelial and endothelial cells. Together, these findings further define the molecular pathological features underlying the pulmonary response to SARS-CoV-2 infection and provide important insights into signaling pathways that may be amenable to therapeutic intervention.

## INTRODUCTION

More than 221 million confirmed severe acute respiratory syndrome coronavirus 2 (SARS-CoV-2) infections with more than 4.2 million coronavirus disease 2019 (COVID-19) deaths have been recorded worldwide as of September 2021. Many individuals suffer asymptomatic or mild-to-moderate symptoms, whereas others develop severe disease characterized by major pulmonary involvement, respiratory distress, systemic thrombosis, and death (1–4). Viral infection is mediated mainly by the binding of SARS-CoV-2 to the angiotensin–converting enzyme 2 (ACE2) receptor expressed on the surface of lung epithelial cells, endothelium, pericytes, and other cell types (1–4). The major reported lung pathologies of severe COVID-19 include diffuse alveolar damage (DAD), organizing pneumonia, and chronic interstitial pneumonia (1–3, 5). Pulmonary and systemic inflammation with vascular-related complications, including pulmonary embolisms, abnormal microthrombi, strokes, and direct blood vessel damage, highlight the critical pathogenic involvement of endothelial dysfunction in severe COVID-19 (6, 7). Key risk factors associated with severe COVID-19 include advanced age (>80 years), diabetes, obesity, male gender, and hypertension (8–12).

Alveolar-capillary barrier dysfunction, which is characterized by impaired gas exchange, vascular leakage, and reduced fluid clearance, is a major cause of the acute respiratory distress syndrome (ARDS) in patients with severe COVID-19. The alveolar-capillary barrier consists of (i) the alveolar epithelium composed of simple squamous alveolar type 1 (AT1) cells and surfactant-producing cuboidal AT2 cells, (ii) the alveolar capillary endothelium, and (iii) the collagen- and laminin-rich alveolar basement membrane separated by a sparse connective tissue interstitium. Permeability across the alveolar epithelium and endothelium is tightly regulated by intercellular junctional complexes, including cadherin-expressing adherens junctions and claudin-rich tight junctions (13–16). Loss or destabilization of epithelial or endothelial junctions is often a key feature of ARDS, acute lung injury, and microbial infections (13–16). Direct endothelial damage or infection also disrupts vascular tone regulation as well as the anti-inflammatory and antithrombogenic properties of the endothelium. Damaged or denuded alveolar epithelium triggers repair processes that are often defective or over-whelmed during infection (16, 17).

Research efforts have advanced the understanding of the pathophysiology of SARS-CoV-2 infection. However, key questions remain regarding the factors associated with severe and fatal disease outcomes. Moreover, delineating the mechanisms underlying the increased disease severity in individuals with advanced age, hypertension, diabetes, and other risk factors remains paramount (8–11). In the present study, lung autopsy tissue samples from 18 patients with fatal COVID-19 and antemortem plasma samples from 6 of these cases were examined using viral genomics, host genetic susceptibility single-nucleotide polymorphism (SNP) analysis, serum antibody and multiplexed cytokine and chemokine protein measurements, and pulmonary transcriptomic and imaging analyses to better understand the molecular pathological processes underlying COVID-19 respiratory failure.

## RESULTS

Postmortem lung autopsy samples (*n* = 18) and antemortem plasma samples (from six of the cases) were collected from a case series of confirmed SARS-CoV-2–positive patients who died between March and July 2020. The mean age was 67.7 years (range, 39 to 101 years; ten males and eight females). Symptom onset-to-death (SOTD) time intervals ranged from as early as 3 days up to 47 days (Table 1). All cases had respiratory failure, and mechanical ventilatory support was provided for 12 cases (Table 1). All patients presented with at least one known comorbidity (table S1). In-hospital clinical chemistry data available for a subset of these patients revealed elevated plasma C–reactive protein, ferritin, and D-dimers (table S1). Anti–SARS-CoV-2 antibodies were detected in the six cases with available antemortem plasma samples, and neutralizing antibodies were found in five of these cases (table S2). RNA sequencing (RNA-seq) analysis revealed loci of several previously reported risk alleles for severe COVID-19 (18–23). The *IFITM3* promoter (cases 9 and 13), *APOe4* (cases 8, 10, 11, and 13), dipeptidyl peptidase 9 (cases 9, 11, and 13), *OAS3* (cases 1 to 3), and *IFNAR2* (case 9), but not *IFITM3* (rs12252), were identified in 8 of 13 cases analyzed by RNA-seq (table S3). RNA-seq analysis also identified evidence of possible secondary bacterial infection with *Moraxella* (7 of 13 cases), *Pseudomonas* (6 of 13 cases), *Acinetobacter* (5 of 13 cases), and *Streptococcus* species (2 of 13) in a subset of 13 patient lung samples analyzed by next-generation sequencing (table S4). Next-generation sequencing of normal lung samples showed a typical lung microbiota (table S4).

### SARS-CoV-2 viral titration by reverse transcription polymerase chain reaction and viral genome SNP analysis by next-generation sequencing

RNA extracted from formalin-fixed, paraffin-embedded (FFPE) autopsy lung tissues were positive by quantitative reverse transcription polymerase chain reaction (qRT-PCR) for SARS-CoV-2 in all 18 cases (Table 1). A total of 20 sequencing libraries were constructed from RNA isolated from the first 13 cases, including multiple libraries from different FFPE lung blocks of several cases (table S5). SARS-CoV-2 viral reads were mapped to SARS-CoV-2/human/USA/NY-PV08472/2020 as the reference genome. All samples passing Nextclade quality thresholds belonged to either Nextclade lineage 20B or 20C (table S6 and fig. S1). Typing the strains with Pangolin software revealed that all viruses belonged to different B lineages (fig. S1), which had been isolated in Europe and the United States contemporaneously from the cases in this study. One sample belonged to the B.1.172 lineage, which has only been isolated in the United States. A total of 198 unique SNP positions were identified in the SARS-CoV-2 genome from these 13 SARS-CoV-2–infected FFPE lung samples, and the number of synonymous and non-synonymous SNPs in each case that passed the defined filtering criteria (see Materials and Methods) is shown in tables S7 to S9 as compared to the reference genome. Nonsynonymous SNPs were detected in 11 cases (tables S7 to S9), and 16 SNPs were shared by more than one case (table S9). The SARS-CoV-2 Spike (S) protein D614G mutation has been linked to higher infectivity (24). In all cases evaluated, the SARS-CoV-2 strains encoded this mutation (table S10). When compared to the amino acid replacements in SARS-CoV-2 genomes tracked by CoV-GLUE, among 136 unique non synonymous SNPs identified in our study, 56 sites have been recorded by CoV-GLUE, and 80 have not been reported by CoV-GLUE (tables S7 to S9). Noticeably, 19 unique nonsynonymous SNPs are in the S protein region, and 12 have not been reported by CoV-GLUE. Among these 19 S protein nonsynonymous SNPs, there are 2 SNPs within the receptor-binding region (RBD), including S371P and A419V. The S protein S371P SNP is within the epitope of neutralizing antibody H014, primarily located in the α2-β2-η2 (residues 368 to 386), η3 (residues 405 to 408 and 411 to 413), α4 (residue 439), and η4 (residues 503) regions (25). The S protein A419V SNP is close to the epitope of neutralizing antibody CC12.1, including K417 (also the ACE2-binding site) and K420 (fig. S2) (26). A number of SARS-CoV-2 variants have been reported (27), including the variants of concern Alpha, Beta, Gamma, and Delta. None of the key nonsynonymous mutations defining these variants were observed in the 13 cases examined by next-generation sequencing. The functional significance of the SNPs in other SARS-CoV-2 open reading frames (ORFs), including ORF1a and ORF1b, will require further study.

### DAD and impaired lung tissue repair after SARS-CoV-2 infection

**Fig. 1.**
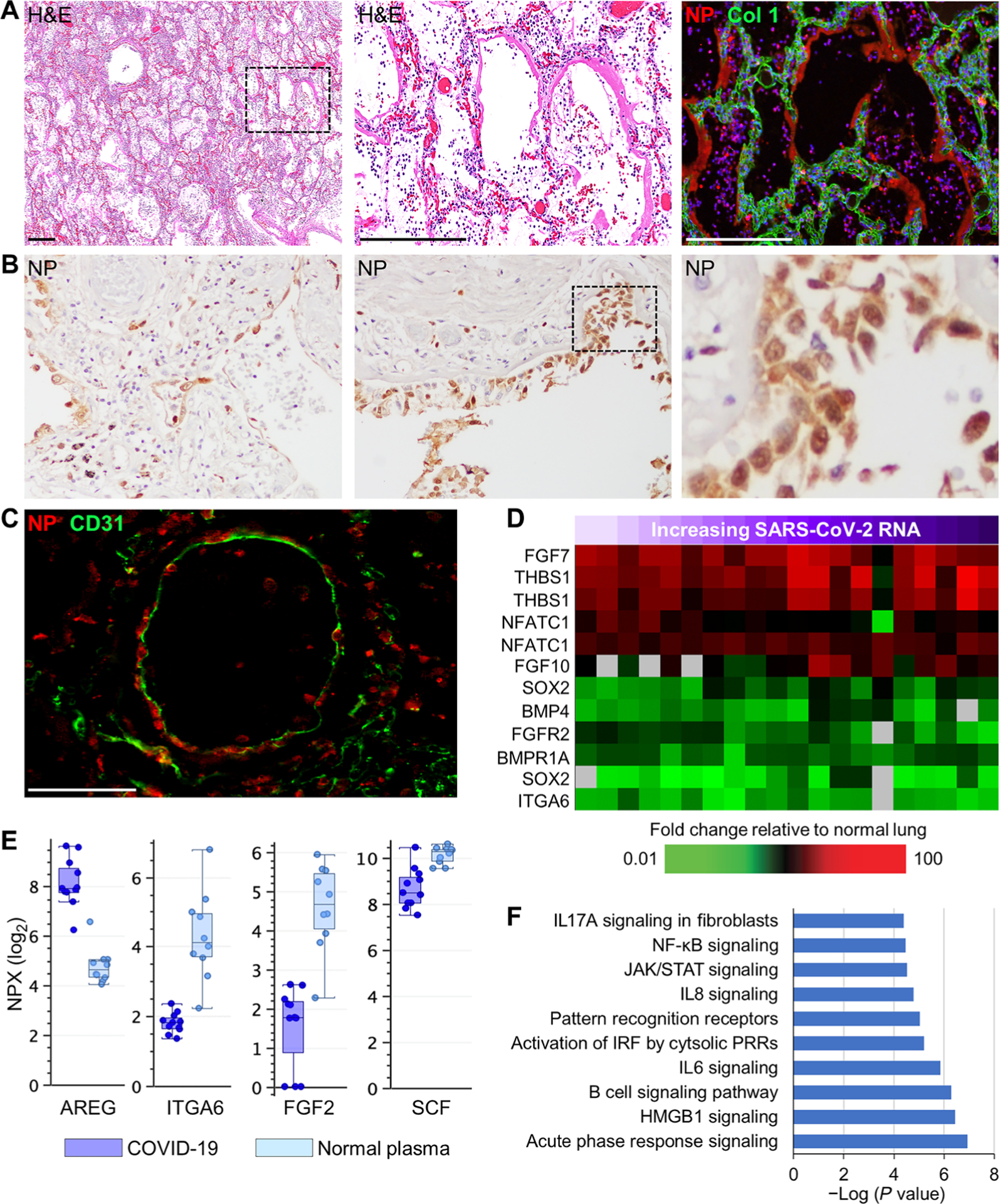
SARS-CoV-2 detection and host gene expression in COVID-19 lung autopsy samples. (**A**) Shown are representative images from serial lung autopsy sections from a patient with COVID-19 with acute DAD (case 1). Sections were stained with H&E or immunostained for the SARS-CoV-2 nucleocapsid antigen (NP) and collagen type 1 (Col 1). The digitally magnified boxed area shows marked NP immunoreactivity in hyaline membranes lining alveoli and intra-alveolar epithelial debris and cellular infiltrates. Nuclei were counterstained with Hoechst 33342 dye (blue). (**B**) Representative images of NP immunohistochemistry show positive staining in bronchiolar epithelium, epithelial basal cells, and alveolar epithelium (case 2). Black dashed box shows area of digital enlargement. Original magnification, ×200. (**C**) Immunofluorescence image of a medium-sized blood vessel stained for NP and endothelial CD31 (case 7). (**D**) Heatmap shows expression of genes encoding lung repair–related proteins in lung tissue from 13 COVID-19 cases (table S5). Each column represents gene expression data from a microarray experiment comparing RNA from COVID-19 lung tissue to pooled RNA isolated from normal lung tissue (*n* = 3). Genes shown in red were significantly increased (twofold, *P* < 0.05, using standard *t* test with Benjamini-Hochberg correction), genes shown in green were decreased, and genes in black indicate no change in expression in COVID-19 lung tissue relative to normal lung tissue. An increase in SARS-CoV-2 RNA in lung tissue is indicated by a purple gradient bar with values ranging from 0 to 44.9. Viral *C*_T_ values were normalized to the calibrator gene GAPDH, and final *C*_T_ values were inverted (40 − Δ*t*) such that a higher value represents a higher viral load. (**E**) Basal cell and lung repair–related proteins in plasma from patients with COVID-19 (*n* = 6) and healthy volunteers (*n* = 10) were measured by the Olink platform. Values were statistically significant (*P* < 0.05) between the groups using standard *t* test with Benjamini-Hochberg correction. Scale of normalized protein expression (NPX) is log_2_. Each circle represents an individual plasma sample, and 25th, median, and 75th quartiles are indicated with box-and-whisker plots. SCF, stem cell factor. (**F**) Shown is a gene ontology analysis of transcripts with expression correlating (*R* ≥ 0.6) with pulmonary SARS-CoV-2 RNA levels. Scale bars, 250 μm (A) and 100 μm (C). NF-κB, nuclear factor κB; JAK, Janus kinase; STAT, signal transducer and activator of transcription; IRF, IFN regulatory factor.

The postmortem lung sections from all 18 cases showed DAD as the predominant histopathologic finding, ranging from acute DAD through organizing and late (fibrotic)–stage DAD (fig. S3 and table S11). Acute DAD was the predominant histopathology observed in cases with the shortest recorded SOTDs (<10 days). Histopathologic features associated with acute DAD included widespread pulmonary edema, fibrin deposition, hyaline membrane formation, and frequently a marked neutrophilic infiltrate within alveolar interstitial spaces and to a lesser extent within the airspaces (Fig. 1A and fig. S3, A to D). Lung alveolar cell desquamation was often observed (Fig. 1A). Respiratory bronchial and bronchiolar epithelia were frequently denuded, including loss of basal cell populations, and necrotic respiratory epithelial cells were frequently observed within alveolar air spaces (fig. S3E). Cases with an intermediate SOTD (about 10 to 20 days) often showed features of organizing DAD with hyperplasia of AT2 cells and reactive changes (fig. S3F), initial fibrotic expansion of the interalveolar septa, and foci of squamous metaplasia and other reactive changes to remaining respiratory epithelia (fig. S3G). Cases with longer SOTDs (>20 days) showed increasing amounts of pulmonary fibrosis, loss of alveoli, and proliferation of fibroblasts and macrophages (fig. S3, H and I). Widespread thrombosis was seen in most of the cases, ranging from medium-sized vessels to capillary microthrombi (fig. S3, J to L).

To localize pulmonary sites of viral infection, serial lung sections were stained with hematoxylin and eosin (H&E) and were immune-labeled for SARS-CoV-2 nucleocapsid protein (NP). Prominent NP staining was mainly detected in patients with shorter clinical illness (SOTD <10 days) (Fig. 1, A and B, and fig. S4). These cases typically showed acute DAD with extensive hyaline membranes, thick alveolar septa, and intra-alveolar epithelial debris and infiltrates (Fig. 1A). Pronounced NP deposition was observed in hyaline membranes and intra-alveolar cellular debris (Fig. 1A and fig. S4). In cases with a short SOTD, high viral antigen deposition was identified by immunohistochemistry in AT1 and AT2 cells, bronchiolar epithelial cells (including respiratory epithelial basal cells) (Fig. 1B), and, less frequently, in the cytoplasm of vascular endothelial cells and adjacent pericytes of medium and small blood vessels in cases with the highest viral load (Fig. 1C and fig. S5). Endothelial cell staining while suggestive does not definitively indicate productive viral infection of these cells.

To characterize host responses, postmortem lung tissues were analyzed by expression microarray analysis, and antemortem plasma samples were analyzed by multiplexed protein assays. Measurement of gene expression responses in autopsy lung tissue from individual patients with COVID-19 (*n* = 13) was compared to expression in a pool of RNA from normal healthy human lung tissue (*n* = 3). Detection of NP in airway basal cells, which play key roles in repair and homeostasis of the respiratory epithelium (28), suggests that basal cells may be early targets of cytopathic viral infection. Basal cell self-renewal and terminal differentiation are dependent on fibroblast growth factor (FGF) receptor 2 (FGFR2)/SOX2 (29) and bone morphogenetic protein 4 (BMP4)-nuclear factor of activated T cells 1 (NFATc1)-thrombospondin-1 signaling (30). Expression of *NFATc1*, *THBS1*, *FGF7*, and *FGF10* mRNAs, which are known to activate *FGFR2* (31), was increased in lung tissue from patients with COVID-19 (Fig. 1D). However, *FGFR2* and *SOX2* transcripts were decreased, as were transcripts of other genes involved in stem cell renewal and differentiation (*BMP4* and *BMPR1A*) (30). Expression of ITGA6, a cell surface marker of lung basal cells (28, 32), was decreased at both the mRNA level in lung (Fig. 1D) and at the protein level in plasma (Fig. 1E). Plasma concentrations of FGF2 and stem cell factor, required for lung repair (31, 33), were also decreased in patients with COVID-19 (Fig. 1E). Plasma concentrations of amphiregulin, an epidermal growth factor produced by damaged epithelial cells that promotes lung repair (34), were elevated in patients with COVID-19 (Fig. 1E).

To characterize the effect of SARS-CoV-2 viral load on host gene expression in lung tissue, correlation analysis (Pearson coefficient ≥ 0.6) was performed using S protein mRNA qRT-PCR cycle threshold (*C*_T_) values. This analysis identified transcripts (*n* = 368) with expression that positively correlated with SARS-CoV-2 viral load (fig. S6). Principal component analysis of these transcripts showed that, in general, patients with COVID-19 with higher viral loads tended to have a shorter SOTD (fig. S7), consistent with a previous report (35). Gene ontology analysis of these sequences revealed enrichment for processes related to innate immune responses and antiviral responses, including interferon (IFN) signaling, IFN regulatory factor activation, Janus kinase/signal transducer and activator of transcription signaling, and pattern recognition receptors (Fig. 1F). Expression of genes encoding type I and III IFNs, including *IFNA4*, *IFNA5*, *IFNA10*, *IFNA21*, *IL28*, and *IL29*, and other transcripts associated with IFN signaling was increased in lung tissue from patients with COVID-19 compared to normal healthy control lung tissue (fig. S8A). More highly expressed transcripts in patients with higher viral loads were enriched for inflammatory [inter-leukin 8 (IL8)/IL6 signaling, acute phase response signaling, nuclear factor κB signaling, and dendritic cell function] and lymphocyte responses (B cell signaling) (Fig. 1F). Multiplex measurements of immune response proteins in antemortem plasma samples from patients with COVID-19 (*n* = 6 patients sampled up to four times before death; *n* = 11 samples total) demonstrated that concentrations of IFN-related proteins IFNLR1, DDX58, and leukemia inhibitory factor were significantly (*P* < 0.05) elevated compared to normal healthy volunteers (*n* = 10) (fig. S8B). Correlation analysis also identified transcripts (*n* = 450) that negatively correlated with viral load (fig. S9A). These transcripts with reduced expression were mainly enriched for processes associated with cellular metabolism, including carbohydrate/lipid metabolism and molecular transport (fig. S9B). Processes related to respiratory function also negatively correlated with viral load, including regulation of surfactant production from AT2 cells. Collectively, these data suggest that even though lung repair processes are being activated, key elements appear to be impaired through damage and loss of critical lung basal cell populations, revealing a mechanism for impaired lung tissue repair in these patients with COVID-19.

### Fatal SARS-CoV-2 infection leads to damage at the lung alveolar-capillary barrier

**Fig. 2.**
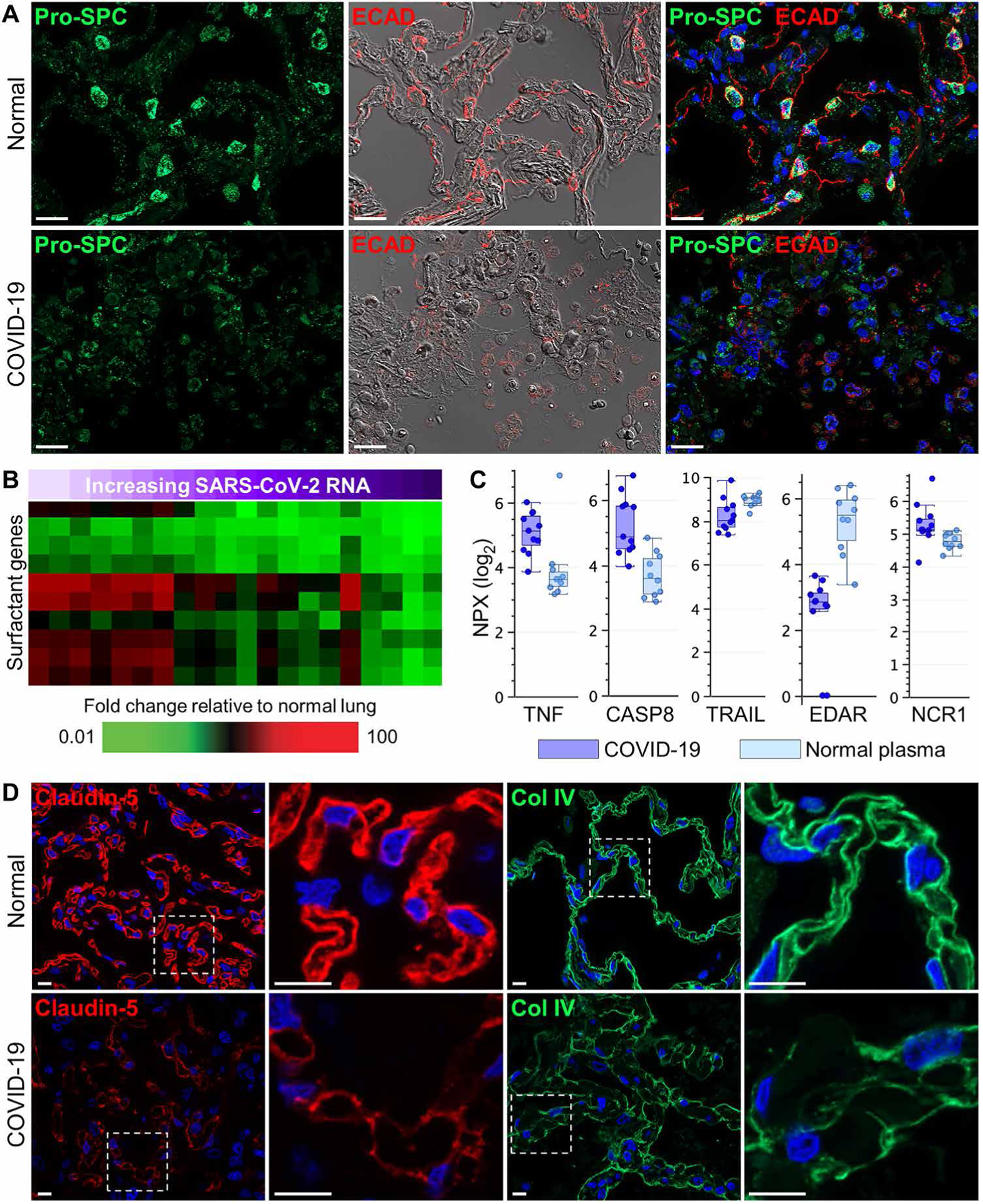
Alveolar epithelial and endothelial damage in COVID-19 lung autopsy samples. (**A**) Representative immunofluorescence and differential interference contrast (DIC) images showing prosurfactant protein C (Pro-SPC) and E-cadherin (ECAD) expression in a COVID-19 lung autopsy sample (case 1) and in normal lung tissue. Normal lung tissue alveolar septa show prominent Pro-SPC and E-cadherin expression in lung AT2 cells and epithelial junctions, respectively, compared to COVID-19 lung tissue which shows marked loss of alveolar septal Pro-SPC and E-cadherin staining and intra-alveolar accumulation of positive-stained epithelial debris. (**B**) Heatmap shows expression of genes encoding lung surfactant proteins that show differential expression in COVID-19 lung tissue samples (*n* = 13 cases; table S5) compared to normal lung tissue. SARS-CoV-2 RNA in lung tissue is indicated by a purple gradient bar with values ranging from 0 to 44.9. Viral *C*_T_ values were normalized to the calibrator gene GAPDH, and final *C*_T_ values were inverted (40 − Δ*t*) such that a higher value represents a higher viral load. (**C**) Cell death–related proteins in plasma from patients with COVID-19 (*n* = 6) and healthy volunteers (*n* = 10) were measured by Olink platform. Values were statistically significant (*P* < 0.05) between the groups using standard *t* test with Benjamini-Hochberg correction. Scale of NPX is log_2_. Each circle represents an individual plasma sample, and 25th, median, and 75th quartiles are indicated with box-and-whisker plots. (**D**) Shown are representative immunofluorescence images of alveolar capillary claudin-5 expression and alveolar basement membrane collagen type 4 (Col IV) expression in normal lung tissue and COVID-19 lung tissue (case 12). Digitally magnified boxed areas highlight the reduced and discontinuous staining of claudin-5 and Col IV in the COVID-19 lung tissue compared to normal lung tissue. Scale bars, 20 μm (A) and 10 μm (D).

To examine the integrity of the alveolar-capillary barrier, lung sections were immunostained for prosurfactant protein C (Pro-SPC), a marker of AT2 cells, and E-cadherin, a major epithelial intercellular junction protein. Reduced Pro-SPC and E-cadherin staining was observed in the alveolar septa of lung tissue from patients with COVID-19 relative to normal lungs, particularly in patients with a short SOTD (Fig. 2A and fig. S10, A and B). Expression of Pro-SPC, E-cadherin, and cytokeratin in cases with intermediate and longer clinical illness showed abnormal distribution with elevated expression in epithelial hyperplastic and bronchiolar metaplastic lesions (figs. S3, F and G, and S11). The expression of genes encoding surfactants A, B, C, and D was decreased in lung tissue from patients with COVID-19 and correlated significantly (≥0.6) with viral load, with greater loss of expression observed in patients with higher viral loads (Fig. 2B). Surfactants, produced by AT2 cells, maintain normal lung function by lowering surface tension at air-liquid interfaces thereby preventing alveolar collapse, and critically contribute to pathogen defense and immune modulation (36). Loss of surfactant gene expression is likely the result of viral infection and destruction of AT2 cells. In support of histological evidence of lung epithelium damage, concentrations of cell death–related proteins tumor necrosis factor–α (TNFα), caspase 8, TRAIL, EDAR, and NCR1 were altered in plasma from patients with COVID-19 relative to normal controls (Fig. 2C). The antemortem plasma samples available represented patients with both a range of clinical illness and a range in sampling time points relative to symptom onset. However, the small sample size (*n* = 6 patients) made it difficult to identify potential differences due to these variables.

To further evaluate alveolar-capillary barrier integrity, lung sections were immunostained for claudin-5, an endothelial junction protein, and collagen type 4 (Col IV), the main collagen component of the alveolar basement membrane. Representative immunofluorescence analyses of normal lungs showed robust claudin-5 and Col IV labeling along the borders of alveolar capillary loops and the thin and thick portions of the alveolar basement membrane, respectively (Fig. 2D). In contrast, lung tissue from patients with COVID-19 showed severely diminished and discontinuous claudin-5 and Col IV immunoreactivity. Microarray analyses also identified reduced transcripts encoding endothelial junction proteins and adhesion molecules, including *ENG*, *PECAM1*, *VCAM1*, and *MCAM* as well as increased transcription of the gene encoding angiopoietin-2, a negative modulator of endothelial barrier dysfunction (fig. S12). Together, these data show that SARS-CoV-2 infection disrupts critical alveolar epithelial and endothelial function leading to breakdown of the alveolar-capillary barrier.

### Progressive DAD histopathology and neutrophil responses

**Fig. 3.**
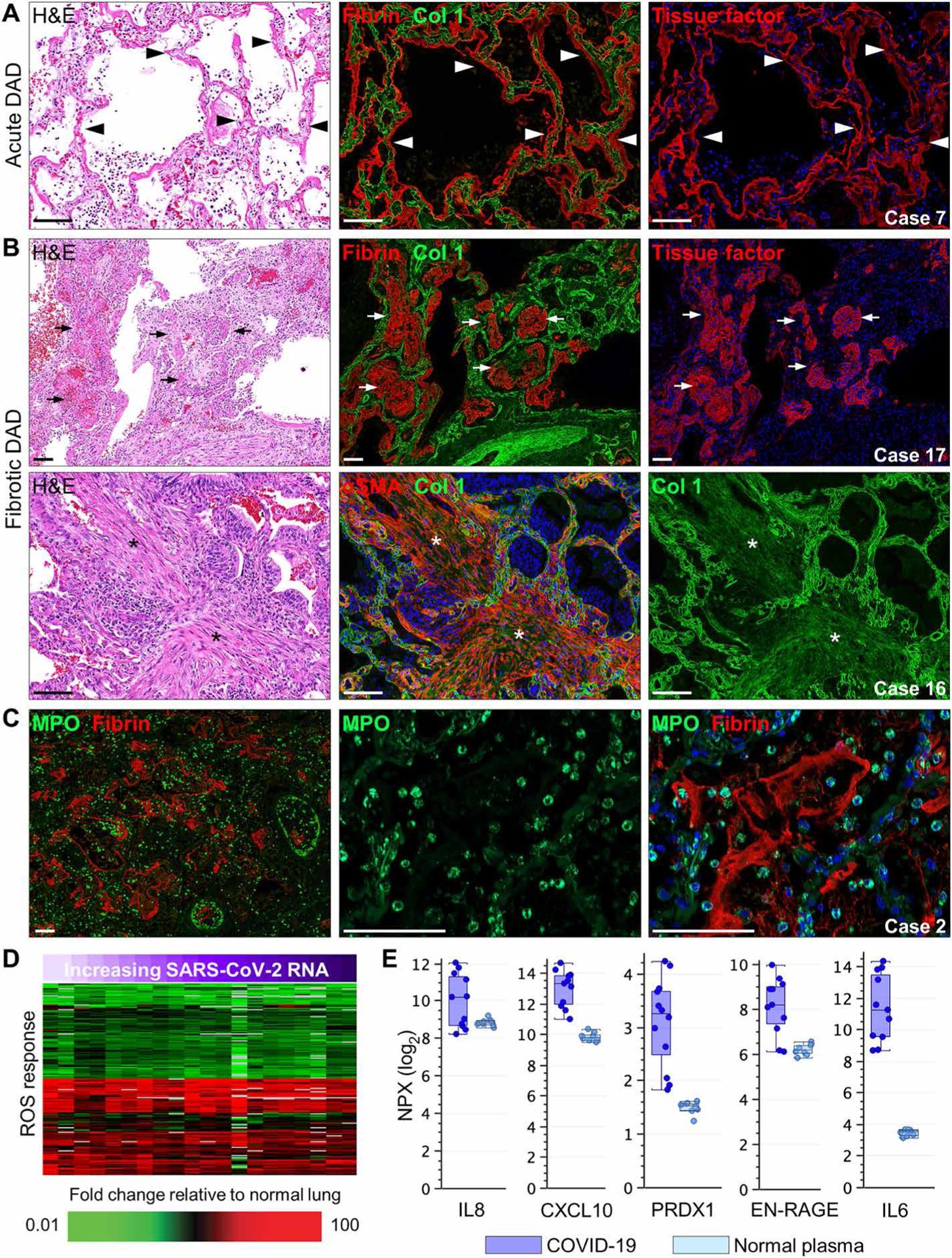
Progressive DAD histopathology and neutrophil responses in COVID-19 lung autopsy samples. (**A** and **B**) Representative lung sections show immunostaining of (A) acute and (B) fibrotic DAD in COVID-19 lung tissue (cases 7, 16, and 17). For (A) and (B), serial lung sections were stained with H&E or were immunostained for fibrin, collagen type 1 (Col 1), tissue factor, or αSMA. Black/white arrowheads in (A) showing acute DAD lung tissue indicate hyaline membrane formation with marked colocalization of fibrin and tissue factor lining alveolar spaces. Black/white arrows in (B) showing fibrotic DAD lung tissue depict late lesions consisting of colocalized intra-alveolar fibrin and tissue factor in “fibrin balls” within alveolar spaces. Black/white asterisks show areas of interstitial fibrosis with marked colocalized expression of αSMA and Col 1. (**C**) Representative immunofluorescence staining of neutrophil myeloperoxidase (MPO) and fibrin in a COVID-19 autopsy lung tissue sample with histological evidence of acute DAD (case 2). (**D**) Heatmap presents expression of genes associated with the ROS response that show differential expression in COVID-19 lung tissue samples (*n* = 13 cases; table S5) compared to normal lung tissue. SARS-CoV-2 RNA in lung tissue is indicated by a purple gradient bar with values ranging from 0 to 44.9. Viral *C*_T_ values were normalized to the calibrator gene GAPDH, and final *C*_T_ values were inverted (40 − Δ*t*) such that a higher value represents a higher viral load. (**E**) Measurement of proteins associated with neutrophil infiltration and oxidative stress in plasma from patients with COVID-19 (*n* = 6) and healthy volunteers (*n* = 10) was performed using the Olink platform. Values were statistically significant (*P* < 0.05) between the groups using standard *t* test with Benjamini-Hochberg correction. Each circle represents an individual plasma sample, and 25th, median, and 75th quartiles are indicated with box-and-whisker plots. Scale of NPX is log_2_. Scale bars, 100 μm.

To further characterize histological indices of DAD progression in relation to clinical illness duration, serial lung sections were stained with H&E or for fibrin, tissue factor, collagen type 1 (Col 1), and α–smooth muscle actin (αSMA). Acute DAD was characterized by marked deposition and colocalization of intra-alveolar fibrin and tissue factor in hyaline membranes (Fig. 3A and fig. S3, A to D). In these cases, Col 1 staining was increased in alveolar interstitial spaces, consistent with the observed septal wall thickening. Cases with intermediate illness duration showed less widespread intra-alveolar fibrin and tissue factor expression but were associated with widespread AT2 cell hyperplasia, a hallmark of organizing DAD (figs. S3F and S11, A and C). Cases with longer illness duration displayed two distinct histopathological patterns expressed either individually or together within the same lung specimen. The first pattern featured multiple colocalized fibrin and tissue factor patches or fibrin “balls” in alveolar spaces that also expressed Col 1 but not αSMA (Fig. 3B). The second pattern featured diffuse interstitial fibrosis with marked expression and colocalization of αSMA and Col 1 (Fig. 3B). Pulmonary vascular remodeling with excessive vessel wall–associated αSMA and Col 1 expression was another prominent finding in intermediate and long-duration cases (fig. S13).

Lung sections were immunostained for myeloperoxidase (MPO), an enzyme primarily expressed in the azurophilic granules of neutrophils. Cases with short SOTDs showed extensive accumulation of MPO-positive neutrophils either trapped in alveolar septal capillaries or extravasated into fibrin-filled alveolar spaces (Fig. 3C and fig. S14). Marked neutrophil adhesion and margination were often detected along the inner endothelial lining of many large- and medium-sized blood vessels (Fig. 3C). In longer-duration cases, fewer MPO-positive neutrophils were generally detectable, and these cells were primarily found in alveolar fibrin “balls” but not in the interstitial fibrotic areas. A subset of neutrophils showed increased nuclear expression of citrullinated histone H3 (CitH3), an early indicator of neutrophil extracellular trap (NET) formation (fig. S15A). These CitH3-positive neutrophils were predominantly located in alveolar spaces and were more widespread in lungs from cases with short-duration disease (fig. S15B). Released NETs costaining for CitH3, MPO, and cell-free DNA were sporadically detected in damaged lung parenchyma, airway lumens, alveolar spaces, and platelet-rich thrombi (fig. S15, C to E). Consistent with this neutrophil response, differential expression of genes associated with neutrophil infiltration and activation was observed in lung tissue (fig. S16). Transcripts associated with the reactive oxygen species (ROS) response were also modulated in lung tissue (Fig. 3D). Plasma proteins associated with neutrophil and ROS responses including IL8, CXCL10, and EN-RAGE primarily derived from activated neutrophils (37), IL6, and PRDX1 were also elevated in lung tissue from patients with COVID-19 (Fig. 3E). Statistical analysis was performed to identify the relationships between DAD progression and other clinical and histological data. As shown in fig. S17, a positive correlation was calculated between acute DAD and SARS-CoV-2 viral RNA load [correlation coefficient (*R*) = 0.565, *P* = 0.024], fibrotic DAD with age (*R* = 0.565, *P* = 0.015), and fibrotic DAD with SOTD (*R* = 0.555, *P* = 0.017). A negative correlation was calculated between acute DAD and fibrotic DAD (*R* = −0.76, *P* = 0.00026) and SOTD with SARS-CoV-2 RNA viral load (*R* = −0.722, *P* = 0.001).

### Prominent pulmonary fibrosis and macrophage activation associated with longer SOTD

**Fig. 4.**
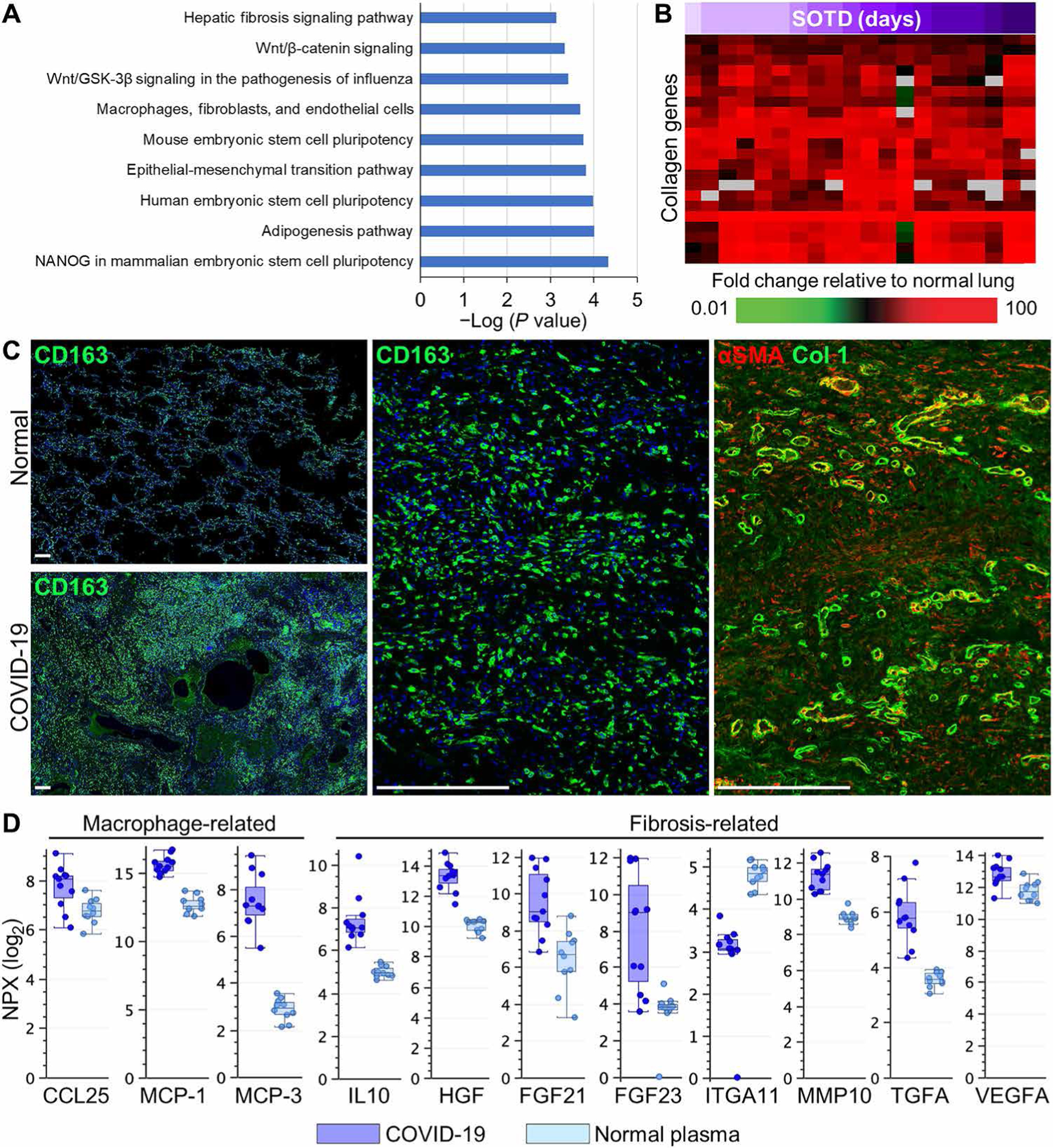
Macrophage activation and fibrogenesis in COVID-19 lung autopsy samples. (**A**) Gene ontology analysis of transcripts with expression correlating (*R* ≥ 0.6) with time of symptom onset to death (SOTD). (**B**) Heatmap shows expression of genes encoding collagen proteins that show differential expression in COVID-19 lung tissue samples (*n* = 13 cases; table S5) compared to normal lung tissue. SOTD is indicated by a purple gradient bar. (**C**) Representative immunofluorescence staining of macrophage CD163 or αSMA and collagen type 1 (Col 1) in COVID-19 lung tissue with histological evidence of fibrotic DAD (case 18). Low-power images highlight the increased patchy distribution of CD163-positive macrophages in regions undergoing fibrosis in the COVID-19 lung section compared to lower CD163 expression in normal lung tissue. High-power images of serial sections depict interstitial fibrotic areas filled with CD163-positive macrophages that colocalize with diffuse deposition of αSMA and Col 1 surrounded by brightly stained αSMA- and Col 1–positive blood vessels. (**D**) Measurement of proteins associated with macrophage infiltration and fibrogenesis in plasma from patients with COVID-19 (*n* = 6) and healthy volunteers (*n* = 10) using the Olink platform. Values were statistically significant (*P* < 0.05) between the groups using standard *t* test with Benjamini-Hochberg correction. Scale of NPX is log_2_. Each circle represents an individual plasma sample, and 25th, median, and 75th quartiles are indicated with box-and-whisker plots. Scale bars, 250 μm.

Pearson correlation analysis using days of SOTD was performed to understand the relationship between lung gene expression changes, histopathology, and clinical illness duration. This analysis identified transcripts that positively (*n* = 263) and negatively (*n* = 333) correlated with clinical illness duration (fig. S18, A and B). Gene ontology analysis of transcripts with increased expression in longer duration cases revealed enrichment of processes linked to fibrosis and wound repair, including fibrosis signaling, epithelial-mesenchymal transition, Wnt and stem cell–related signaling, adipogenesis, as well as macrophage, fibroblast, and endothelial cell function (Fig. 4A). Consistent with gene ontology analysis, there was increased expression of genes encoding numerous collagens, including some (e.g., Col11A1 and Col14A1) that showed a positive correlation with SOTD (Fig. 4B). Immunofluorescence analyses identified prominent CD163-positive macrophage accumulation in cases with extensive fibrotic DAD compared to lower CD163 expression in normal lungs and acute DAD cases (Fig. 4C and fig. S19). Notable perivascular CD163-positive staining was observed around large vessels and in fibrotic interstitial areas containing elevated deposition of αSMA and Col 1 (Fig. 4C). Plasma concentrations of macrophage chemotactic proteins (MCPs) (CCL25, MCP-1, and MCP-3) and key fibrosis mediators [IL10, hepatocyte growth factor, FGF21, FGF23, matrix metalloproteinase 10 (MMP10), transforming growth factor–α (TGFα), and vascular endothelial growth factor] were elevated in patients with COVID-19, whereas integrin subunit alpha 11 (ITGA11) was decreased (Fig. 4D). ITGA11 selectively binds to Col 1 during fibrogenesis (38); thus, the decrease in plasma ITGA11 may reflect the increased binding of ITGA11 to Col 1 in fibrotic lung tissue.

### Dysregulation of fibrinolysis and failure of normal clot resolution in severe COVID-19

**Fig. 5.**
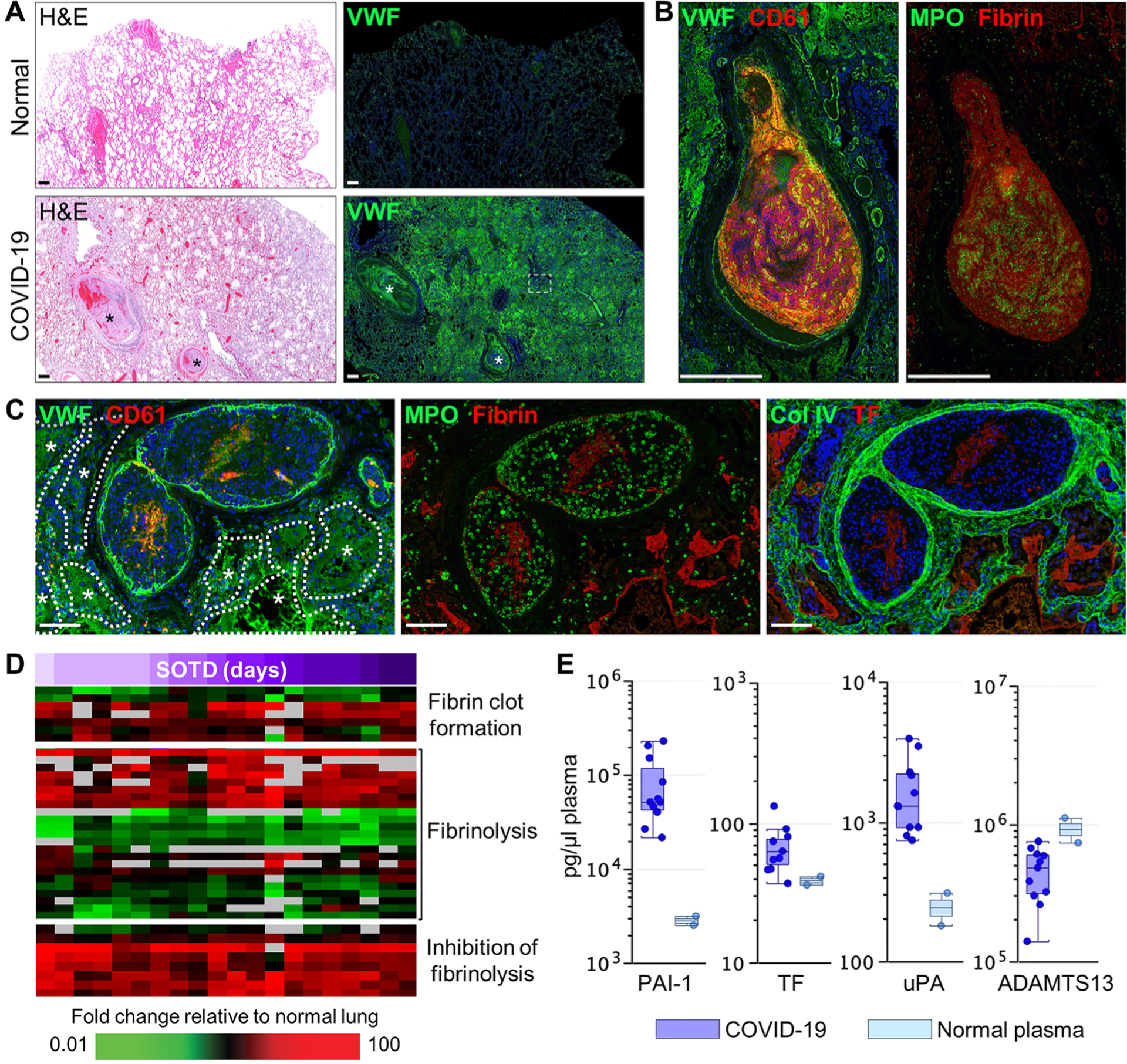
Vascular injury and mediators of coagulopathies in COVID-19 lung autopsy samples. (**A**) Serial sections of normal and COVID-19 (case 7) lung tissue were stained with H&E or immunostained for von Willebrand Factor (VWF). Widespread intra- and extravascular VWF staining is observed in the COVID-19 lung section including large blood clots with high VWF expression (asterisks). (**B**) Large blood clots [region indicated by white asterisks in (A)] show marked expression of VWF, platelet CD61, fibrin, and neutrophil MPO. (**C**) Medium-sized blood clots [white boxed region in (A)] show endothelial- and clot-associated VWF and CD61, MPO and fibrin, and tissue factor (TF) and Col IV. Asterisks and dotted white outlines depict intra-alveolar spaces flooded with extravascular VWF. (**D**) Heatmap shows expression of genes associated with coagulopathy with differential expression in COVID-19 lung tissue samples (*n* = 13 cases; table S5) compared to normal lung tissue. Time (days) of SOTD is indicated by a purple gradient bar. (**E**) Measurement of coagulation-related proteins in plasma from patients with COVID-19 (*n* = 6) and healthy volunteers (*n* = 10) was performed by multiplex enzyme-linked immunosorbent assay. Each circle represents an individual plasma sample, and 25th, median, and 75th quartiles are indicated with box-and-whisker plots. Scale bars, 500 μm (A and B) and 100 μm (C).

Inflammation and endothelial injury promote coagulopathies, a common pathophysiological finding in severe COVID-19. Von Willebrand factor (VWF), a large multimeric glycoprotein synthesized, stored, and released by endothelial cells and platelets, is a key initiator of clotting reactions. Representative immunofluorescence analyses revealed prominent intravascular and parenchymal VWF immunoreactivity in lung tissue from patients with COVID-19 compared to normal lungs (Fig. 5A and fig. S20, A and B). Large- and medium-sized thrombi containing intense VWF staining colocalized with CD61-positive platelets, MPO-positive neutrophils, and fibrin (Fig. 5, B and C). Marked CitH3 staining was also observed in these platelet-rich thrombi but not in platelet-poor thrombi (fig. S15, D and E). Vessels containing clots typically showed reduced and discontinuous staining for endothelial junction markers and were more frequently observed in short and intermediate SOTD cases than in long-duration cases. Marked VWF extravasation into alveolar spaces was also observed, potentially because of alveolar-capillary barrier breakdown (Fig. 5, A to C). Consistent with histological evidence of clot formation with increased staining of VWF and fibrin, expression of genes associated with fibrin clot formation (*FN1*, *FGA*, and *F13A1*) was also increased in lung tissue from patients with COVID-19 (Fig. 5D). Decreased plasma concentrations of ADAMTS13, the natural metalloprotease inhibitor of VWF, were also observed (Fig. 5E) (38). Plasma concentrations of tissue factor, the major activator of the extrinsic coagulation pathway, and urokinase plasminogen activator (uPA), which mediates cleavage of plasminogen to plasmin that then breaks down fibrin clots, were also elevated (Fig. 5E).

**Fig. 6.**
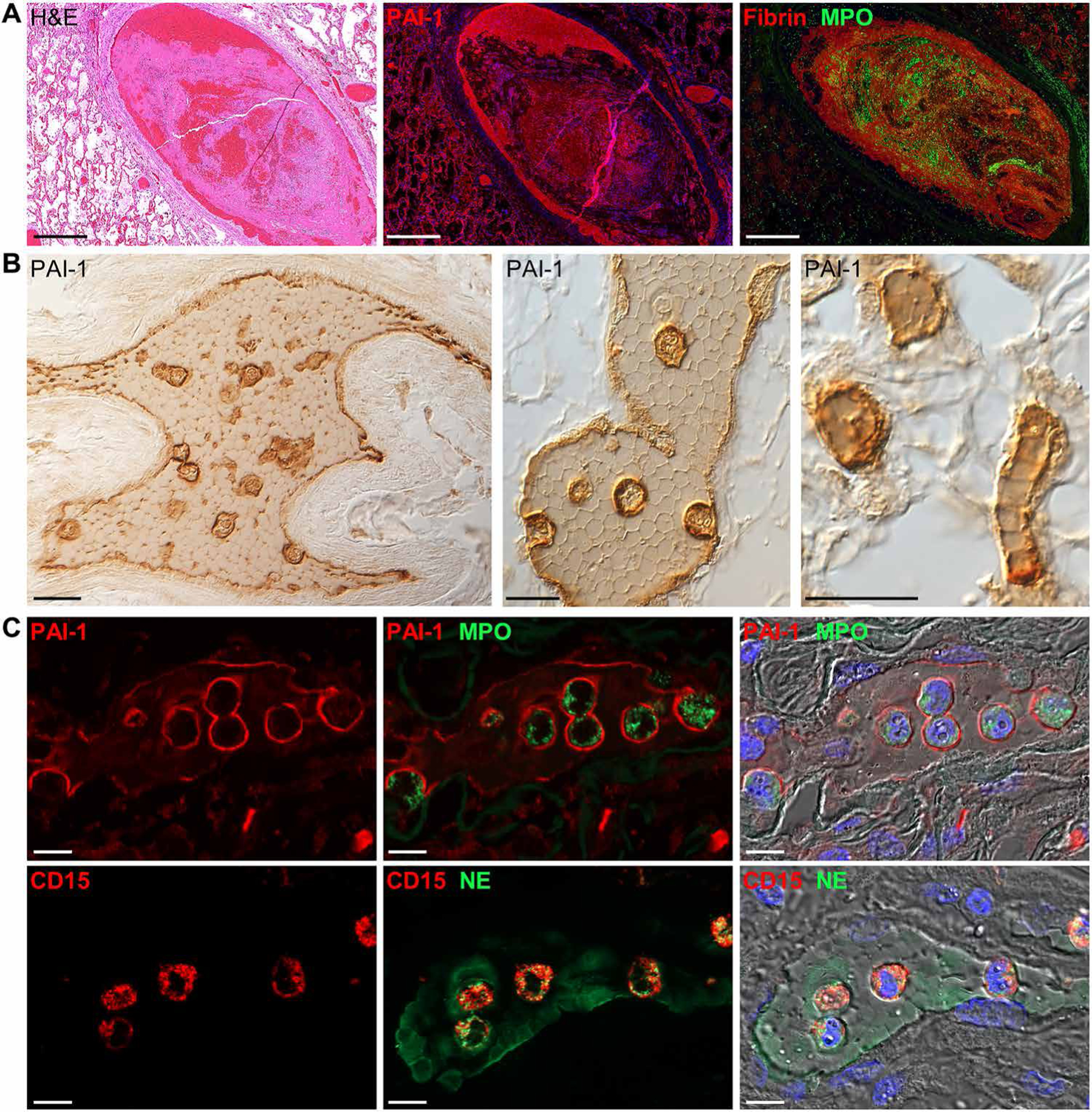
Vascular- and clot-associated PAI-1 expression in COVID-19 lung autopsy samples. (**A**) Representative images of a pulmonary clot in lung tissue from a patient with COVID-19 (case 7). Serial lung sections were stained with H&E or were immunostained for PAI-1 or fibrin and MPO. Marked PAI-1 expression was observed along the inner endothelial lining and colocalized with fibrin-rich regions of the clot. (**B**) Representative immunohistochemical images of clot-associated PAI-1 expression in a medium-sized pulmonary artery (left), small-sized pulmonary vein (middle), and alveolar capillaries (right) (case 10). PAI-1 was detected on vessel endothelium and was lightly expressed between tightly packed polyhedrocyte-shaped red blood cells and markedly expressed on clot-embedded cells. (**C**) Shown are immunofluorescence and DIC images of clot-embedded cells expressing PAI-1 and the neutrophil markers, MPO, neutrophil elastase (NE), and CD15. Nuclei were counterstained with Hoechst 33342 dye (blue). Scale bars, 500 μm (A) and 10 μm (B and C).

Activation of coagulation in fatal COVID-19 also appears to coincide with the inhibition of fibrinolysis in the lung. Whereas some genes associated with fibrinolysis (*F12* and *ANXA2*) showed increased expression, many were decreased in COVID-19 lung tissue relative to normal tissue (Fig. 5D). Expression of genes encoding key inhibitors of fibrinolysis, including *SERPINE1* [PAI-1 (plasminogen activator inhibitor-1)], *SERPINF1* (A2AP), *THBS1* (thrombospondin 1), and *HRG* (histidine-rich glycoprotein) were elevated relative to normal lung tissue regardless of clinical illness duration (Fig. 5D). Plasma concentrations of PAI-1 were also increased in patients with COVID-19 (Fig. 5E). Immunofluorescence analyses revealed high PAI-1 immunoreactivity embedded in large fibrin- and MPO-positive blood clots (Fig. 6A). Prominent PAI-1 staining was also detected on the endothelium of alveolar septal capillaries and in small- and medium-sized blood vessels containing unresolved clots with tightly packed polyhedrocyte-shaped red blood cells and distinctive entrapped PAI-1–positive cells (Fig. 6, A and B). These clot-embedded PAI-1 cells stained positively for neutrophil markers (MPO, neutrophil elastase, and CD15) (Fig. 6C) but were negative for markers of T cells (CD3), B cells (CD20), monocytes/macrophages (CD14/CD163), or megakaryocytes/platelets (CD61/CD42b) (fig. S21). These PAI-1–labeled neutrophils were also negative for cleaved caspase 3 and CitH3, suggesting a nonapoptotic and non–NET-producing phenotype, respectively (figs. S21 and S15D). Endothelial and inflammatory cell expression of IL6 and TGFβ1 in pulmonary blood vessels and alveolar septal capillaries also paralleled the localization of PAI-1 (fig. S22A). Colocalized staining of PAI-1 and TGFβ1 was also detected in developing fibrotic lesions (fig. S22, B and C). Together, these data indicate that COVID-19 coagulopathies may be driven by an imbalance in the regulation of prothrombotic and anti-fibrinolytic processes.

### Increased cellular senescence of epithelium and endothelium in COVID-19 lungs

**Fig. 7.**
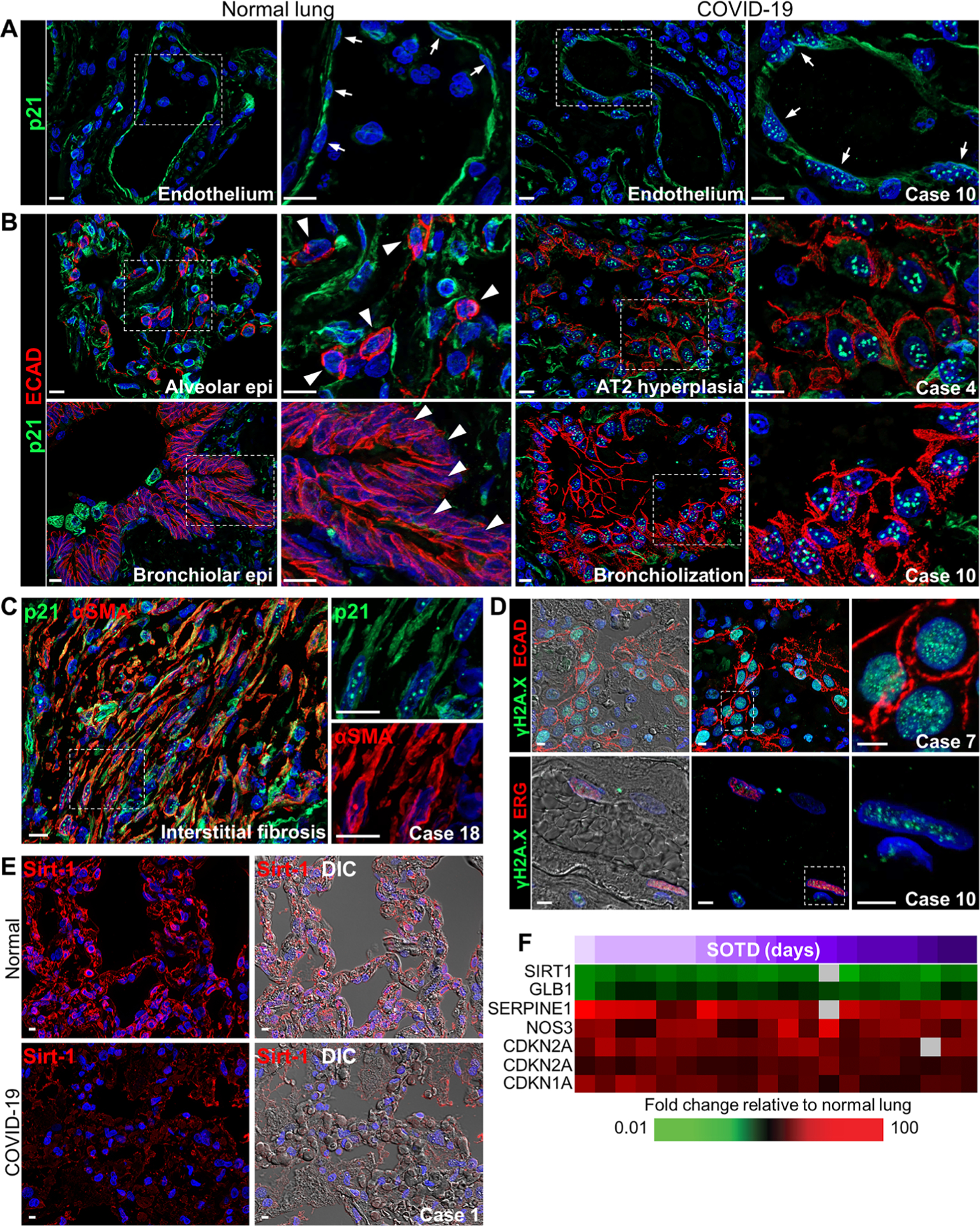
Expression of cellular senescence markers in COVID-19 lung autopsy samples. (**A** and **B**) Representative images show p21 staining in (A) vascular endothelium and (B) alveolar and airway epithelium in normal lung tissue and COVID-19 lung tissue (cases 4 and 10). (A) Distinctive punctate nuclear p21 staining is observed in endothelial cells in the COVID-19 lung section but not in the normal lung section (white arrows). (B) Punctate p21 nuclear staining is seen in E-cadherin–labeled epithelial cells in abnormal hyperplastic and bronchiolization lesions in COVID-19 lung tissue. White arrowheads depict the lack of nuclear p21 staining in normal lung AT2 cells and bronchiolar epithelial cells. (**C**) Representative immunofluorescence images show colocalized staining of p21 and α–smooth muscle actin (αSMA) in cells forming interstitial fibrotic lesions in lung tissue from a patient with COVID-19 (case 18) with a longer SOTD. White dotted box shows area of digital enlargement. (**D**) Immunofluorescence and DIC images show nuclear γH2A.X foci in epithelial (top) and endothelial (bottom) cells costained for E-cadherin or the erythroblast transformation specific (ETS)–related gene (ERG) in COVID-19 lung tissue (cases 7 and 10). (**E**) Representative immunofluorescence and DIC images show sirtuin-1 (Sirt-1) expression in the alveolar septa of a normal lung section and a COVID-19 lung section (case 1). (**F**) Heatmap shows expression of genes encoding senescence markers with differential expression in COVID-19 lung tissue samples (*n* = 13 cases; table S5) compared to normal lung tissue. Time (days) of SOTD is indicated by a purple gradient bar. Scale bars, 10 μm (A to C) and 5 μm (D and E). Epi, epithelium.

Collectively, these findings indicate that pulmonary SARS-CoV-2 infection disrupts the balance between normal tissue regeneration and pathological fibrosis, promotes increased and unresolving vascular thrombosis, and elicits production of damaging proinflammatory mediators and ROS. Because many of these dysregulated processes overlap with those that mediate or characterize cellular senescence and age-related pathologies, the potential relationship between cellular senescence and COVID-19 pathogenesis was investigated. Immunofluorescence analyses identified increased nuclear expression of the cyclin-dependent kinase inhibitor p21, a marker of senescence, in vascular endothelium and hyperplastic and metaplastic E-cadherin–labeled epithelial cells in lung tissue from patients with COVID-19 compared to endothelial and epithelial cells in normal lungs (Fig. 7, A and B). Cells expressing nuclear p21 showed minimal staining for the proliferation marker Ki67, providing further evidence of a senescence-associated growth arrest phenotype (fig. S23). Increased nuclear and cytoplasmic p21 expression was also observed in αSMA-positive cells, presumably myofibroblasts, located in interstitial fibrotic foci in lungs from COVID-19 cases with longer illness duration (Fig. 7C). Injured or senescent epithelial and endothelial cells also showed elevated nuclear expression of phosphorylated histone H2A.X (γH2A.X), a marker of ROS-induced DNA damage, in lung tissue from patients with COVID-19 (Fig. 7D). Reduced alveolar septal expression of sirtuin-1 (Sirt-1), a nicotinamide adenine dinucleotide–dependent histone deacetylase involved in regulating inflammation, cellular senescence, and stress resistance was also observed (Fig. 7E). Gene expression analysis revealed increased expression of genes that promote senescence including *SERPINE1* (PAI-1), *eNOS3*, and the cell cycle inhibitors *CDKN2A* (p16) and *CDKN1A* (p21). Expression of *SIRT1* mRNA, a major inhibitor of oxidative stress–induced senescence in endothelial and epithelial cells as well as stem cells, was also decreased (Fig. 7F). Together, these data support a close link between cellular senescence processes, oxidative stress, pulmonary epithelial and endothelial damage, and defective repair in lung tissue from patients with fatal COVID-19.

## DISCUSSION

This series of lung autopsy samples from patients with fatal SARS-CoV-2 infection showed a wide range of aberrant pulmonary responses to infection that were associated with viral load, immune response, and duration of clinical illness before death. Pulmonary pathologic changes over a wide range of illness durations (SOTDs ranging from 3 to 47 days) were investigated. Although pathologic changes likely occurred asynchronously throughout the lung and each case examined resulted in a fatal outcome regardless of SOTD duration, these observations nevertheless suggested a temporal framework for disease progression that outlines a natural history and pathogenesis of SARS-CoV-2 infection (fig. S17).

Pathological observations indicated that both direct virus-induced cytopathic effects and host inflammatory and immune responses led to early pulmonary epithelial and endothelial injury, alveolar-capillary barrier dysfunction, impaired lung tissue repair processes, and widespread vascular thrombosis with reduced fibrinolysis. Furthermore, in cases with a longer disease duration, disease progression led to excessive pulmonary fibrosis, loss of alveoli, and vascular remodeling. Our study also demonstrated epithelial and endothelial cell senescence in the pathophysiology of COVID-19, consistent with increased susceptibility and severity of COVID-19 in the elderly and comorbid risk populations.

DAD and alveolar-capillary barrier breakdown are prominent pathological features of severe COVID-19 (1–4). This lung autopsy series showed that pulmonary SARS-CoV-2 infection damaged all three major components of the alveolar-capillary barrier as evidenced by the loss of alveolar epithelial and endothelial junction integrity, marked desquamation of lung AT1 and AT2 cells, and disruption of the Col IV–expressing alveolar basement membrane. The observed loss of alveolar basement membrane integrity indicates excessive and persistent damage to the alveolar architecture, which often precedes dysregulated tissue repair processes and fibrosis (16, 17). Increased TNF and caspase 8 concentrations with minimal evidence of caspase 3–dependent apoptosis suggested a role for inflammatory-driven cell death pathways (e.g., necroptosis), as previously reported (39). Alveolar damage was accompanied by the marked early reduction of pulmonary surfactant expression, likely mediated by direct SARS-CoV-2 cytopathic effects on lung AT2 cells. This AT2 cell and surfactant loss may correlate clinically with diminished lung compliance and progressive respiratory failure in severe COVID-19. Cases with the shortest SOTD showed marked viral antigen deposition in alveolar and bronchiolar epithelial cells, including respiratory epithelial basal cells. Cases with a longer SOTD frequently showed denuded respiratory epithelium and a lack of lung tissue repair and regeneration. The acute damage was significant, because basal cells are stem cells that give rise to ciliated and secretory epithelial cells of the pseudostratified respiratory epithelium (40). Loss of basal cells precludes regeneration of the airway epithelium, resulting in sustained respiratory compromise (41). Direct viral infection of basal cells in fatal COVID-19 contrasts with fatal influenza viral infection, where viral replication does not occur in basal cells but associated secondary bacterial infections result in basal cell loss and lack of tissue repair and regeneration (42). Thus, fatal COVID-19 appears to differ from fatal influenza in that direct pulmonary damage due to SARS-CoV-2 infection, and the immune response such damage elicits, is of sufficient severity that it does not require secondary bacterial copathogenesis as seen in influenza.

Findings from this study and others indicate that inflammatory responses serve as a primary driver of the progressive and persistent pathophysiologic features of severe COVID-19 (43–46). In short SOTD cases, alveolar-capillary breakdown coincided with prominent recruitment of MPO-positive neutrophils in damaged alveoli with excessive fibrin and tissue factor deposition, in thickened alveolar interstitial spaces, and along the endothelial lining of injured blood vessels. Activated neutrophils, along with damaged epithelial and endothelial cells, release a host of cytokines, chemokines, proteases, MMPs, and cytotoxic ROS, leading to DNA damage and perpetuation of alveolar destruction and respiratory dysfunction. NETs have been shown to promote tissue factor activation and fibrin deposition, consistent with our observations, suggesting a role for NETs in intra-alveolar thrombosis and the formation or organization of platelet-rich clots (43, 45). The greater staining of CitH3-positive neutrophils in lung tissue from short-duration cases compared to longer-duration cases may indicate a more active contribution of NETs to early pathological processes relative to later disease stages. Intra-alveolar neutrophils and injured alveolar epithelial cells are also key producers of tissue factor, which directly stimulates fibrin deposition in alveolar spaces, hyaline membranes, and fibrotic foci (47, 48).

Prominent pulmonary fibrosis was observed in this lung autopsy series, consistent with previous reports (49–51), and correlated with SOTD and patient age (fig. S17). A spectrum of pulmonary fibrotic disease has been observed in COVID-19 pneumonia from fibrosis associated with organizing pneumonia to severe acute lung injury and widespread fibrosis (52). A systematic review of 45 studies of chest computed tomography images of patients with COVID-19 demonstrated pulmonary fibrosis in 17% of patients, which was attributed to impaired healing after viral infection of lung AT2 cells (53). Correlation analysis of the severity and clinical prognosis of patients with COVID-19 demonstrated that an increase in fibrosis indicators detected at hospital admission, including hyaluronic acid, laminin, and type III procollagen, was predictive of critical illness and poor prognosis (49). This finding suggests that fibrosis may occur independently of mechanical ventilation. Furthermore, patients with severe COVID-19 were found to have a high rate of fibrotic lung function abnormalities at hospital release, highlighting important implications for long-term health complications (54).

Vascular dysfunction and thrombotic complications are hallmark features of COVID-19 (55). The present study revealed evidence of viral antigen staining in pulmonary vascular endothelial cells in cases with short SOTD and high viral loads and a high frequency of thrombi in small- and medium-sized pulmonary blood vessels, particularly in short and intermediate SOTD cases. Long-duration cases commonly showed vascular remodeling in medium and large vessels. Collectively, these observations suggest that aberrant clotting reactions in COVID-19 are driven by an imbalance in prothrombotic and antifibrinolytic processes. This has been previously proposed for COVID-19 and other coronaviral diseases including SARS-CoV-1 and Middle East respiratory syndrome (MERS)–CoV (56). An enhanced prothrombotic environment may result, at least in part, from the prominent intravascular expression of VWF caused by endothelial injury and neutrophil-mediated proinflammatory factors as well as by reduced plasma concentrations of the natural VWF inhibitor ADAMTS13. We also observed increased gene and protein expression of several antifibrinolytic factors including PAI-1, the main inhibitor of tissue-type plasminogen activator (tPA) and uPA. Microvascular endothelium is a main site of PAI-1 production, and the marked incorporation of PAI-1 in abnormal or persistent clots supports a plausible mechanism that may explain poor clot resolution in COVID-19 (57). PAI-1–positive neutrophils were often identified in clotted vessels in this study, although it is not clear whether this reflected PAI-1 production by neutrophils or binding of plasma PAI-1 to the outer membrane of neutrophils. A recent study reported a correlation between elevated PAI-1 plasma concentrations and circulating neutrophils in patients with COVID-19 (58). It may be particularly relevant that risk factors for developing severe COVID-19 such as advanced age, obesity, diabetes, and vascular disease are all associated with increased plasma PAI-1 concentrations (59, 60).

Cellular senescence is defined by a stable growth arrest regulated by a family of cyclin-dependent kinase (CDK) inhibitors including p21 and p16 (61). Increased nuclear p21 expression in hyperplastic and metaplastic epithelial cells as well as endothelial cells was detected in this COVID-19 lung autopsy case series. Senescence-mediated loss of progenitor cell capabilities among AT2 and basal cell populations has been implicated in the impaired reepithelialization of damaged alveoli and airways (62). Endothelial senescence may underlie the enhanced prothrombotic properties of injured endothelium in COVID-19 by shifting the balance between anti- and procoagulant pathways (63). Our findings here also link the induction of epithelial and endothelial senescence to oxidative stress–induced DNA damage and modulation of ROS pathways. Aside from the contribution of neutrophils and other cell types to ROS generation, increased angiotensin (Ang) II concentrations mediated by the actions of SARS-CoV-2 on ACE2 receptor–Ang II signaling may up-regulate ROS and cellular senescence pathways (64). Senescent cells exhibit a senescence-associated secretory phenotype (SASP) characterized by an overproduction of proinflammatory molecules such as IL6, TGFβ, IL8, MCP-1, extracellular matrix remodeling enzymes (MMPs), serine/cysteine proteinase inhibitors (serpins), and tissue inhibitors of metalloproteinases (61). SASP markers were elevated at the protein and gene levels in lung tissue from our COVID-19 autopsy case series, including PAI-1, which is considered both a major marker and mediator of cellular senescence (65). Key clinical risk factors for developing severe COVID-19 such as advanced age, obesity, diabetes, and vascular disease are all characterized by the activation or exacerbation of cellular senescence processes (66).

There are certain limitations to our study. Whereas the present case series allowed for the examination of lung tissue from patients with COVID-19 with short, intermediate, or long clinical illness duration, the overall number of patients was small. Larger autopsy studies will be needed to specifically examine mitigating factors including varying patient demographics, comorbidities, and in-hospital interventions (e.g., mechanical ventilatory support). Another potential concern is related to lung tissue sampling and, specifically, whether the available lung specimens used for imaging and transcriptomic analyses were representative of the whole lung. Last, because all cases in this study were fatal, it is unknown to what extent these pathological processes occur in mild to moderate or hospitalized, nonfatal COVID-19 cases or in cases with long-term sequelae after SARS-CoV-2 infection.

Understanding the natural history and pathogenesis of COVID-19 at the cellular and immunological level can potentially provide clues to different therapeutic interventions at different stages of disease progression. Such interventions could reduce the number of severely ill patients who require intensive care and mechanical ventilation as well as limit the number of individuals who experience long-term impaired lung function. A review of 107 patients with COVID-19 showed that 7 to 13 days after illness onset is a critical stage in this disease (67). Additional studies found that, among patients who developed severe disease, the median time to dyspnea was 5 to 8 days, the median time to ARDS was 8 to 12 days, and the median time to intensive care unit admission was 10 to 12 days after symptom onset (68–70). The time interval between symptom onset and the requirement for critical care may therefore represent a unique therapeutic window in which to counter progressive COVID-19 pathologies.

Because of the complexity of COVID-19 pathology, combination treatment with both direct acting antiviral drugs such as remdesivir and therapeutics that modulate damaging host immune responses is likely needed (71–74). Treatment with corticosteroids, that act on multiple anti-inflammatory pathways (75), has shown some efficacy against COVID-19 (76). More targeted anti-inflammatory drugs, including IFNβ, mavrilimumab (a monoclonal antibody targeting granulocyte-macrophage colony-stimulating factor), modified tetracyclines, and ROS scavengers (e.g., *N*-acetylcysteine), are also being evaluated. ROS scavengers have shown efficacy in influenza (77), which, as noted, has certain clinical and pathologic similarities to COVID-19. Clinical trials have been conducted with the IL6-blocking agent tocilizumab in patients with COVID-19 (78), and clinical studies have demonstrated efficacy in lowering all-cause mortality (79). Others have reported that targeting surfactant deficiency may improve respiratory function in patients with COVID-19 (80, 81). Mesenchymal stem cell (MSC) therapy has also been proposed as a treatment for COVID-19 (82, 83). Clinical trials have shown reduced mortality in patients with influenza A/H7N9 virus–induced ARDS treated with transplanted MSCs, and ongoing trials are currently investigating stem cell therapies for treating patients with COVID-19 (83).

Another critical concern is the resolution of pulmonary fibrosis secondary to severe COVID-19 in surviving patients. Both SARS-CoV-1 and MERS-CoV infections have been associated with long-term fibrotic lung disease (84, 85). Clinical trials specifically targeting fibrosis using pirfenidone (ClinicalTrials.gov identifier: NCT04282902, NCT04607928, and NCT04653831) and LYT-100 (deupirfenidone) (NCT04652518) are currently under way. The antioxidative and anti-inflammatory properties of pirfenidone may also target ROS-mediated and DNA-mediated damage to epithelial and endothelial cell populations. Other inhaled or intravenous fibrinolytic therapies for COVID-19–related alveolar fibrosis and coagulopathies have received considerable attention (e.g., tPA) (86, 87). A small molecule inhibitor of PAI-1, TM5614, is also being tested in hospitalized patients with severe COVID-19 to treat both fibrotic and thrombotic complications (ClinicalTrials.gov identifier: NCT04634799). Efforts have also focused on using antithrombotic therapy such as heparin and low–molecular weight heparins in COVID-19, although an incomplete understanding of thrombogenic risk factors and reports of increased pulmonary hemorrhage in certain patients with COVID-19 have complicated the establishment of optimal anti-coagulation treatments for COVID-19 (88–90). Elevated plasma tissue factor and D-dimer concentrations observed in patients with COVID-19 suggest that these mediators may be useful markers of ongoing endothelial dysfunction and hypercoagulability and warrant further investigation of tissue factor as a possible therapeutic target (ClinicalTrials.gov identifier: NCT04655586). Potential therapeutic approaches targeting cellular senescence to improve epithelial and endothelial cell function include the use of senolytic compounds, such as ABT-263 (Navitoclax), which promote clearance of senescent cells by inhibiting prosurvival pathways (91–93). Manipulation of specific components of the SASP represents another potential therapeutic strategy to counter cellular senescence (93).

In summary, the present study provides insight into the pathological, immunological, and host genetic correlates of progressive pulmonary failure and impaired tissue repair in fatal COVID-19. The data presented here highlight key scientific links between these processes and common comorbidities including age, diabetes, and obesity that may help to define important determinants of disease severity and recovery. A more complete understanding of the specific interplay between these pulmonary responses and cellular senescence–driven risk factors may prove critical in the development of relevant disease markers and urgently needed therapeutics.

## MATERIALS AND METHODS

### Study design

This study was designed to examine pulmonary pathology of lung autopsy samples from 18 patients with fatal SARS-CoV-2 infection by analyzing FFPE lung tissue samples by histology, immunohistochemistry, next-generation sequencing, cytokine analysis, and gene expression profiling. The 18 cases included in this study developed fatal SARS-CoV-2 infections that occurred between March and July 2020. Initial lung histopathology findings for the first eight cases have been reported (5). The remaining cases had postmortem, percutaneous open lung biopsy samples that were fixed and processed as FFPE tissue blocks. For each postmortem lung sample examined, sections were cut for histological and immunohistochemical analysis, and RNA was extracted for host genetic and transcriptomic analysis. Viral sequence analyses were performed using next-generation sequencing, and viral load was quantitated by RT-PCR (Table 1). For six of the California patients, antemortem plasma samples were available for analysis of soluble mediators and serology (Table 1). Deidentified autopsy lung samples and plasma bio-specimens were obtained from fatal COVID-19 cases without the requirement for Institutional Review Board (IRB) review according to National Institutes of Health policy.

For comparative imaging analyses, commercially available and deidentified control FFPE lung specimens were obtained under IRB-approved protocols from a cohort of individuals without COVID-19 (*n* = 10), with a mean age of 60 years (range, 41 to 82 years; four females and six males), who had undergone lobectomy for cancer (OriGene Technologies, Rockville, MD). These lung sections were histologically defined as normal by a certified pathologist using routine H&E staining.

### Lung histology, immunohistochemistry, and immunofluorescence analyses

FFPE lung sections 4 μm thick were dewaxed, rehydrated, stained by H&E for routine histology, and scored for DAD histopathology (see Supplementary Materials and Methods). Immunohistochemistry and immunofluorescence protocols and the list of primary antibodies used in all staining experiments are described in Supplementary Materials and Methods (table S12).

### RNA isolation and complementary DNA synthesis

RNA was extracted from four 5-μm sections, cut from each FFPE lung tissue block, and placed into a 1.5-ml Eppendorf tube. Xylene (1 ml) was added and incubated for 15 min at 50°C (94). Samples were centrifuged and air dried, and 200 μl of digestion buffer was added. Samples were incubated for 15 min at 98°C, and proteinase K (1 mg/ml) was added. Samples were incubated overnight at 50°C. The next day, samples were prepared using the RecoverAll Total Nucleic Acid Isolation Kit (Invitrogen) following the manufacturer’s instructions. Final volume was 60 μl of diethylpyrocarbonate–treated water. Synthesis of first-strand complementary DNA was carried out using the SuperScript Synthesis System following the manufacturer’s instructions (Thermo Fisher Scientific, Waltham, MA).

### Quantitation of pulmonary SARS-CoV-2 viral RNA

SARS-CoV-2 RNA levels in human lung tissue was quantified using TaqPath 2019nCoV Assay Kit v1 (Thermo Fisher Scientific). Total RNA samples were adjusted to 4 ng/μl, and then 2.5 μl was combined directly with 4× TaqPath 1-Step RT-qPCR Master Mix, 20× 2019-nCoV Assay either N protein, S protein, or ORF1ab, and 20× ribonuclease P internal-positive control assay. RT and real-time PCR was performed on the Bio-Rad CFX384 Touch Real-Time PCR Detection System in a 12.5-μl total reaction volume in duplicate. *C*_T_ values were normalized to the calibrator gene glyceraldehyde-3-phosphate dehydrogenase (GAPDH) (Thermo Fisher Scientific catalog no. 4333764 T) and final *C*_T_ value inverted (40 − Δ*t*). Values represent the average of three SARS-CoV-2 genes (N protein, ORF1a, and S protein).

### Measurement of plasma proteins using Olink platform

Proteins in plasma samples (*n* = 11) from a subset of patients with COVID-19 (*n* = 6) and healthy volunteers (*n* = 10) were quantitated using a proximity extension assay (Olink Proteomics, Uppsala, Sweden), which allows for the simultaneous analysis of 92 protein biomarkers on each panel. Sampling time points ranged from between days 3 and 17 of symptom onset (table S2). For each of the inflammation and immune response panels, 1 μl of plasma was incubated at 4°C overnight and allowed to bind with oligonucleotide-labeled antibody pairs to form specific DNA duplexes. The resulting template was then extended and preamplified, and the individual protein markers were measured using high-throughput microfluidic real-time PCR according the manufacturer’s instruction. The resulting *C*_T_ values were normalized against an extension control and an interplate control and adjusted with a correction factor according to the manufacturer’s instructions to calculate a normalized protein expression (NPX) value in log_2_ scale. Statistical analysis included standard *t* test using Benjamini-Hochberg procedure to correct for the false-positive rate in multiple comparisons.

### Measurement of plasma proteins using Luminex platform

Proteins in plasma samples (*n* = 11) from patients with COVID-19 (*n* = 6) and healthy volunteers (*n* = 10) were quantitated using the Luminex Human Pre-mixed Multi-Analyte Kit (catalog no. LXSAHM) according to the manufacturer’s instructions. Samples were run on a MAGPIX instrument. Analytes included ADAMTS13, tissue factor, uPA, and PAI-1.

### Microarray gene expression profiling

Gene expression profiling experiments were performed using Agilent Human Whole Genome 44K microarrays. Fluorescent probes were prepared using an Agilent Quick Amp labeling kit according to the manufacturer’s instructions, with the exception of the fragmentation step that was eliminated. RNA samples were isolated from 20 different FFPE lung blocks from 13 fatal COVID-19 cases (table S5). Each RNA sample was labeled and hybridized to individual arrays. Spot quantitation was performed using Agilent’s Feature Extractor software, and data were uploaded into Genedata Analyst 9.0 (Genedata, Basel, Switzerland). Pearson correlation, Student’s *t* test, and principal components analyses were performed using Genedata Analyst 9.0. The Benjamini-Hochberg procedure was used to correct for the false-positive rate in multiple comparisons.

### Statistical analysis

Normalization of microarray data was performed in Genedata Analyst using central tendency, followed by relative normalization using pooled normal (*n* = 3) as a reference. Pearson correlation, Student’s *t* test, and principal component analyses were performed using Genedata Analyst 9.0. The Benjamini-Hochberg procedure was used to correct for the false-positive rate in multiple comparisons. Ingenuity pathway analysis was used for gene ontology enrichment using Bonferroni correction. Additional statistical analysis was performed using R statistical software (4.1.0/18 May 2021; Foundation for Statistical Computing, Vienna, Austria).

## Supplementary Material

suppl figures

## Figures and Tables

**Table 1.** Summary of COVID-19 autopsy cases. N, no; Y, yes; Pos, positive; Neg, negative; NA, not available.

Case no.	State	Age (years)	Sex	Race	Time of SOTD (days)	Duration of hospitalization, (days)	Mechanical ventilation	SARS-CoV-2 viral PCR*	Plasma Ab titers^†^
1	NY	64	F	White	3	0	N	42.4	NA
2	NY	60	M	White	7	0	N	44.5	NA
3	CA	101	F	White	7	5	N	33.0	NA
4	CA	85	M	White	7	5	N	32.5	Pos
5	CA	87	F	White, Hispanic	7	7	N	41.3	NA
6	CA	70	M	White, Hispanic	8	6	Y	32.5	NA
7	NY	50	F	Hispanic	9	2	Y	36.8	NA
8	CA	73	M	White	11	9	Y	28.2	Pos
9	NY	44	M	Black	13	6	Y	33.3	NA
10	CA	86	F	White	14	12	N	34.5	Neg
11	NY	64	F	Hispanic	16	9	Y	35.8	NA
12	NY	65	M	White	17	3	Y	34.6	NA
13	CA	81	M	White, Hispanic	19	17	Y	32.3	Pos
14	VT	39	M	Hispanic	20	14	Y	29.5	NA
15	NY	55	F	Hispanic	25	11	Y	29.4	NA
16	CA	64	F	White	40	35	Y	18.6	Pos
17	CA	60	M	White, Hispanic	40	34	Y	28.9	Pos
18	CA	70	M	White, Hispanic	47	43	Y	27.7	NA

* Cycle threshold (*C*_T_) values were normalized to the calibrator gene GAPDH, and the final *C*_T_ values were inverted (40 − Δ*t*). Values represent the average of three SARS-CoV-2 genes (N protein, ORF1a, and S protein).

† See table S2.

## Data Availability

All data associated with this study are present in the paper or the Supplementary Materials. The complete MIAME-compliant microarray dataset has been deposited in NCBI’s Gene Expression Omnibus (GEO) (www.ncbi.nlm.nih.gov/geo) and is accessible through GEO Series accession number GSE180226. This work is licensed under a Creative Commons Attribution 4.0 International (CC BY 4.0) license, which permits unrestricted use, distribution, and reproduction in any medium, provided that the original work is properly cited. To view a copy of this license, visit http://creativecommons.org/licenses/by/4.0. This license does not apply to figures/photos/artwork or other content included in the article that is credited to a third party; obtain authorization from the rights holder before using this material.
