# Supplementary material for: Lung epithelial and endothelial damage, loss of tissue repair, inhibition of fibrinolysis, and cellular senescence in fatal COVID-19
Source: Sci Transl Med. Author manuscript; Available in PMC 2024 Apr 8. (PMC11000440; doi:10.1126/scitranslmed.abj7790)
Supplement: suppl figures [file NIHMS1982184-supplement-suppl_figures.pdf]

Science Translational Medicine
AAAS

Supplementary Materials for

Lung epithelial and endothelial damage, loss of tissue repair, inhibition of fibrinolysis, and cellular senescence in fatal COVID-19

Felice D'Agnillo *et al.*

Corresponding author: Jeffery K. Taubenberger, taubenbergerj@niaid.nih.gov

Sci. Transl. Med. **13**, eabj7790 (2021)
DOI: 10.1126/scitranslmed.abj7790

The PDF file includes:

Materials and Methods
Figs. S1 to S23
Tables S1 to S12
References (*95–102*)

Other Supplementary Material for this manuscript includes the following:

Data file S1

MATERIALS AND METHODS

Immunohistochemistry and immunofluorescence protocols

FFPE lung sections were dewaxed, rehydrated, and heat-treated in a microwave oven for 15 minutes in 10 mM sodium citrate buffer (pH 6.0) or 10 mM Tris/1 mM EDTA buffer (pH 9.0). After cooling for 30 min at room temperature (RT), slides were washed in phosphate-buffered saline with 0.05% Tween-20 (PBST) and incubated with Bloxall (Vector Laboratories, Burlingame, CA) for 15 min at RT to inhibit endogenous peroxidases. After a brief wash, sections were blocked in 2.5% horse serum for 30 min at RT and incubated overnight at 4°C with primary antibodies in PBST containing 2.5% horse serum (table S12). For negative controls, sections were incubated without the primary antibody or mouse and rabbit isotype antibody controls. Signal was developed using polymeric peroxidase-conjugated secondary antibodies (Vector ImmPRESS Kit) and ImmPACT DAB (Vector Laboratories). Slides were washed, dehydrated in graded ethanol and SafeClear II (xylene substitute), and mounted using Permount (ThermoFisher, Waltham, MA). Images were captured using an Olympus IX71 inverted microscope fitted with Plan Apochromat 40x and 60x objectives and an Olympus DP80 digital camera and processed using cellSens imaging software. For immunofluorescence analyses, heat-retrieved sections were blocked in PBST with 2.5% bovine serum albumin (BSA) for 30 min at RT followed by overnight incubation at 4°C with primary antibodies in 1% BSA. Sections were rinsed and incubated with Alexa Fluor 488 and Alexa Fluor 647-conjugated secondary antibodies for 1 hour at RT (ThermoFisher, Waltham, MA). Nuclei were counterstained with Hoechst 33342. For double labeling experiments, primary antibodies were mixed and incubated overnight at 4°C. Sections stained with secondary antibodies alone showed no specific staining. Images were captured using an Axio Observer Z1 inverted microscope (Carl Zeiss, Thornwood, NY) equipped with an Axiocam 506 monochrome

camera, an ApoTome.2 optical sectioning system, and a Plan-Apochromat 63x/1.4NA oil immersion with WD=0.19 and Plan-Apochromat 20x/0.8 objective lens. Digital image post-processing and analysis were performed using the ZEN 2 ver. 2.0 imaging software. Images were constructed from Z-stack slices collected at 0.6 µm intervals (6 µm thickness in total) and visualized as maximum intensity projections in orthogonal mode. Whole slide brightfield and fluorescence imaging was also performed using a Hamamatsu NanoZoomer 2.0-RS whole-slide digital scanner with a fluorescence module #L11600 and NDP.view2 software was used for processing (Hamamatsu Photonics, Japan).

DAD histopathology image analysis

H&E-stained lung sections from COVID-19 patients were scored for DAD histopathology by two qualified and blinded investigators (FD, JKT). Histological criteria were defined for DAD stages including acute (hyaline membranes, interstitial or alveolar edema, epithelial sloughing, alveolar/interstitial inflammation); organizing (epithelial hyperplasia, loose organizing fibrosis); and fibrotic (dense interstitial fibrosis, epithelial metaplasia/bronchiolization). Histological grading of severity or prevalence for these histological criteria was scored on a scale of 0 – 3 (0 – absent or minimal, 1 – mild, 2 – moderate, 3 – severe). High resolution whole-slide digital images of each lung section were divided into four quadrants and each quadrant was assigned a score for acute, organizing, and fibrotic DAD. The individual quadrant scores (n = 8 scores per DAD stage) were averaged and a final score for each DAD stage was derived for each lung section.

Immunofluorescence image analyses

Semiquantitative and blinded evaluation of pulmonary neutrophils was performed on sections stained by MPO immunofluorescence. A total of 20-25 randomly selected fields from high resolution whole-slide digitized images of MPO-stained lung sections were examined at 400x magnification. For each field, the number of MPO-positive cells were counted, averaged, and a final count per high power field (HPF) was derived for each lung section. Nucleated MPO-stained cells were counted as positive regardless of staining intensity or morphology, and stained cells in large or medium-sized vessels were excluded.

Semiquantitative analysis of Pro-SPC immunofluorescence was performed in lung sections from non-COVID-19 patients (n = 5) and a subset of COVID-19 cases with predominant acute DAD histopathology (n = 4). Pro-SPC stained sections were examined at 400x magnification and a total of 15 high resolution fields were captured. For each field, nucleated cells with detectable Pro-SPC reactivity localized to alveolar septa were counted, averaged, and a final count per HPF was derived for each lung section. Cases with predominant organizing and fibrotic DAD were not evaluated as alveolar septa were not easily distinguishable and aberrant Pro-SPC expression in hyperplastic and metaplastic epithelial lesions precluded reliable analysis.

Semiquantitative analysis of CD163 immunofluorescence was performed in lung sections from non-COVID-19 patients (n = 3) and COVID-19 cases with acute (n = 5) and fibrotic DAD (n = 4) as determined by histological grading. A total of 15 randomly selected fields were acquired at 200x magnification using the Zeiss ApoTome.2 system, and 16-bit image files were processed using the Fiji/ImageJ software version 1.53c (NIH, Bethesda, MD; http://imagej.nih.gov/ij). Threshold limits were normalized across all analyses, and fluorescence integrated density measurements were

recorded and averaged for each case. Final values were reported as the means ± SD for each analysis group.

Semiquantitative evaluation of VWF immunofluorescence was performed on lung sections from non-COVID-19 patients (n = 5) and COVID-19 cases (n = 17). High resolution whole-slide digital images of each lung section were divided into four quadrants. Each quadrant was assigned a score for VWF staining based on the following scale (0 – 3): 0 – absent or minimal staining, 1 – detectable vessel and capillary staining, 2 – increased vessel lumen-associated VWF expression with detectable extravascular VWF staining affecting < 50% of the field, 3 – increased vessel lumen-associated VWF expression with detectable extravascular VWF staining affecting > 50% of the field. The individual quadrant scores were averaged to generate a final VWF score for each lung section.

Protein structure

The crystal structures of SARS-CoV-2 RBD in complex with neutralizing antibody H014 (7CAH) and CC12.1 (6XC2) were downloaded from the Research Collaboratory for Structural Bioinformatics (RCSB) Protein Data Bank (PDB) (http://www.rcsb.org/pdb/home/home.do) (*95*), and amino acid changes in RBD were made and viewed with Swiss-PdbViewer (*96*).

Library construction and sequencing. Three normal lung total RNA samples were purchased from BioChain Institute, Inc (Newark, CA), Thermo Fisher Scientific Inc. (Waltham, MA), and Takara Bio Inc. (Kusatsu, Shiga, Japan), respectively. First, isolated total RNA from FFPE samples and purchased normal lung total RNA were performed rRNA depletion by using NEBNext®

rRNA Depletion Kit (New England Biolabs, Ipswich, MA). Seven µl of rRNA depleted RNA was used to construct total RNA library and was performed according to TruSeq RNA Library Prep Kit v2 (Illumina, San Diego, CA, USA) eliminating the poly-A selection and RNA fragmentation steps (library construction from normal lung RNA only eliminated play-A selection step). For SARS-CoV-2 enriched library, isolated total RNA from FFPE sample was amplified using the Ovation RNA-Seq system V2 from NuGEN (NuGEN, San Carlos, CA). For each sample, 5µl of total RNA was used as input. The amplified total cDNAs were analyzed by an Agilent 2100 Bioanalyzer using the Agilent High Sensitivity DNA Kit (Agilent Technologies, Santa Clara, CA) and sheared to 150bp on the Covaris S2 machine (Covaris, Woburn, MA). The Agilent 2100 Bioanalyzer was then used to analyze amplified sheared cDNA using the Agilent High Sensitivity DNA Kit (Agilent Technologies, Santa Clara, CA) again. Approximately 300ng of amplified cDNA was used to generate the Illumina sequencing library using the Agilent SureSelect^{XT} Target Enrichment Kit (Agilent, Santa Clara, CA) enriched with SureSelect Community Design Pan Human Coronavirus Panel for Illumina Multiplex Sequencing. Final Illumina sequencing libraries were analyzed with the Agilent 2100 Bioanalyzer using the Agilent High Sensitivity DNA Kit. Libraries were then pooled and sequenced on an Illumina NextSeq sequencer (Illumina, San Diego, CA). In this project, more than 3.4 billion reads were generated. All sequences generated were deposited as a series into the GenBank SRA database.

Data analysis. Reads were mapped to the Bowtie2 (*101*) (version 2.3.4.1, http://bowtie-bio.sourceforge.net/bowtie2/index.shtml) indexed *H. sapiens*, GRCh38 downloaded from the Center for Computational Biology, Johns Hopkins University (http://ccb.jhu.edu/) or Hisat2 (*97*) indexed SARS-CoV-2/human/USA/NY-PV08472/2020 genome (GenBank: MT370889.1) using

Bowtie2 or Hisat2. SAMtools mpileup (version 2.1.0) (*100*) was used to generate mpileup files that were subsequently used to make SNP calls by VarScan2 (*98, 99*). Bacteria classification in all the samples was done by running Kraken2 (*102*) on the reads that failed to be mapped to the human genome. A reported SNP call was the one that satisfied the following criteria at the SNP position: 1) more than 100 reads at that position, 2) minimum base Phred quality score as 25, 3) the different bases were more than 10% of the aligned reads, 4) pass VarScan2 Strand Filter, 5) VarScan2 SNP call p-value less than 0.05. Consensus nucleotide sequence was generated by VarScan2 with default setting. The amino acid replacements in SARS-CoV-2 genomes were downloaded on Nov 19, 2020 from CoV-GLUE site that tracks the mutations from sequenced SARS-CoV-2 genomes (http://cov-glue.cvr.gla.ac.uk/#/home), which was compared to the called SNPs in our study.

Strain selection to place autopsy samples into local/global context

Nextstrain[1] SARS-CoV-2 North America, which is a sample of all strains isolated in North America, aimed at covering the genetic diversity of SARS-CoV-2 circulating up to November 2020 (https://nextstrain.org/ncov/north-america) was used for local context. Sequences of all New York State (NY) and Californian (CA) isolates were downloaded from GISAID[2]. Ten isolates each from NY and CA were randomly selected. For the global context, the Nextstrain SARS-CoV-2 Global build (https://nextstrain.org/ncov/global) was used, which is a sample of all strains isolated up to November 2020. Next, we down sampled the dataset to 25 strains using Treemmer[2] and subsequently downloaded the sequences from GISAID[2].

Alignment, phylogenetic inference and visualization

Autopsy samples that passed the Nextclade (https://clades.nextstrain.org/ v.0.8.1) quality filtering thresholds were included. The autopsy sequences were aligned and the local/global diversity set to the reference genome (NC_045512.2) with mafft (v.7.467). The phylogeny was inferred using IQtree (v.1.6.12) with the best-fitting substitution model (GTR+F+I+G4) and 1000 rapid boostrap replicates. The phylogeny was plotted using the package ggtree (v.2.0.4)[3] for R (v. 3.6.2).

SUPPLEMENTARY FIGURES

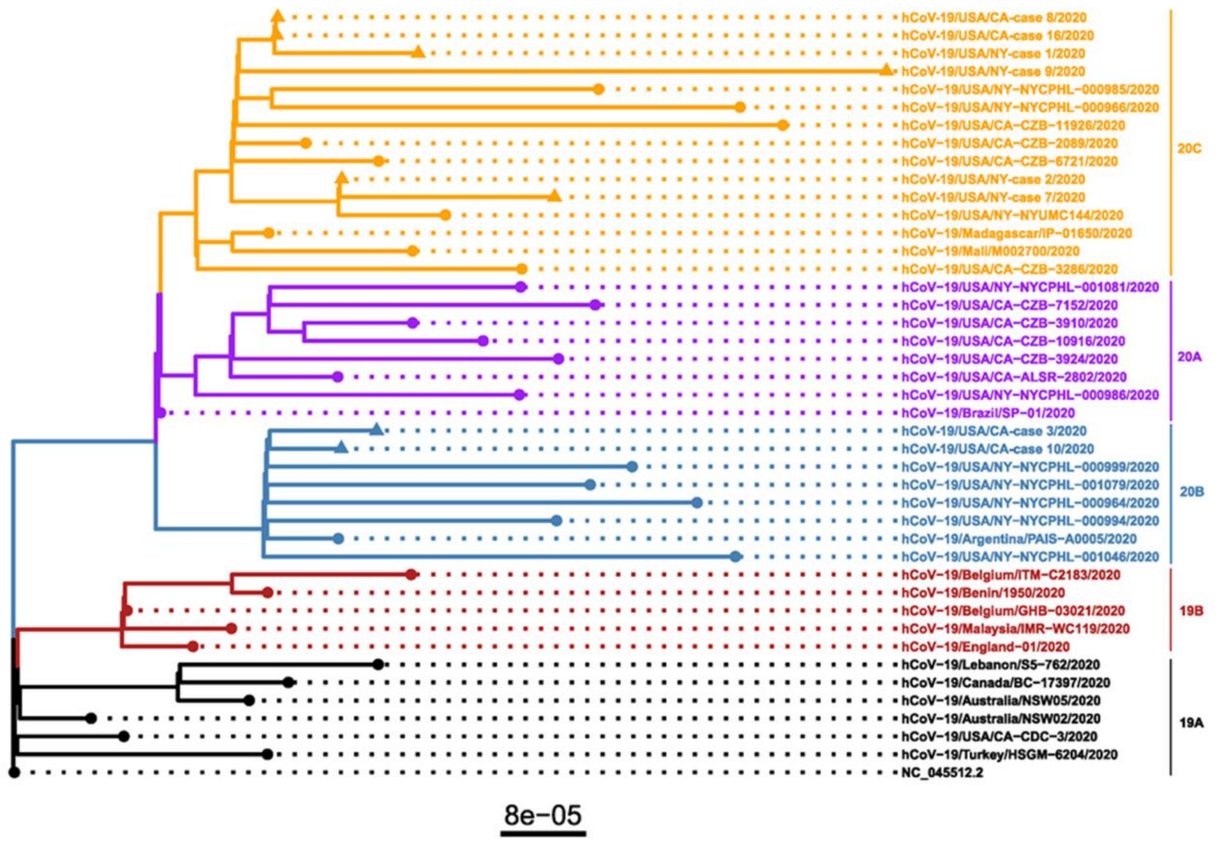

Fig. S1. SARS-CoV-2 phylogeny. Whole genome maximum likelihood phylogeny based on 42 SARS-CoV-2 isolates. Triangular tip labels depict autopsy samples. The autopsy samples were put into a local, as well as global context by randomly selecting 10 isolates each from New York State/California and 15 "global" isolates (see Materials and Methods). Scale bar depicts numbers of substitutions/per site. Lineage labels correspond to Nextclade (v.0.11.2) clades.

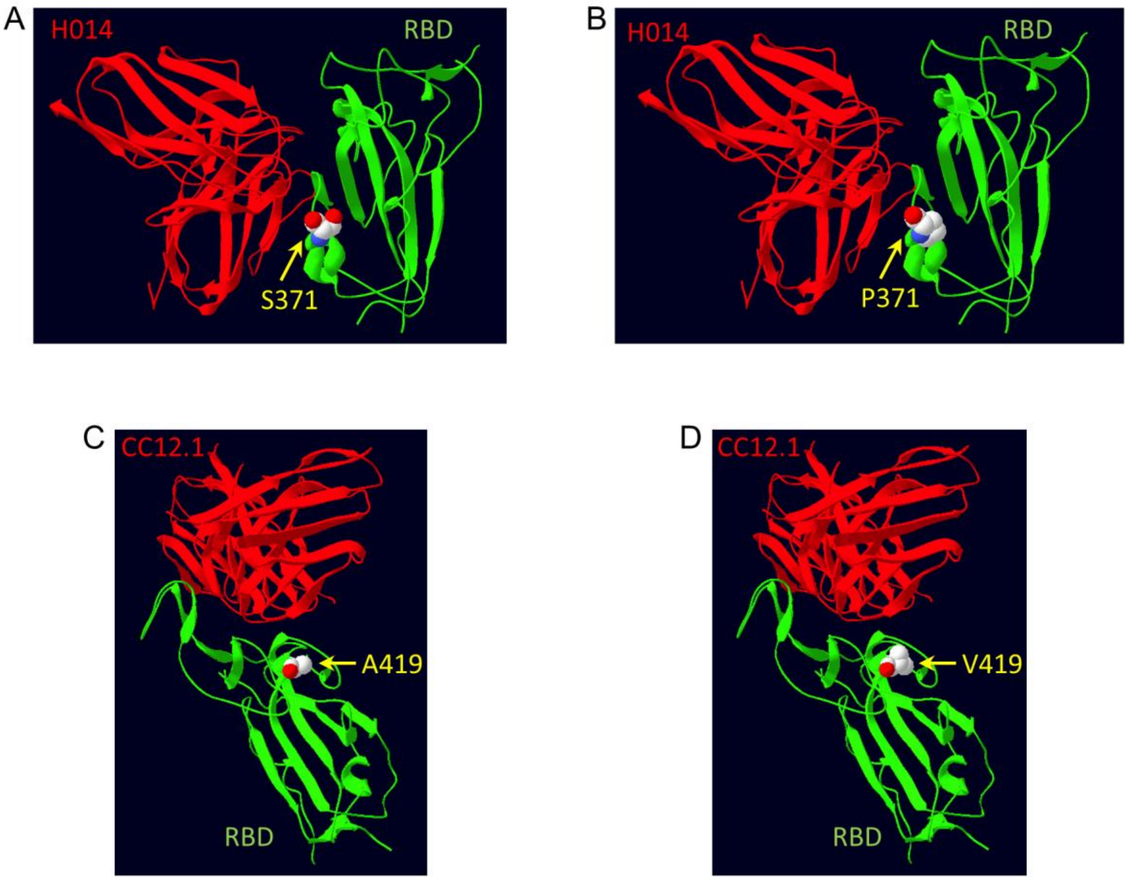

Fig. S2. Structures of RBD SNP mutations with neutralizing antibodies. The structures of 2 RBD SNP mutations related to neutralizing antibody H014 and CC12.1 (**A**) RBD S371 related to H014 (**B**) RBD P371 related to H014 (**C**) RBD A419 related to CC12.1 (**D**) RBD V419 related to CC12.1

Fig. S3

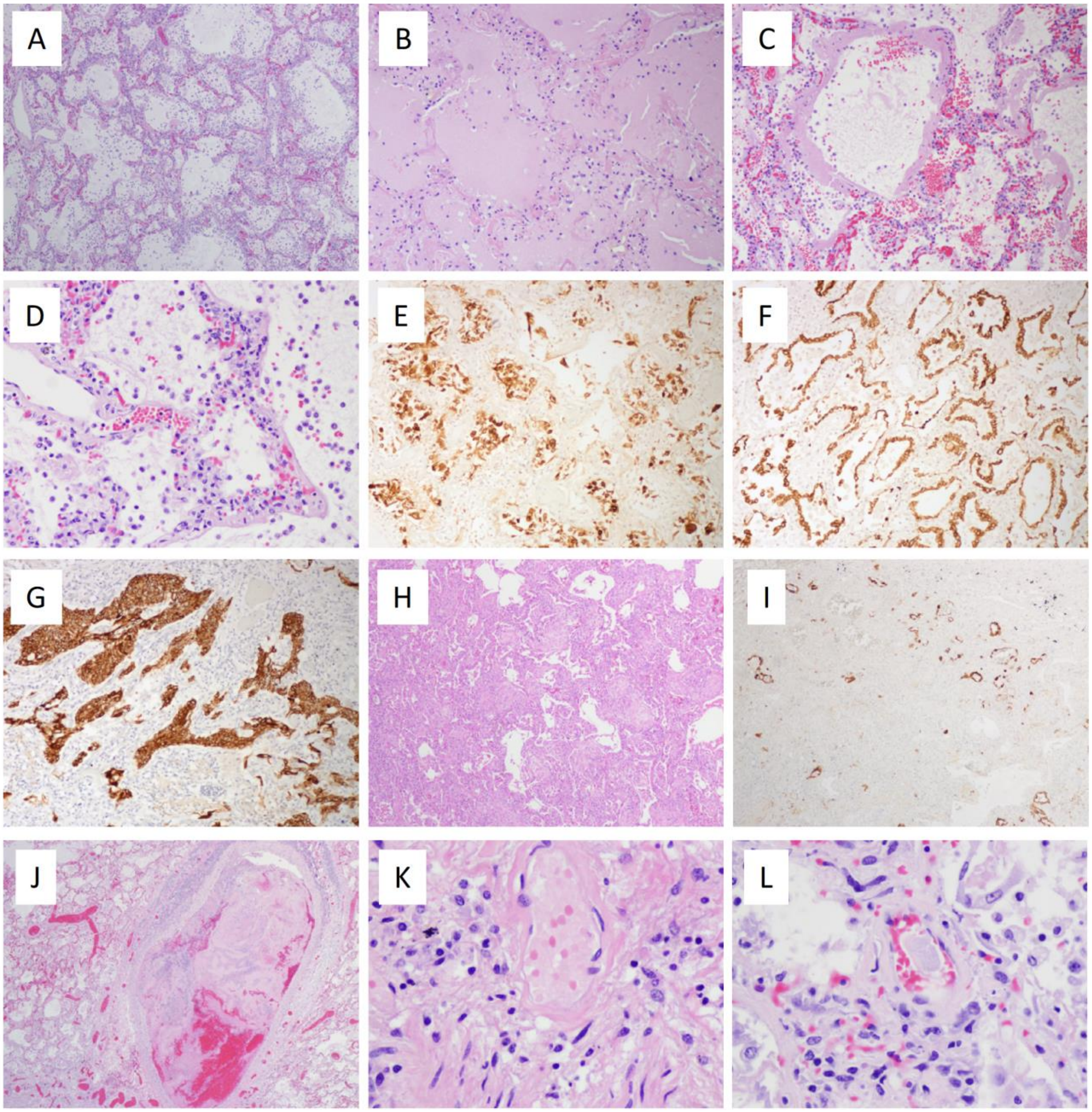

Fig. S3. Overview of SARS-CoV-2 pulmonary pathology. Examples of histopathological features seen in short (**A-E**), intermediate (**F** and **G**), and long SOTD cases (**H** and **I**), and representative vascular thrombi (**J-L**). (A) Acute diffuse alveolar damage (DAD), original magnification 100x. (B) Acute DAD with pulmonary edema and interstitial inflammation, original magnification 200x. (C) Acute DAD with hyaline membranes, original magnification 200x. (D) Acute DAD with luminal and interstitial neutrophilic infiltrates, original magnification 400x. (E) Abundant detached respiratory epithelial cells in alveolar lumina (cytokeratin stain), original magnification 100x. (F) Alveolar type II cell hyperplasia (cytokeratin stain), original magnification 100x. (G) Squamous metaplasia of the epithelium of bronchiole (cytokeratin stain), original magnification 100x. (H) Interstitial pulmonary fibrosis, original magnification 40x. (I) Loss of alveoli in fibrotic lung (cytokeratin stain), original magnification 40x. (J) Acute thrombus in a medium sized vessel, original magnification 40x. (K and L) Microvascular thrombi, original magnification 400x.

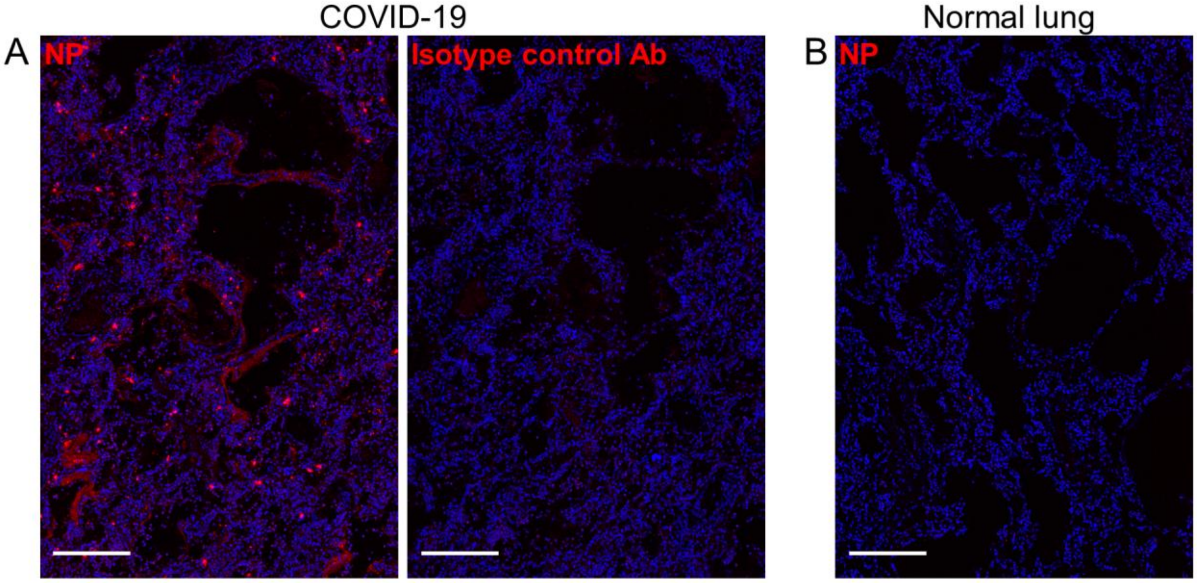

Fig. S4. Antibody staining controls for SARS-CoV-2 nucleocapsid protein. Serial sections from a COVID-19 lung (case 1) immunostained with (**A**) SARS-CoV-2 nucleocapsid protein (NP) antibody or a mouse isotype control antibody. (**B**) Negative NP immunoreactivity observed in a normal lung section from a non-COVID-19 patient. Nuclei were counterstained with Hoechst 33342 (blue). Scale bars: 250 µm.

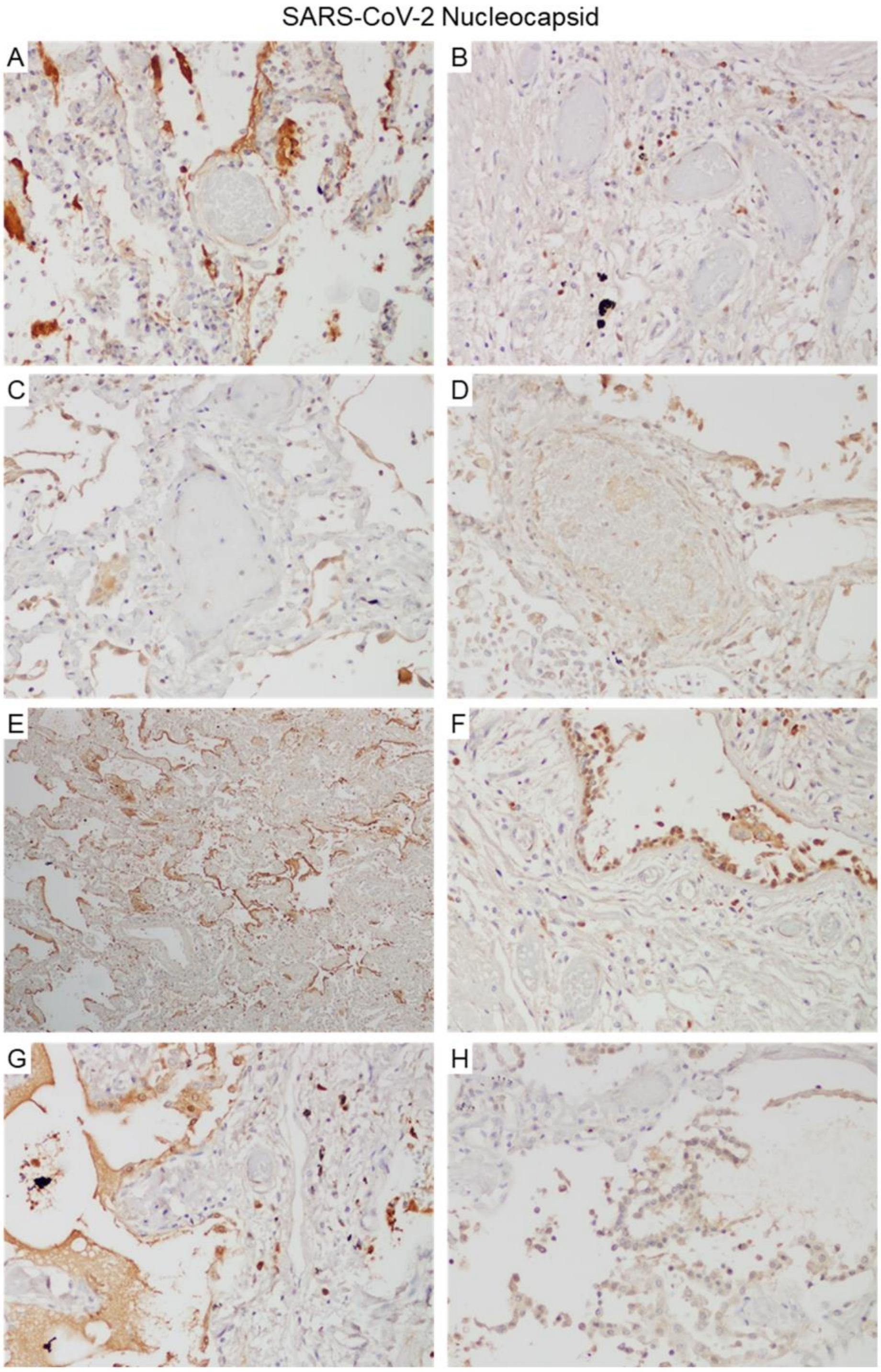

Fig. S5. Pulmonary SARS-CoV-2 immunohistochemistry. Representative staining of SARS-CoV-2 nucleocapsid protein (NP) in COVID-19 lung sections (cases 1 and 2). Weak but detectable NP staining in blood vessel lumens and endothelial cells (**A-D**) compared to marked NP deposition in hyaline membranes and sloughed alveolar/bronchiolar epithelium (**E-H**). Original magnification 200x.

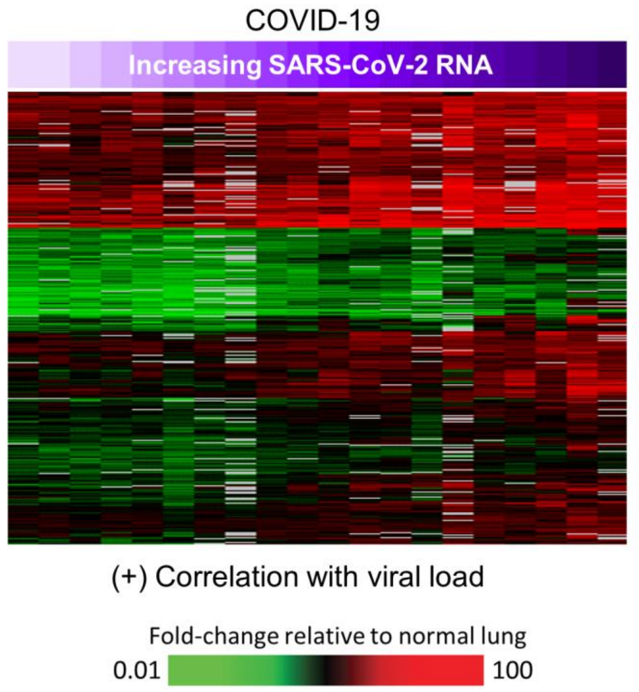

Fig. S6. Pulmonary gene expression correlated positively with lung viral load. Heatmap showing expression profiles of transcripts with expression levels that positively (n = 368) correlated with SARS-CoV-2 viral load in lung tissue (≥0.6). Each column represents gene expression data from a microarray experiment comparing patient lung tissue relative to pooled RNA isolated from normal lung tissue (n = 3). Genes shown in red were increased, genes shown in green were decreased, and genes in black indicate no change in expression in COVID-19 relative to normal lung tissue. Levels of SARS-CoV-2 RNA are indicated by a purple gradient bar with values ranging from 0 to 44.9. Viral C_T values were normalized to the calibrator gene GAPDH and final C_T value inverted (40-Δt) such that a higher value represents a higher viral load (see methods).

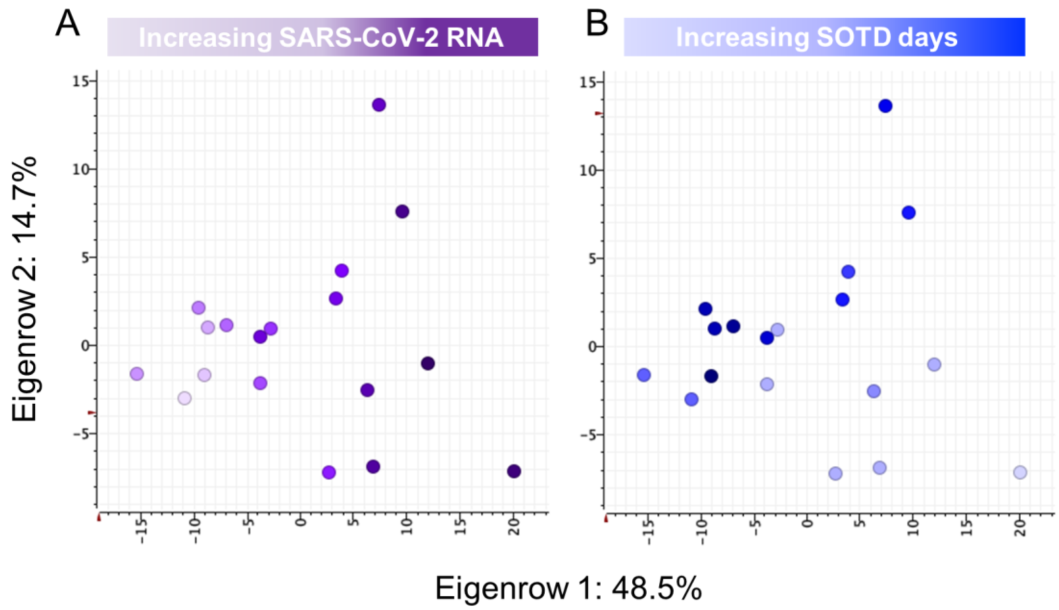

Fig. S7. Pulmonary viral load and associated gene expression is inversely correlated with duration of clinical illness prior to death. (**A**) Principal-component analysis (PCA) of viral load associated gene expression identified by Pearson correlation (>0.6). Each participant is shown as a separate circle with viral load in lung tissue indicated using a purple gradient with values ranging from 0 to 44.9. Viral C_T values were normalized to the calibrator gene GAPDH and final C_T value inverted (40-Δt) such that a higher value represents a higher viral load (see methods). (**B**) PCA of viral load associated gene expression showing days of symptom onset to death (SOTD) for each participant using a blue gradient.

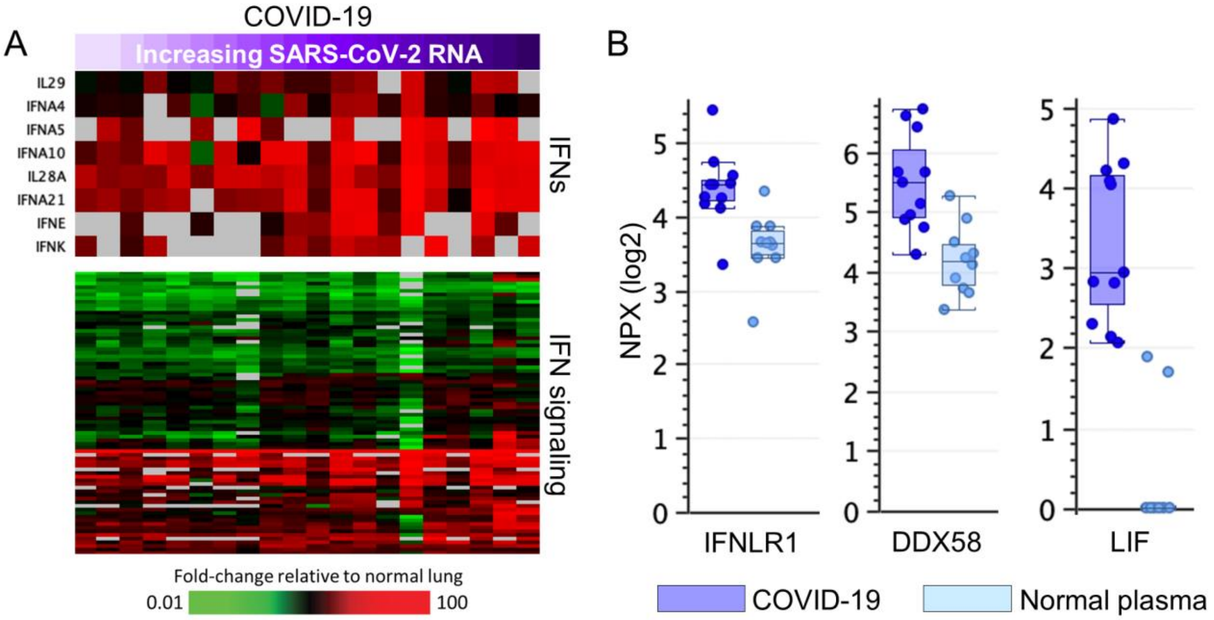

Fig. S8. Induction of antiviral response in autopsy lung tissue from COVID-19 patients. (**A**) Heatmap showing expression levels of genes encoding interferons (IFN) and IFN-related signaling proteins. Each heatmap column represents gene expression data from a microarray experiment comparing patient lung tissue relative to pooled RNA isolated from normal lung tissue (n = 3). Genes shown in red were increased, genes shown in green were decreased, and genes in black indicate no change in expression in COVID-19 relative to normal lung tissue. Levels of SARS-CoV-2 RNA in lung tissue are indicated by a purple gradient bar with values ranging from 0 to 44.9. Viral C_T values were normalized to the calibrator gene GAPDH and final C_T value inverted (40-Δt) such that a higher value represents a higher viral load (see methods). (**B**) Levels of antiviral-related proteins in plasma from COVID-19 (n = 6) and healthy volunteers (n = 10) measured by Olink platform. Values were statistically significant (p value <0.05) between the groups using standard t test. Scale of NPX value is log2.

A

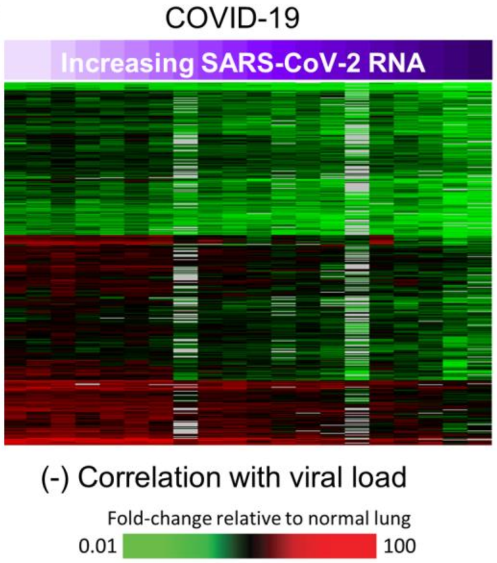

B

Function	p-value
Organismal Injury and Abnormalities	1.73E-14-1.3E-02
Carbohydrate Metabolism	5.06E-11-1.3E-02
Lipid Metabolism	5.06E-11-1.3E-02
Molecular Transport	5.06E-11-1.3E-02
Small Molecule Biochemistry	5.06E-11-1.3E-02
Respiratory Disease	6.7E-07-1.3E-02
Cell Morphology	7.68E-07-1.3E-02
Embryonic Development	7.68E-07-1.3E-02
Organismal Development	7.68E-07-1.3E-02
Respiratory System Development and Function	7.68E-07-1.3E-02
Tissue Development	7.68E-07-1.3E-02
Antimicrobial Response	8.68E-06-8.68E-06
Cell-To-Cell Signaling and Interaction	2.15E-05-1.3E-02
Inflammatory Disease	2.15E-05-1.3E-02

Fig. S9. Pulmonary gene expression correlated negatively with lung viral load. (**A**) Heatmap showing expression profiles of transcripts with expression levels that negatively (n = 450) correlated with SARS-CoV-2 viral load in lung tissue (≥0.6). Each column represents gene expression data from a microarray experiment comparing patient lung tissue relative to pooled RNA isolated from normal lung tissue (n = 3). Genes shown in red were increased, genes shown in green were decreased, and genes in black indicate no change in expression in COVID-19 relative to normal lung tissue. Levels of SARS-CoV-2 RNA are indicated by a purple gradient bar with values ranging from 0 to 44.9. Viral C_T values were normalized to the calibrator gene GAPDH and final C_T value inverted (40-Δt) such that a higher value represents a higher viral load (see methods). (**B**) Gene ontology analysis showing enriched biological functions.

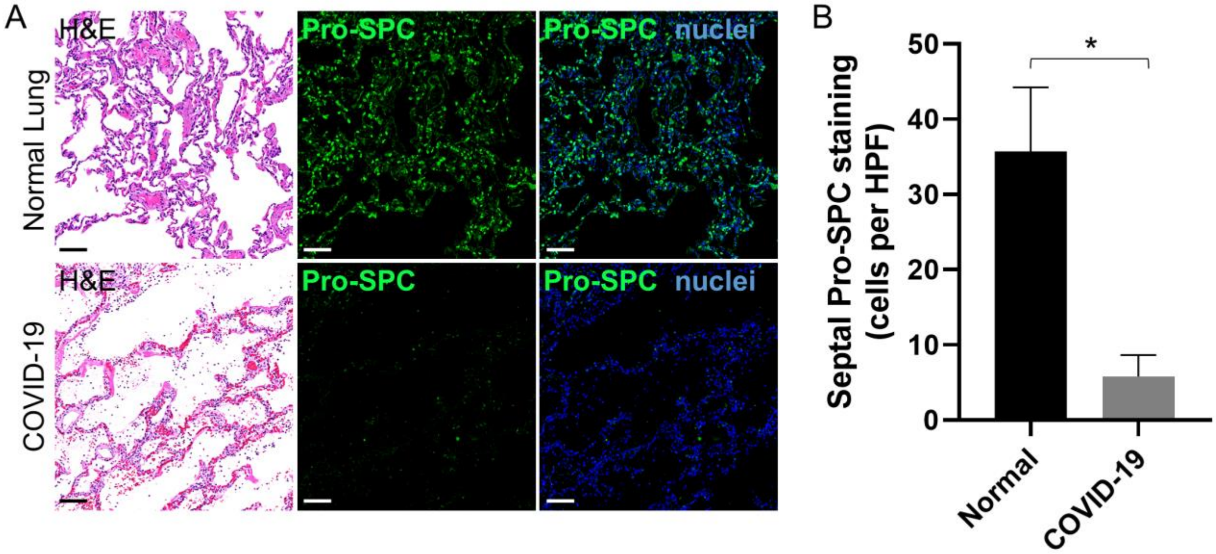

Fig. S10. Reduced Pro-SPC expression in COVID-19 lungs. (**A**) Representative H&E and immunofluorescence staining of prosurfactant protein C (Pro-SPC) in normal and COVID-19 lung (case 1). Normal lungs show distinct Pro-SPC-expressing AT2 cells populating the alveolar septa compared to marked loss of Pro-SPC staining in the COVID-19 lung. Nuclei were counterstained with Hoechst 33342 (blue). (**B**) Semiquantitative evaluation of Pro-SPC-expressing cells localized to the alveolar septa in normal (n = 5) and COVID-19 lung (n = 4 cases). Pro-SPC expressing cells in alveolar septa were counted, averaged, and a final count per high power field (HPF, n = 15 per section) was derived. Values are shown as the means ± SEM for each group. *$P < 0.05$, Mann-Whitney test. Scale bars: 100 μm.

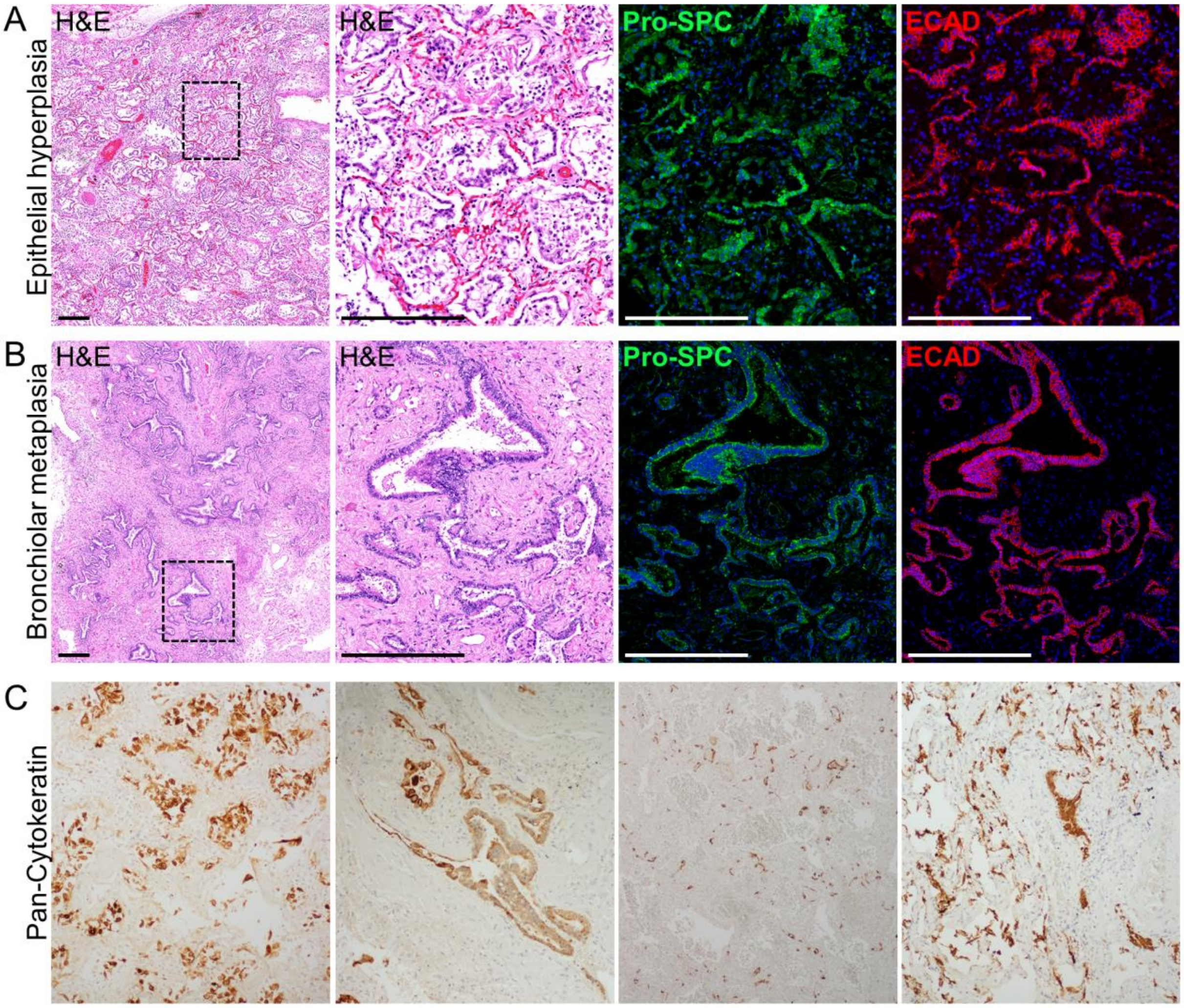

Fig. S11. Organizing DAD with epithelial hyperplasia and bronchiolar metaplasia. Representative images of COVID-19 lung sections with (**A**) epithelial hyperplastic and (**B**) bronchiolar metaplastic lesions serially stained for H&E, Pro-SPC, and E-cadherin (ECAD) (cases 11 and 13). Digitally magnified boxed areas in the low-power H&E panels highlight abnormal colocalized expression of Pro-SPC and ECAD with AT2 hyperplasia (A) and bronchiolization (B). Nuclei were counterstained with Hoechst 33342 (blue). (**C**) Pan-cytokeratin immunohistochemistry shows sloughed epithelial cells in alveolar spaces as well as abnormal expression in hyperplastic/metaplastic airway epithelium. Original magnification 200x (C). Scale bars: 250 μm.

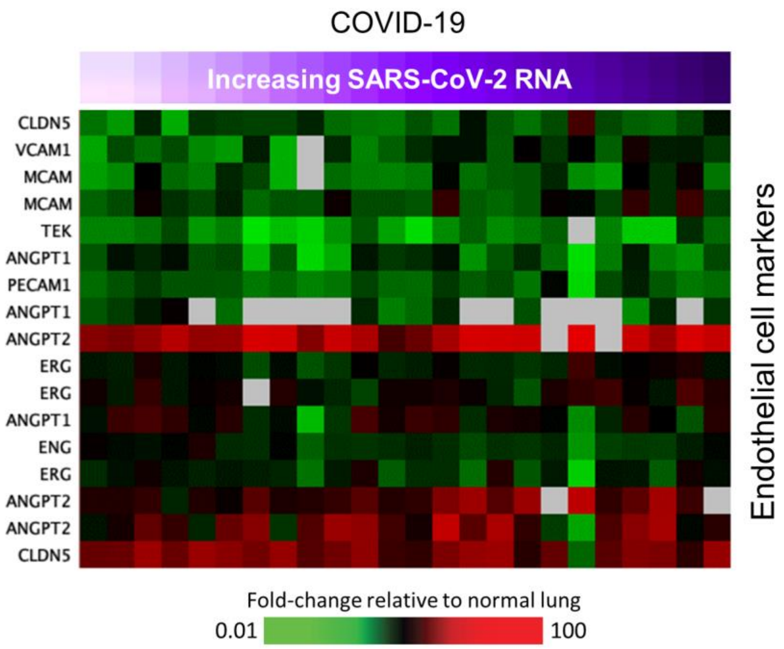

Fig. S12. Loss of endothelial-related gene expression in COVID-19 lungs. Heatmap showing expression profiles of transcripts encoding endothelial cell markers. Each column represents gene expression data from a microarray experiment comparing patient lung tissue relative to pooled RNA isolated from normal lung tissue (n = 3). Genes shown in red were increased, genes shown in green were decreased, and genes in black indicate no change in expression in COVID-19 relative to normal lung tissue. Levels of SARS-CoV-2 RNA are indicated by a purple gradient bar with values ranging from 0 to 44.9. Viral C_T values were normalized to the calibrator gene GAPDH and final C_T value inverted (40-Δt) such that a higher value represents a higher viral load (see methods).

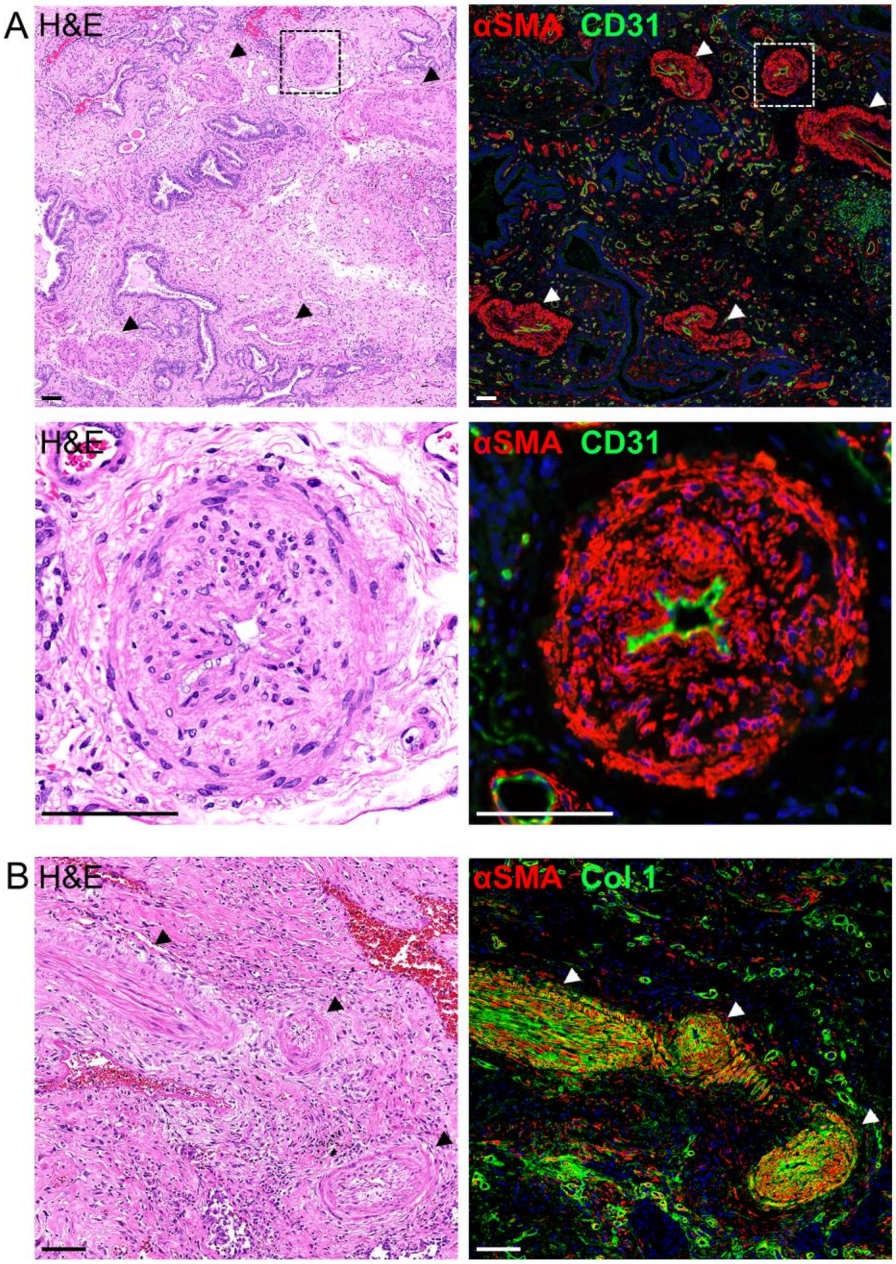

Fig. S13. Pulmonary vascular remodeling in COVID-19 lungs. (**A** and **B**) Representative images of vascular remodeled large blood vessels (arrowheads) in serial COVID-19 lung sections stained for H&E, αSMA, and CD31 or collagen type 1 (Col 1) (cases 17 and 18). High power magnification of the boxed area in (A) depicts a remodeled vessel with significant αSMA-positive tunica media hypertrophy and severely constricted lumen bordered by CD31-positive endothelium. (B) Vascular remodeling with excessive αSMA and Col 1 deposition in vessel walls (arrowheads). Nuclei were counterstained with Hoechst 33342 (blue). Scale bars: 100 μm.

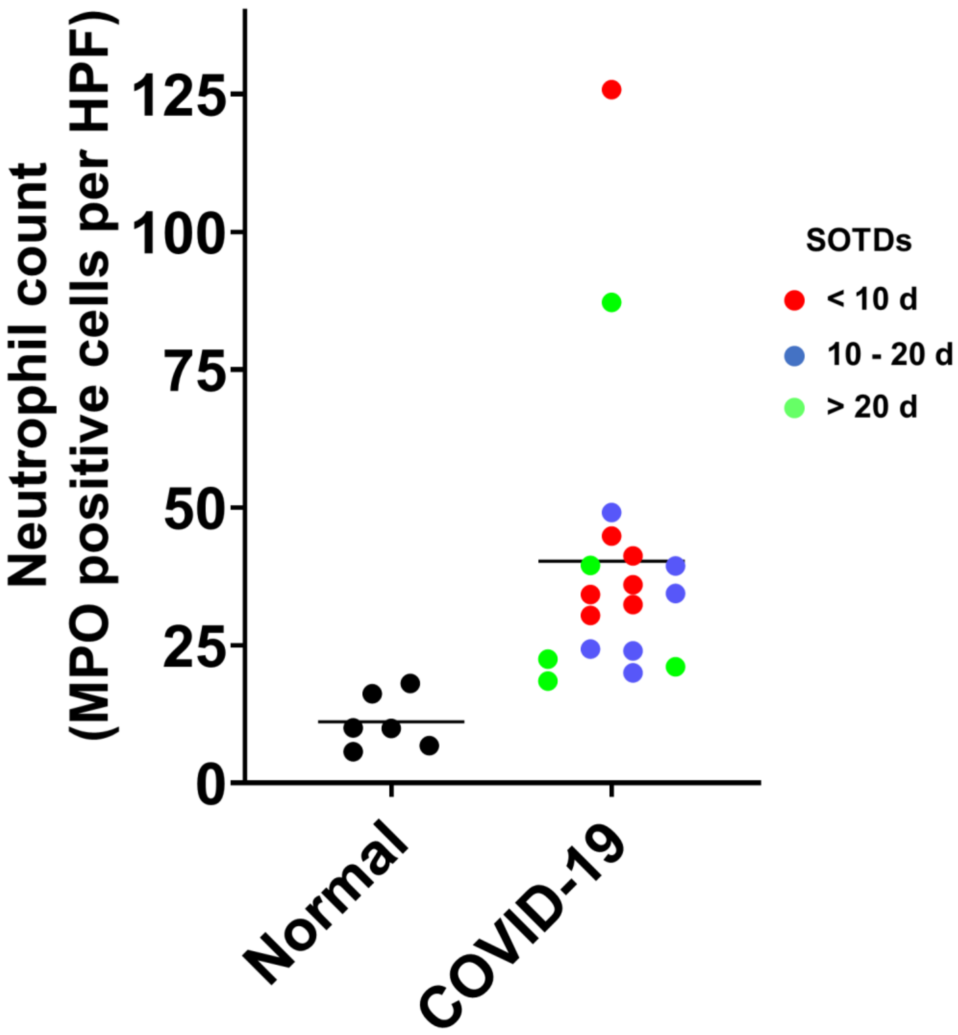

Fig. S14. Semiquantitative analysis of pulmonary neutrophils in COVID-19 patients. Lung sections from non-COVID-19 individuals (n = 6) and COVID-19 cases (n = 18) were stained by MPO immunofluorescence. MPO positive cells were counted, averaged, and a final count per high power field (HPF, n = 20 – 25 per section) was derived (see Supplementary Methods). COVID-19 cases are grouped and color coded based on symptom onset to death times (SOTDs) (red, < 10 days; blue, 10 - 20 days; green, > 20 days). Mean values for each case (circles) and each dataset (horizonal bars) are shown. $P < 0.05$, normal vs. COVID-19; Mann-Whitney test.

Fig. S15

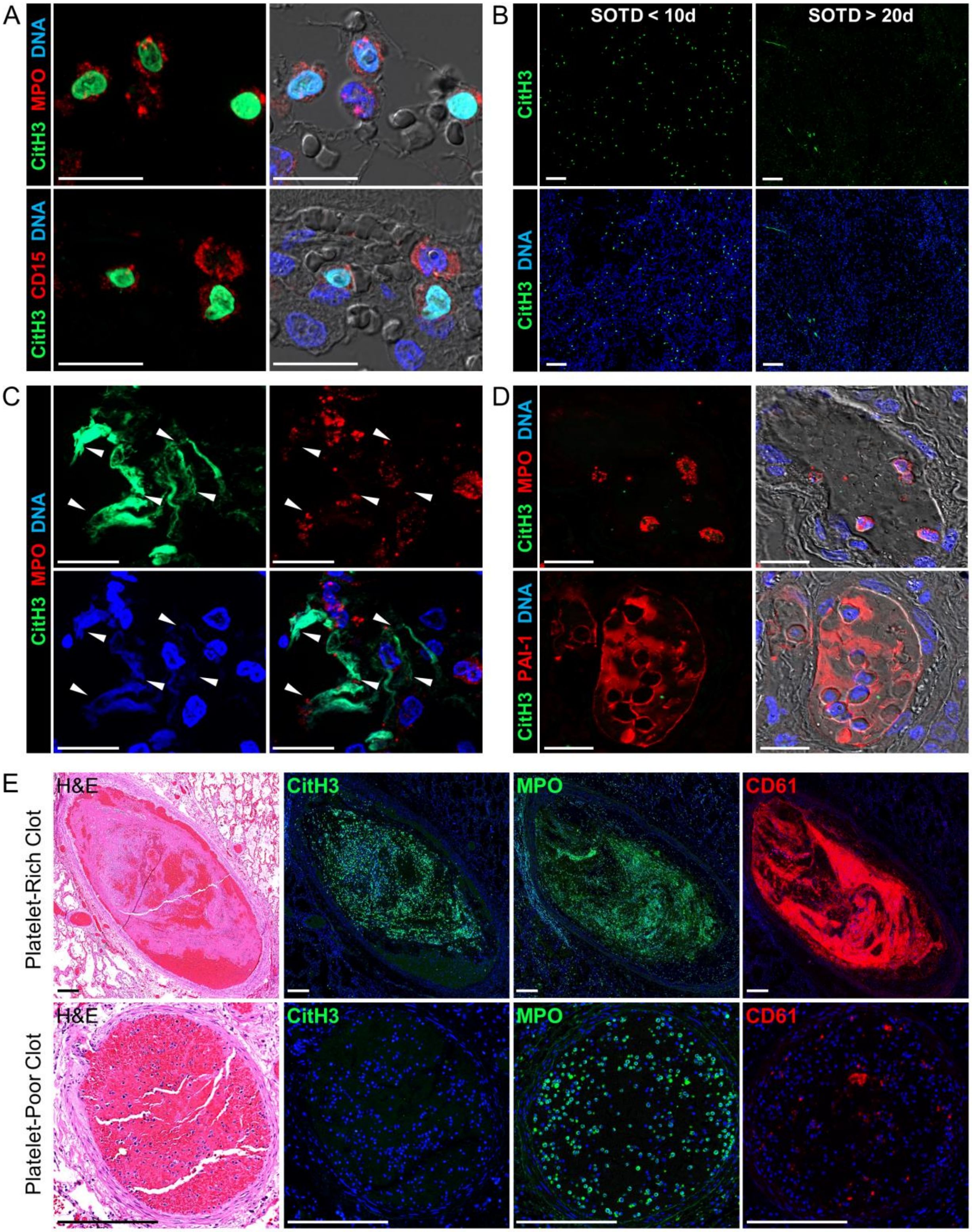

A
CitH3 MPO DNA
CitH3 CD15 DNA
B
SOTD < 10d
SOTD > 20d
CitH3
CitH3 DNA
C
CitH3 MPO DNA
D
CitH3 MPO DNA
CitH3 PAI-1 DNA
E
H&E
CitH3
MPO
CD61
Platelet-Rich Clot
Platelet-Poor Clot

Fig. S15. Immunomarkers of neutrophil NETs in COVID-19 lungs. (**A**) Nuclear expression of citrullinated histone H3 (CitH3) in MPO- and CD15-positive intraalveolar neutrophils in lung sections from a COVID-19 patient with short clinical illness duration (case 2). (**B**) Representative low-power images of CitH3-positive nuclei in lung sections from COVID-19 patients with short (SOTD < 10 d) and long illness duration (SOTD > 20 d) (case 1 and 16). (**C**) Representative intraalveolar NETs stained for CitH3, MPO, and DNA. Arrowheads depict structures with colocalized expression of CitH3, MPO, and cell-free DNA. (**D**) Red blood cell-packed thrombi in COVID-19 lung showing negative staining for CitH3 in MPO and PAI-1 labeled neutrophils. (**E**) Representative platelet-rich and platelet-poor thrombi stained for CitH3, MPO, and platelet marker CD61. Extensive CitH3 staining is embedded within the platelet-rich clot colocalized with extensive MPO and CD61 compared to platelet-poor clots that show negative CitH3 staining even in the presence of a high number of MPO-positive neutrophils. Nuclei and cell-free DNA were counterstained with Hoechst 33342 (blue). Scale bars: 20 µm (A, C, and D); 100 µm (B); 250 µm (E).

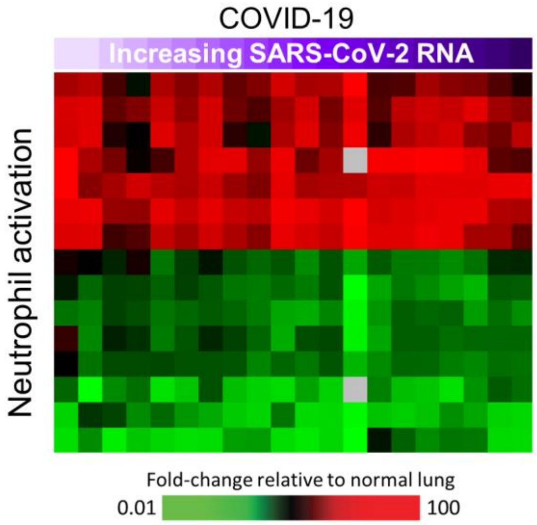

Fig. S16. Pulmonary gene expression associated with neutrophil function. Each column represents gene expression data from a microarray experiment comparing patient lung tissue relative to pooled RNA isolated from normal lung tissue (n = 3). Genes shown in red were increased, genes shown in green were decreased, and genes in black indicate no change in expression in COVID-19 relative to normal lung tissue. Levels of SARS-CoV-2 RNA are indicated by a purple gradient bar with values ranging from 0 to 44.9. Viral C_T values were normalized to the calibrator gene GAPDH and final C_T value inverted (40-Δt) such that a higher value represents a higher viral load (see methods).

Fig. S17

A

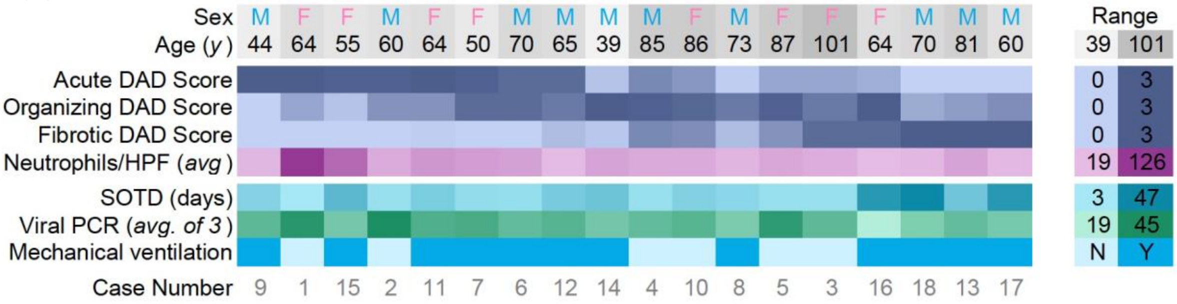

B

Correlation

	**Age (y)**	**Acute DAD Score**	**Organizing DAD Score**	**Fibrotic DAD Score**	**Neutrophils/ HPF**	**Symptom Onset to Death (SOTD)**	**SARS-CoV-2 Viral PCR**
Age (y)	1						
Acute DAD Score	-0.293	1					
Organizing DAD Score	0.32	-0.267	1				
Fibrotic DAD Score	**0.565**	**-0.76**	0.166	1			
AVG Neutrophils/HPF	-0.143	0.458	-0.34	-0.431	1		
Symptom Onset to Death (SOTD)	-0.225	**-0.528**	-0.169	**0.555**	-0.325	1	
SARS-CoV-2 Viral RNA	0.091	**0.538**	-0.109	-0.448	0.395	**-0.722**	1

C

P-value

	**Age (y)**	**Sex**	**Acute DAD Score**	**Organizing DAD Score**	**Fibrotic DAD Score**	**Neutrophils/ HPF**	**Symptom Onset to Death (SOTD)**	**SARS-CoV-2 Viral PCR**
Age (y)								
Sex	0.408							
Acute DAD Score	0.238	0.222						
Organizing DAD Score	0.196	0.655	0.283					
Fibrotic DAD Score	**0.015**	0.845	0.00026	0.510				
AVG Neutrophils/HPF	0.572	0.109	0.056	0.168	0.074			
Symptom Onset to Death (SOTD)	0.369	0.548	**0.024**	0.503	**0.017**	0.188		
SARS-CoV-2 Viral RNA	0.719	0.617	**0.021**	0.667	0.063	0.104	**0.001**	
Mechanical ventilation	**0.029**	0.321	0.475	0.320	0.704	0.460	**0.003**	**0.018**

Fig. S17. Statistical analysis of diffuse alveolar damage (DAD) progression. Relationships between the stage of DAD, neutrophil counts, time of symptom onset to death (SOTD), SARS-CoV-2 viral RNA, and mechanical ventilation status. (**A**) Heatmap showing blind histologic scoring (see Supplementary Methods) of acute, organizing, and fibrotic DAD is shown by purple gradient. Neutrophil counts per high power field (HPF; see Methods) are shown by magenta gradient; SOTD is shown by blue gradient; SARS-CoV-2 RNA concentration, as determined by qPCR (see Methods), is shown by green gradient; and mechanical ventilation status and case number are shown at bottom. For each of these gradient scales, increasing color indicates increasing values, as shown in key on right. (**B**) Statistical analysis. For pairwise comparison of two continuous variables, Pearson's correlation test was used. (**C**) For pairwise comparison contained one categorical variable (e.g. sex or mechanical ventilation), a t-test was used to calculate p-value, without equal variance assumption. For sex vs. mechanical ventilation, a Fisher's exact test was used to test independence.

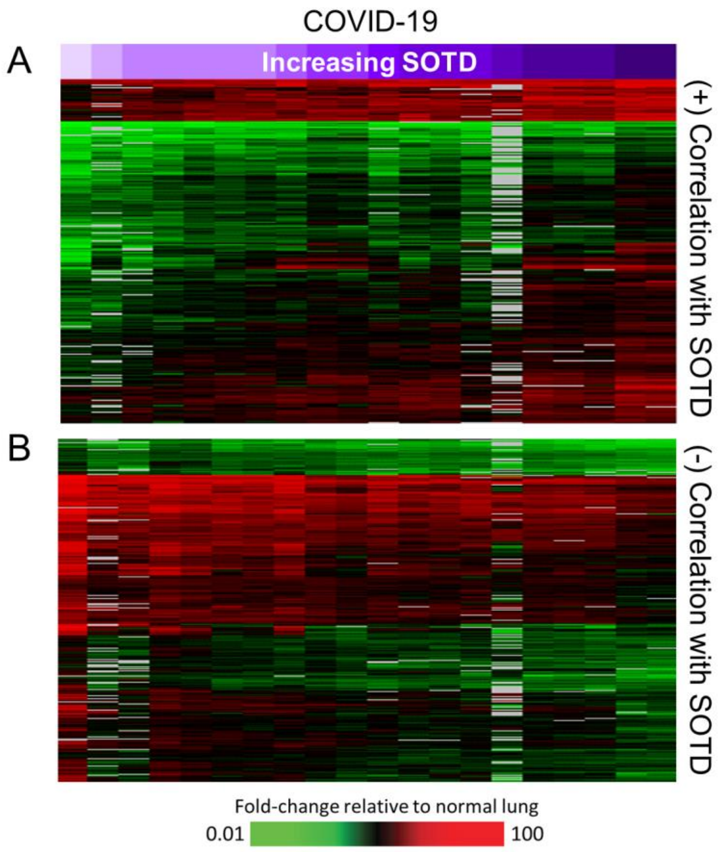

Fig. S18. Pulmonary gene expression correlated with duration of clinical illness. Heatmap showing expression profiles of transcripts with expression levels that (**A**) positively (n = 263) and (**B**) negatively (n = 333) correlated with days of symptom onset to death (SOTD) in lung tissue (≥0.6). Each heatmap column represents gene expression data from a microarray experiment comparing patient lung tissue relative to pooled RNA isolated from normal lung tissue (n = 3). Genes shown in red were increased, genes shown in green were decreased, and genes in black indicate no change in expression in COVID-19 relative to normal lung tissue. Days of SOTD are indicated by a purple gradient bar.

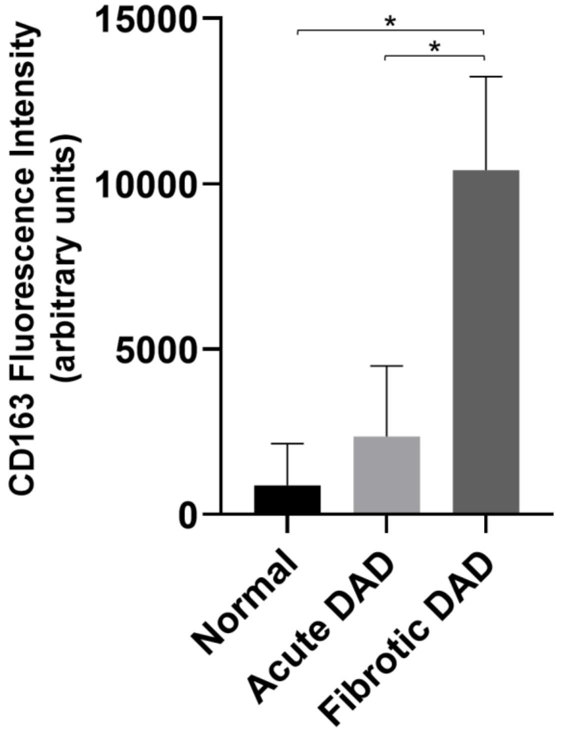

Fig. S19. Semiquantitative analysis of CD163 immunofluorescence in COVID-19 lungs. Macrophage CD163 immunofluorescence image analysis was performed on lung sections from non-COVID-19 patients (n = 3) and COVID-19 cases with predominant acute DAD (n = 5) or fibrotic DAD (n = 4). High resolution images of CD163-stained sections were acquired at 200x magnification and fluorescence intensity measurements were processed by Fiji/ImageJ software (see Supplementary Methods). Values are reported as the means ± SD for each indicated dataset. **P* < 0.05, one-way ANOVA and Tukey post hoc test.

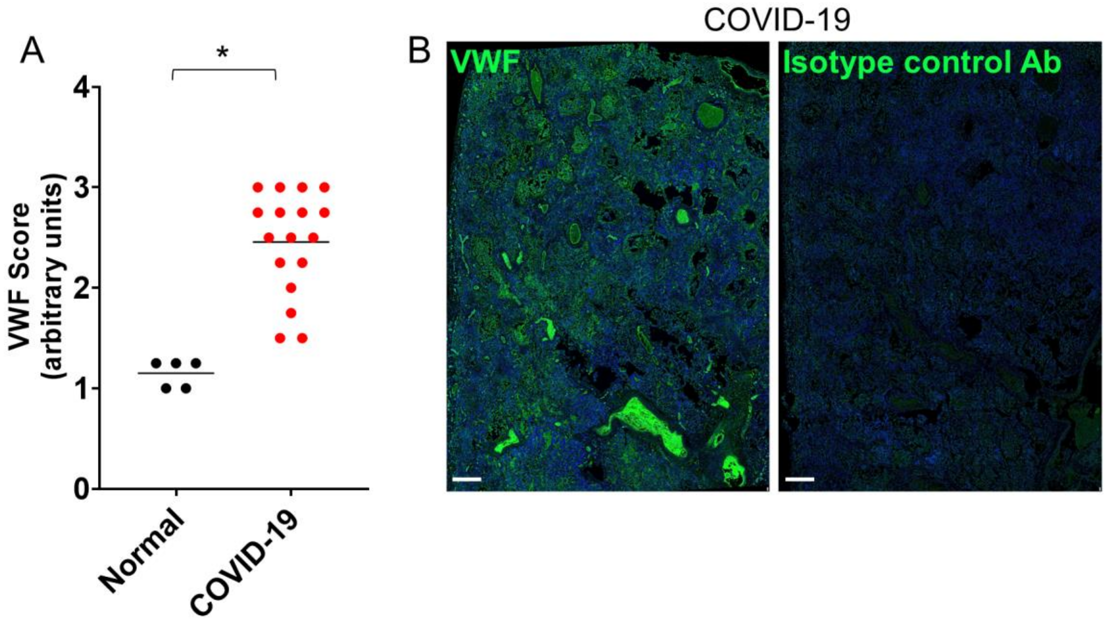

Fig. S20. Semiquantitative analysis of VWF immunofluorescence in COVID-19 lungs. (**A**) VWF immunofluorescence scoring analysis in lung sections from non-COVID-19 patients (n = 5) and COVID-19 cases (n = 17). VWF staining intensity and localization were graded on a scale of 0 - 3 (see Supplementary Methods). Average scores for each case (circles) and the mean of each dataset (horizonal bars) are shown. *$P < 0.05$, Mann-Whitney test. (**B**) Serial lung sections from a COVID-19 lung (case 11) stained with VWF primary antibody or rabbit isotype control antibody. Nuclei were counterstained with Hoechst 33342 (blue). Scale bars: 500 μm.

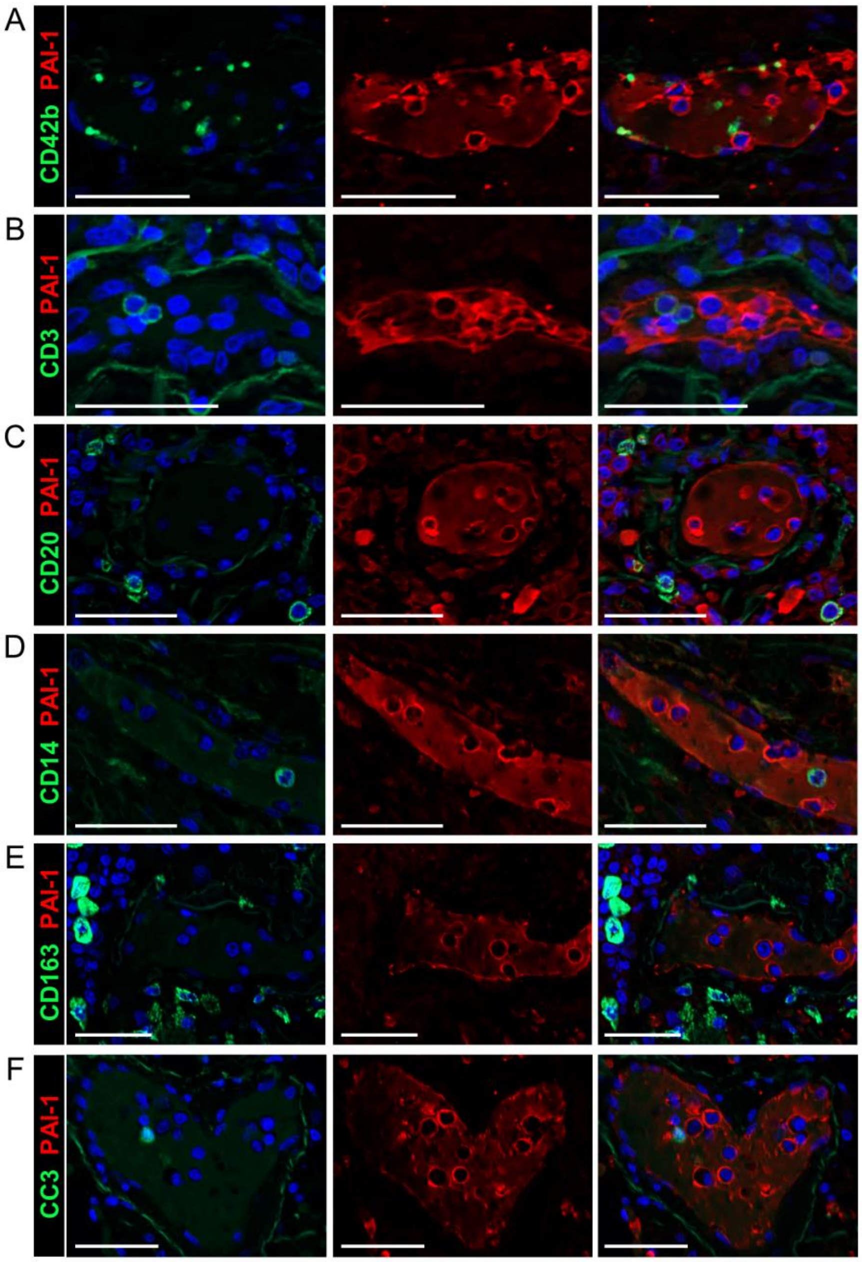

Fig. S21. Clot-entrapped PAI-1 labeled cells do not stain for non-neutrophil immune cells or cleaved caspase 3. Representative immunofluorescence images of pulmonary clots in COVID lungs stained for PAI-1 and common immunomarkers of (**A**) platelets/ megakaryocytes (CD42b), (**B**) T-lymphocytes (CD3), (**C**) B-lymphocytes (CD20), (**D** and **E**) monocytes and macrophages (CD14 and CD163) and (**F**) apoptotic marker cleaved caspase 3 (CC3). Nuclei were counterstained with Hoechst 33342 (blue). Scale bars: 50 µm.

Fig. S22

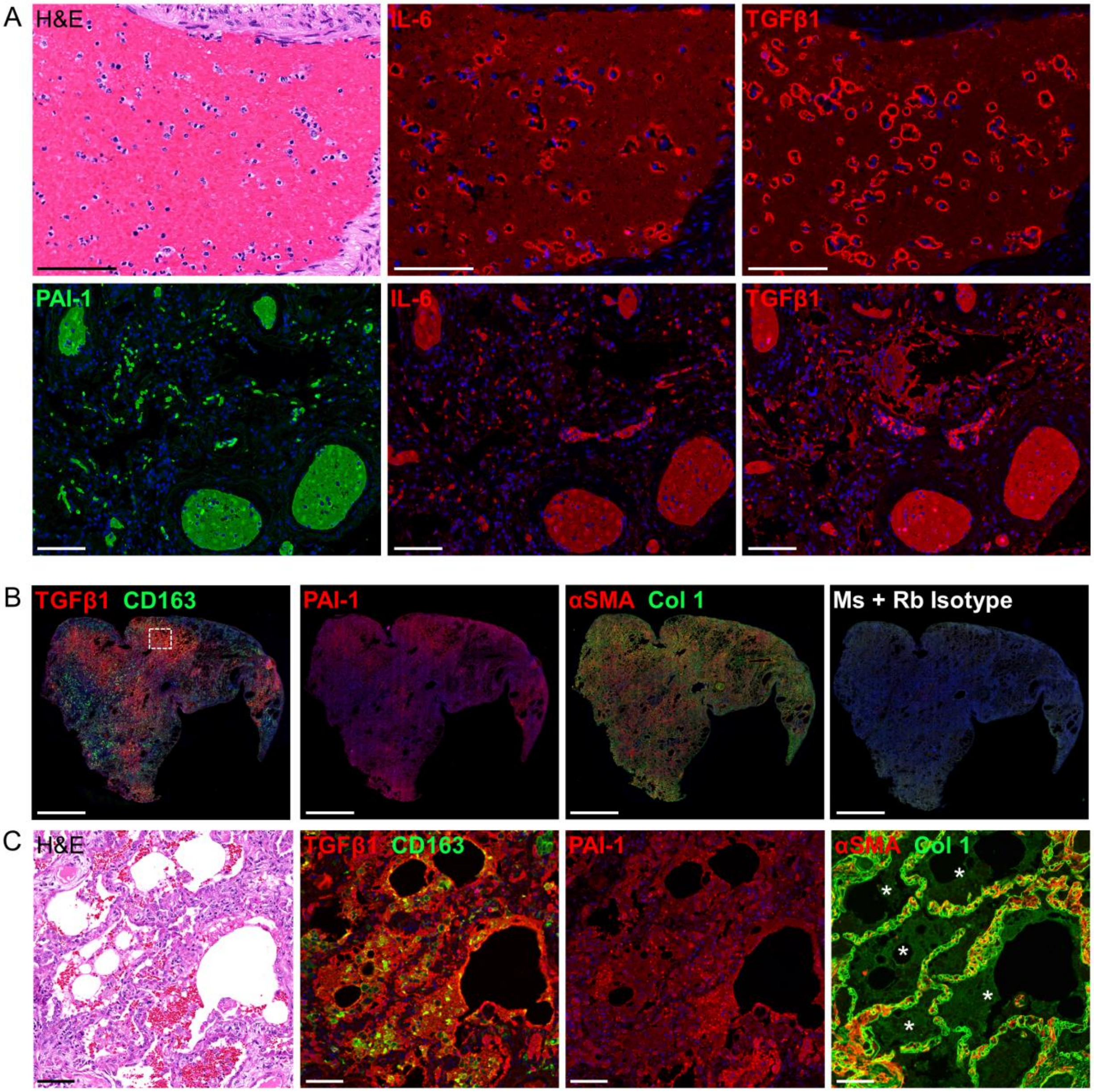

A
H&E
IL-6
TGFβ1
PAI-1
IL-6
TGFβ1
B
TGFβ1 CD163
PAI-1
αSMA Col 1
Ms + Rb Isotype
C
H&E
TGFβ1 CD163
PAI-1
αSMA Col 1

Fig. S22. Increased expression of IL-6 and TGFβ1 in COVID-19 lungs. (**A**) Representative H&E and immunofluorescence staining of IL-6, TGFβ1, and PAI-1 in serial COVID-19 lung sections (case 10). (**B**) Serial lung sections in a COVID-19 patient (case 16) with long duration illness stained for PAI-1, TGFβ1, CD163, αSMA, collagen type 1 (Col 1), and isotype mouse and rabbit antibodies. Low-power images show widespread colocalization of PAI-1 and TGFβ1 in fibrotic lung tissue with excessive CD163-positive macrophages and interstitial αSMA and Col 1 deposition. No staining was observed with mouse and rabbit isotype control antibodies. (**C**) H&E and immunofluorescence images of the digitally magnified boxed area in (B) demonstrate the overlapping staining distribution of PAI-1 and TGFβ1 in alveolar spaces co-expressing CD163-positive macrophages and loose Col 1 deposition but limited αSMA staining (asterisks). Nuclei were counterstained with Hoechst 33342 (blue). Scale bars: 100 μm (A and C); 5000 μm (B).

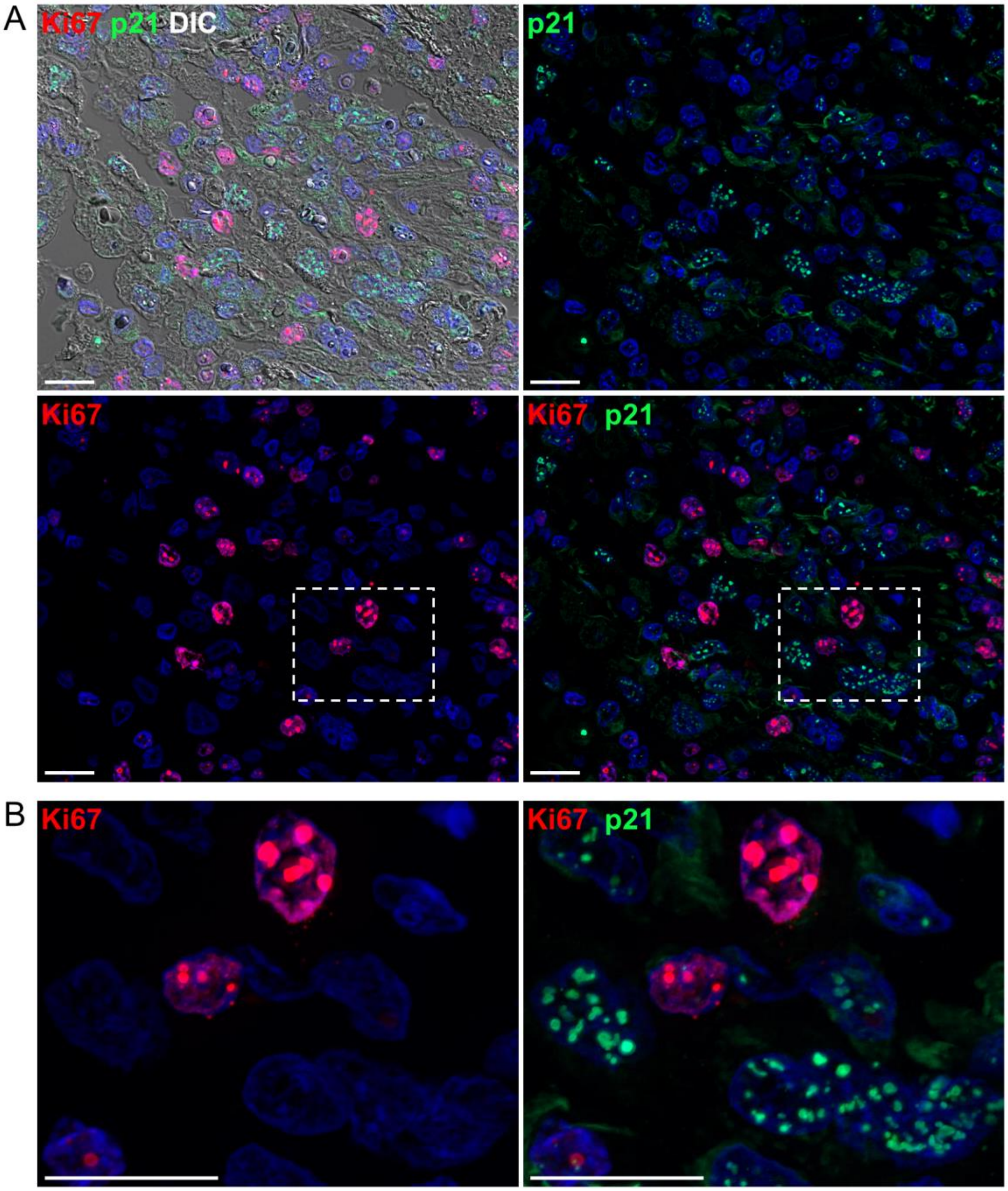

Fig S23. Lack of Ki67 expression in nuclear p21-positive senescent lung cells. (**A**) Representative DIC and immunofluorescence staining of proliferation marker Ki67 and p21 in an epithelial hyperplastic lesion in a COVID-19 lung (case 4). (**B**) High power imaging of the boxed area depicts the absence of Ki67 labeling in p21-expressing senescent cells. Nuclei were counterstained with Hoechst 33342 (blue). Scale bars: 20 µm.

Table S1. Known comorbidities and available laboratory testing

Case No.	Age (y)	Sex	Time of Symptom Onset to Death, SOTD (days)	Known Comorbidities	C-Reactive Protein (mg/dL)*	Ferritin (ng/mL)*	D-dimer (µg/mL FEU)*
1	64	F	3	Obesity, Type II DM, COPD, Hx lung carcinoma	NA	NA	NA
2	60	M	7	Obesity, Type II DM, CAD	NA	NA	NA
3	101	F	7	HTN, Hx cancer, dementia, chronic steroid use	6.8 (4.9)	NA	10.2 (8.9)
4	85	M	7	HTN, CAD	19.4 (2.2)	386 (59)	4.7 (1.4)
5	87	F	7	HTN, CAD, Type II DM, Dementia	9.8	3303	4.3
6	70	M	8	Obesity	4.9 (1.2)	10362 (12093)	9.8 (0.1)
7	50	F	9	Type II DM, HTN	NA	NA	NA
8	73	M	11	HTN, Type II DM, COPD	8.3 (3.0)	1300 (195)	2.3 (0.3)
9	44	M	13	Obesity, Type II DM, HTN, Hx renal carcinoma	NA	NA	NA
10	86	F	14	Hx cancer	7.6 (5.6)	1258 (201)	1.2 (0.5)
11	64	F	16	Obesity, HTN	NA	NA	NA
12	65	M	17	Type II DM, HTN, peripheral vascular disease	NA	NA	NA
13	81	M	19	Obesity, HTN	7.6 (7.4)	2574 (8354)	5.2 (3.8)
14	39	M	20	Obesity	NA	NA	NA
15	55	F	25	Obesity	NA	NA	NA
16	64	F	40	COPD, HTN	3.6 (4.2)	221 (152)	8.2 (3.6)
17	60	M	40	Obesity	15.4 (6.9)	1411 (578)	7.2 (3.7)
18	70	M	47	Asthma	13.3 (7.0)	940 (287)	7.0 (5.0)

* mean value (± SD) of all available in-hospital test samples for each case. DM = Diabetes Mellitus; CAD = Coronary Artery Disease; COPD = Chronic Obstructive Pulmonary Disease; HTN = Hypertension; Hx = History of; FEU = fibrinogen-equivalent-units; NA = not available. Reference values for normal population: CRP (≤ 0.8 mg/dL); Ferritin (11-336 ng/mL); D-dimer (0.2-0.5 µg/mL FEU).

Table S2. Antemortem plasma antibody and neutralization titers

Case No.	Day of Symptom	IgG		IgM		IgA		Spike+ and RBD+			Neutralization Titer
		Spike	RBD	Spike	RBD	Spike	RBD	IgG	IgM	IgA	
4	6	3.9445	3.9445	3.5184	3.168	1.5595	0.8945	+	+	+	157
8	7	1.462	0.33	2.87	0.623	0.2785	0.079	+	+	-	2306
8	11	3.772	3.5555	3.898	2.9985	1.146	0.61	+	+	+	1927
10	3	0.11	0.0815	0.025	0.094	0.069	0.034	-	-	-	<10
13	11	3.947	3.944	3.122	3.69	3.601	3.9045	+	+	+	368
13	15	3.946	3.9475	1.8915	2.8155	2.878	3.634	+	+	+	1926
16	7	3.941	3.938	2.477	2.19	2.8	2.438	+	+	+	2429
17	6	3.929	3.9135	3.944	3.928	3.9415	3.2495	+	+	+	318
17	13	3.8655	3.892	3.6525	3.7165	3.843	3.282	+	+	+	757
17	17	3.929	3.91	3.846	3.9475	3.9035	3.639	+	+	+	929

Table S3. Host genetic variation detected in RNASeq NGS libraries

Case No.	IFITM3	IFITM3-promoter	APOE	DPP9	OAS3	IFNAR2
1	rs12252-T	rs34481144-G	e3/e3	no coverage	rs10735079-**A**	rs2236757-**G**
2	rs12252-T	rs34481144-G	no coverage	no coverage	rs10735079-G/**A**	no coverage
3	rs12252-T	rs34481144-G	e2/**e4**	rs2109069-**A**	no coverage	rs2236757-G
4	rs12252-T	rs34481144-G/**A**	e3/**e4**	rs2109069-**A**	no coverage	no coverage
7	rs12252-T	rs34481144-G	e3/e3	no coverage	rs10735079-**A**	no coverage
8	rs12252-T	rs34481144-G	e2/e3	no coverage	no coverage	no coverage
9	rs12252-T	rs34481144-G	no coverage	no coverage	no coverage	no coverage
10	rs12252-T	rs34481144-G/**A**	e3/e2	rs2109069-**A**	no coverage	rs2236757-**A**/G
11	rs12252-T	rs34481144-G	e3/e3	no coverage	no coverage	no coverage
12	rs12252-T	rs34481144-G	e3/e3	no coverage	no coverage	no coverage
14	rs12252-T	rs34481144-G	e3/**e4**	no coverage	no coverage	rs2236757-G
15	rs12252-T	rs34481144-G	e3/e3	no coverage	no coverage	no coverage
16	rs12252-T	rs34481144-G	e3/**e4**	rs2109069-G	no coverage	no coverage

Bold and highlighted bases/alleles are the ones related to severity or mortality of COVID-19 as reported (*17-22*).

Table S4. Bacterial composition in samples

Case No.	Total reads rooted to bacteria	Top1 bacterial genus	Total reads rooted to top1 bacterial genus	Top2 bacterial genus	Total reads rooted to top2 bacterial genus
1	2951399	*Rhodococcus*	2488244	*Streptomyces*	45191
2	233837	*Streptococcus*	48925	*Pseudomonas*	24260
3-sample 1*	1306975	*Pseudomonas*	359318	*Acinetobacter*	336218
3-sample 2	578389	*Pseudomonas*	102892	*Moraxella*	100608
4-sample 1	881820	*Acinetobacter*	584619	*Moraxella*	21710
4-sample 2	2210747	*Acinetobacter*	1879641	*Pseudomonas*	15487
7-sample 1	248750	*Rhodococcus*	45802	*Pseudomonas*	26732
7-sample 2	2827268	*Rhodococcus*	2296860	*Streptomyces*	69119
8-sample 1	118739	*Moraxella*	56305	*Acinetobacter*	3309
8-sample 2	226634	*Moraxella*	96681	*Acidovorax*	10956
9	350598	*Hydrogenophaga*	40509	*Clostridium*	30988
10-sample 1	12854469	*Rhodococcus*	12283802	*Moraxella*	85637
10-sample 2	12451423	*Rhodococcus*	12110056	*Moraxella*	21092
11	489194	*Pseudomonas*	50534	*Streptomyces*	35672
14-sample 1	86857	*Corynebacterium*	7079	*Cutibacterium*	6250
14-sample 2	81053	*Streptococcus*	11950	*Cutibacterium*	8136
14-sample 3	85318	*Streptococcus*	9317	*Corynebacterium*	7595
15	6483919	*Rhodococcus*	5805119	*Streptomyces*	56326
16- sample 1	550314	*Moraxella*	48485	*Acinetobacter*	41697
16-sample 2	439016	*Streptomyces*	36788	*Clostridium*	18167
Normal lung1	4604887	Rhodococcus	4411434	Streptomyces	14241
Normal lung2	6208091	Rhodococcus	6065685	Streptomyces	9140
Normal lung3	5814789	Rhodococcus	5639832	Streptomyces	11303

*Cases with >1 FFPE lung sample were processed separately

Table S5. Next Generation Sequencing Data

		RNA-seq library		CoV2 enrichment library		
	Case No.	Total reads	Reads mapped to PV08472*	Total reads	Reads mapped to PV08472	PV08472 coverage
Normal lung	N1	160180855	0		Not Done	0
	N2	53166878	0		Not Done	0
	N3	108268615	0		Not Done	0
CoV2 lung samples	1	130031409	145854			1
	2	94185253	34243			1
	3-1#	89063290	68			
	3-2	70142554	3803	124100696	110242118	1
	4-1	96466972	24	53547639	5438374	0.64
	4-2	52440604	2			
	7	141542679	1497	157066420	134322672	1
	8-1	42490916	15	67876959	111414	1
	8-2	99397041	0			
	9	147083879	177	146750080	99460475	1
	10-1	133092882	206			
	10-2	65923966	1614	126846127	109100024	1
	11	144019076	17	72128177	19294905	0.63
	12	190608325	80	73774946	47005076	0.98
	14-1	34689934	0	71566988	216157	0.32
	14-2	40896522	4			
	14-3	51252557	0			
	15	129194226	2	75950337	402495	0.23
	16-1	150166853	0	53994537	16981	1
	16-2	202940873	0			

* SARS-CoV-2/human/USA/NY-PV08472/2020 was reference sequence
#Cases with >1 FFPE lung sample were processed separately

Table S6. Lineage typing of SARS-CoV-2 strains included in the phylogeny
Lineages were determined using Nextclade and Pangolin[1]

Strain identifier	Nextclade lineage	Pangolin lineage	Main isolation countries
hCoV-19/USA/NY-case 1/2020	20C	B.1	USA, UK, France
hCoV-19/USA/NY-case 2/2020	20C	B.1	USA, UK, France
hCoV-19/USA/CA-case 3/2020	20B	B.1.1.167	Denmark, Russia, Poland, Portugal, France, UK
hCoV-19/USA/CA-case 4/2020	NA	B.1	USA, UK, France
hCoV-19/USA/NY-case 7/2020	20C	B.1	USA, UK, France
hCoV-19/USA/CA-case 8/2020	20C	B.1	USA, UK, France
hCoV-19/USA/NY-case 9/2020	20C	B.1	USA, UK, France
hCoV-19/USA/CA-case 10/2020	20B	B.1.1.172	USA
hCoV-19/USA/NY-case 11/2020	NA	B.1	USA, UK, France
hCoV-19/USA/NY-case 12/20203	NA	B.1	USA, UK, France
hCoV-19/USA/VT-case 14/2020	NA	Low coverage	NA
hCoV-19/USA/NY-case 15/2020	NA	Low coverage	NA
hCoV-19/USA/CA-case 16/2020	20C	B.1	USA, UK, France

[1]Rambaut, A. *et al.* A dynamic nomenclature proposal for SARS-CoV-2 lineages to assist genomic epidemiology. *Nat Microbiol* **5**, 1403–1407 (2020).

Table S7. Non-synonymous viral SNPs*

Case No.	Reference	nt_position	ref_base	snp_base	coverage	snp_perc	ORF	ORF_aa_position	ref_codon	ref_aa	snp_codon	snp_aa	CoV_GLUE_status
1	NY_PV08472	1592	T	A	138	14.71%	ORF1ab	461	ATC	I	AAC	N	Reported
1	NY_PV08472	1601	T	A	138	15.79%	ORF1ab	464	GTT	V	GAT	D	Reported
1	NY_PV08472	5674	A	T	111	12.15%	ORF1ab	1822	ACT	T	TCT	S	Not_reported
1	NY_PV08472	5710	G	A	156	10.71%	ORF1ab	1834	GGT	G	AGT	S	Not_reported
1	NY_PV08472	11861	C	T	274	100%	ORF1ab	3884	TCA	S	TTA	L	Reported
1	NY_PV08472	18110	A	G	157	13.64%	ORF1ab	5967	ATA	I	ATG	M	Not_reported
1	NY_PV08472	18943	C	T	158	100%	ORF1ab	6245	GCA	A	GTA	V	Reported
1	NY_PV08472	19266	A	C	119	12.39%	ORF1ab	6353	ACA	T	CCA	P	Not_reported
1	NY_PV08472	20995	T	A	121	13.45%	ORF1ab	6929	ATG	M	AAG	K	Reported
1	NY_PV08472	24391	C	T	134	12.90%	S	962	CTT	L	TTT	F	Not_reported
1	NY_PV08472	24466	G	C	102	10.10%	S	987	GTT	V	CTT	L	Not_reported
2	NY_PV08472	4077	T	G	298	10.03%	ORF1ab	1289	GAT	D	GAG	E	Not_reported
2	NY_PV08472	4127	C	T	893	99.88%	ORF1ab	1306	GCT	A	GTT	V	Reported
2	NY_PV08472	7339	A	C	228	13.06%	ORF1ab	2377	ATT	I	CTT	L	Not_reported
2	NY_PV08472	8882	C	T	119	100%	ORF1ab	2891	GCA	A	GTA	V	Reported
2	NY_PV08472	11375	A	G	639	99.67%	ORF1ab	3722	TAT	Y	TGT	C	Reported
2	NY_PV08472	27268	C	G	134	100%	ORF6	41	TCT	S	TGT	C	Reported
7	NY_PV08472	253	C	A	10608	22.61%	ORF1ab	15	CAA	Q	AAA	K	Not_reported
7	NY_PV08472	580	C	T	28028	21.95%	ORF1ab	124	CGT	R	TGT	C	Not_reported
7	NY_PV08472	836	G	A	163870	64.26%	ORF1ab	209	GGT	G	GAT	D	Reported
7	NY_PV08472	1582	A	T	44639	10.63%	ORF1ab	458	AAA	K	TAA	STOP	Not_reported
7	NY_PV08472	1936	T	A	7126	11.62%	ORF1ab	576	TAT	Y	AAT	N	Not_reported
7	NY_PV08472	2036	C	T	37277	33.21%	ORF1ab	609	ACT	T	ATT	I	Reported
7	NY_PV08472	4952	C	T	292246	22.55%	ORF1ab	1581	ACG	T	ATG	M	Reported
7	NY_PV08472	6517	T	C	19396	12.78%	ORF1ab	2103	TCT	S	CCT	P	Not_reported

7	NY_PV08472	8836	A	T	209154	15.51%	ORF1ab	2876	ACA	T	TCA	S	Not_reported
7	NY_PV08472	10415	C	T	89163	33.57%	ORF1ab	3402	TCA	S	TTA	L	Reported
7	NY_PV08472	11861	C	T	115213	99.98%	ORF1ab	3884	TCA	S	TTA	L	Reported
7	NY_PV08472	15889	C	T	30938	11.61%	ORF1ab	5227	TCA	S	TTA	L	Reported
7	NY_PV08472	16713	C	T	274712	22.41%	ORF1ab	5502	CGA	R	TGA	STOP	Not_reported
7	NY_PV08472	18456	C	T	317439	66.63%	ORF1ab	6083	CCT	P	TCT	S	Not_reported
7	NY_PV08472	18943	C	T	305176	99.94%	ORF1ab	6245	GCA	A	GTA	V	Reported
7	NY_PV08472	18963	C	T	353890	24.93%	ORF1ab	6252	CCA	P	TCA	S	Not_reported
7	NY_PV08472	21159	C	T	105078	15.93%	ORF1ab	6984	CAC	H	TAC	Y	Not_reported
7	NY_PV08472	22763	C	T	66769	14.91%	S	419	GCT	A	GTT	V	Reported
7	NY_PV08472	23222	C	T	119644	99.95%	S	572	ACT	T	ATT	I	Reported
7	NY_PV08472	25114	C	T	70466	14.68%	S	1203	CTT	L	TTT	F	Not_reported
7	NY_PV08472	26682	G	A	554559	22.07%	M	72	AGA	R	AAA	K	Not_reported
7	NY_PV08472	27927	C	T	535835	17.80%	ORF8	30	CCA	P	CTA	L	Reported
7	NY_PV08472	28903	C	T	148499	13.75%	N	229	CAG	Q	TAG	STOP	Not_reported
7	NY_PV08472	29429	C	T	1116041	21.92%	N	404	TCC	S	TTC	F	Reported
9	NY_PV08472	1301	C	T	421016	20.84%	ORF1ab	364	GCT	A	GTT	V	Reported
9	NY_PV08472	1666	G	T	40828	10.85%	ORF1ab	486	GCT	A	TCT	S	Not_reported
9	NY_PV08472	2635	A	T	14171	16.55%	ORF1ab	809	ACA	T	TCA	S	Not_reported
9	NY_PV08472	3971	T	A	27399	29.95%	ORF1ab	1254	TTA	L	TAA	STOP	Reported
9	NY_PV08472	4201	G	A	17510	18.51%	ORF1ab	1331	GGT	G	AGT	S	Not_reported
9	NY_PV08472	5177	G	C	5506	20.53%	ORF1ab	1656	TGG	W	TCG	S	Reported
9	NY_PV08472	7083	T	A	6667	97.44%	ORF1ab	2291	TGT	C	TGA	STOP	Not_reported
9	NY_PV08472	10724	T	A	298095	42.92%	ORF1ab	3505	CTA	L	CAA	Q	Reported
9	NY_PV08472	11911	G	C	3501	27.33%	ORF1ab	3901	GCT	A	CCT	P	Not_reported
9	NY_PV08472	12086	C	T	157015	54.21%	ORF1ab	3959	ACT	T	ATT	I	Reported
9	NY_PV08472	12440	A	C	7382	48.87%	ORF1ab	4077	TAT	Y	TCT	S	Reported
9	NY_PV08472	12441	T	A	7076	46.21%	ORF1ab	4077	TAT	Y	TAA	STOP	Not_reported

9	NY_PV08472	12576	A	C	3251	50.08%	ORF1ab	4122	TTA	L	TTC	F	Not_reported
9	NY_PV08472	14253	T	C	95012	36.21%	ORF1ab	4682	TGG	W	CGG	R	Not_reported
9	NY_PV08472	15216	A	G	379527	73.74%	ORF1ab	5003	AAC	N	GAC	D	Not_reported
9	NY_PV08472	15224	C	A	459	19.13%	ORF1ab	5005	CAC	H	CAA	Q	Not_reported
9	NY_PV08472	16360	T	G	6577	99.79%	ORF1ab	5384	GTG	V	GGG	G	Reported
9	NY_PV08472	17355	C	T	2291664	11.90%	ORF1ab	5716	CGT	R	TGT	C	Not_reported
9	NY_PV08472	17746	C	T	542671	84.79%	ORF1ab	5846	GCC	A	GTC	V	Reported
9	NY_PV08472	18046	C	T	225235	21.08%	ORF1ab	5946	ACA	T	ATA	I	Reported
9	NY_PV08472	18402	C	T	443249	99.92%	ORF1ab	6065	CCA	P	TCA	S	Not_reported
9	NY_PV08472	18471	C	T	51758	40.28%	ORF1ab	6088	CGT	R	TGT	C	Not_reported
9	NY_PV08472	18594	G	T	208714	32.65%	ORF1ab	6129	GAG	E	TAG	STOP	Not_reported
9	NY_PV08472	19764	C	T	289390	10.16%	ORF1ab	6519	CCA	P	TCA	S	Not_reported
9	NY_PV08472	20746	C	T	8748	33.67%	ORF1ab	6846	ACT	T	ATT	I	Reported
9	NY_PV08472	22618	T	C	11093	54.92%	S	371	TCC	S	CCC	P	Not_reported
9	NY_PV08472	23123	T	A	66994	99.84%	S	539	GTC	V	GAC	D	Not_reported
9	NY_PV08472	25237	C	T	204451	89.49%	S	1244	CTC	L	TTC	F	Not_reported
9	NY_PV08472	25987	C	A	132	87.10%	ORF3a	217	ACT	T	AAT	N	Reported
9	NY_PV08472	27592	A	G	60634	97.42%	ORF7a	85	AAA	K	AGA	R	Reported
11	NY_PV08472	568	A	G	3419	100%	ORF1ab	120	AAG	K	GAG	E	Not_reported
11	NY_PV08472	4643	C	G	1727	100%	ORF1ab	1478	TCT	S	TGT	C	Reported
11	NY_PV08472	14253	T	C	10141	57.75%	ORF1ab	4682	TGG	W	CGG	R	Not_reported
11	NY_PV08472	20010	A	G	7957967	40%	ORF1ab	6601	AAA	K	GAA	E	Not_reported
11	NY_PV08472	22384	C	T	2481	26.90%	S	293	CTT	L	TTT	F	Not_reported
11	NY_PV08472	24357	T	A	1414	100%	S	950	GAT	D	GAA	E	Not_reported
11	NY_PV08472	24941	T	C	999	100%	S	1145	TTA	L	TCA	S	Reported
11	NY_PV08472	28393	C	A	211	66.96%	N	59	CAT	H	AAT	N	Not_reported
12	NY_PV08472	1407	G	C	1284	67.81%	ORF1ab	399	AAG	K	AAC	N	Not_reported
12	NY_PV08472	2752	A	T	556	40.64%	ORF1ab	848	AGG	R	TGG	W	Not_reported

12	NY_PV08472	2944	G	A	24648	31.39%	ORF1ab	912	GGT	G	AGT	S	Not_reported
12	NY_PV08472	3386	A	G	16776	57.09%	ORF1ab	1059	AAT	N	AGT	S	Reported
12	NY_PV08472	4843	C	A	183	10.24%	ORF1ab	1545	CAC	H	AAC	N	Not_reported
12	NY_PV08472	5188	C	A	1774	21.72%	ORF1ab	1660	CAA	Q	AAA	K	Not_reported
12	NY_PV08472	7421	G	C	381557	26.08%	ORF1ab	2404	TGT	C	TCT	S	Reported
12	NY_PV08472	8348	G	C	2718	40.86%	ORF1ab	2713	TGG	W	TCG	S	Reported
12	NY_PV08472	10078	C	A	172752	36.65%	ORF1ab	3290	CTT	L	ATT	I	Not_reported
12	NY_PV08472	11092	T	C	12432	59.58%	ORF1ab	3628	TTT	F	CTT	L	Not_reported
12	NY_PV08472	11093	T	A	12426	64.04%	ORF1ab	3628	TTT	F	TAT	Y	Reported
12	NY_PV08472	11323	G	T	52419	39.72%	ORF1ab	3705	GCT	A	TCT	S	Not_reported
12	NY_PV08472	11324	C	T	55538	50.15%	ORF1ab	3705	GCT	A	GTT	V	Reported
12	NY_PV08472	12473	C	T	566	42.28%	ORF1ab	4088	ACA	T	ATA	I	Reported
12	NY_PV08472	12583	C	T	9372	83.42%	ORF1ab	4125	CCT	P	TCT	S	Not_reported
12	NY_PV08472	12703	G	A	108	20.69%	ORF1ab	4165	GAT	D	AAT	N	Not_reported
12	NY_PV08472	14112	C	T	57116	11.99%	ORF1ab	4635	CCT	P	TCT	S	Not_reported
12	NY_PV08472	15157	C	T	16469	99.09%	ORF1ab	4983	ACA	T	ATA	I	Reported
12	NY_PV08472	15298	T	C	15038	95.97%	ORF1ab	5030	CTT	L	CCT	P	Reported
12	NY_PV08472	15304	G	T	13180	95.66%	ORF1ab	5032	CGC	R	CTC	L	Reported
12	NY_PV08472	17364	C	T	193913	16.87%	ORF1ab	5719	CAC	H	TAC	Y	Not_reported
12	NY_PV08472	18402	C	T	76128	99.37%	ORF1ab	6065	CCA	P	TCA	S	Not_reported
12	NY_PV08472	18812	G	A	28299	20.56%	ORF1ab	6201	ATG	M	ATA	I	Not_reported
12	NY_PV08472	18861	A	G	46254	34.01%	ORF1ab	6218	ACT	T	GCT	A	Not_reported
12	NY_PV08472	18943	C	T	50206	20.06%	ORF1ab	6245	GCA	A	GTA	V	Reported
12	NY_PV08472	20613	G	T	4821	29.28%	ORF1ab	6802	GCG	A	TCG	S	Not_reported
12	NY_PV08472	20764	T	C	4221	90.21%	ORF1ab	6852	TTA	L	TCA	S	Reported
12	NY_PV08472	25974	C	A	61708	26.88%	ORF3a	213	CAG	Q	AAG	K	Not_reported
12	NY_PV08472	26139	A	T	10773	17.62%	ORF3a	268	ACG	T	TCG	S	Not_reported
12	NY_PV08472	26143	C	T	11273	16.20%	ORF3a	269	ACG	T	ATG	M	Reported

15	NY_PV08472	22618	T	C	10797	99.98%	S	371	TCC	S	CCC	P	Not_reported
10	NY_PV08472	721	C	T	45101	20.13%	ORF1ab	171	CGT	R	TGT	C	Not_reported
10	NY_PV08472	970	C	T	174667	21.35%	ORF1ab	254	CCT	P	TCT	S	Not_reported
10	NY_PV08472	1004	T	C	176959	99.94%	ORF1ab	265	ATC	I	ACC	T	Reported
10	NY_PV08472	4649	C	T	204226	11.34%	ORF1ab	1480	CCT	P	CTT	L	Reported
10	NY_PV08472	6484	C	T	84063	10.50%	ORF1ab	2092	CAC	H	TAC	Y	Not_reported
10	NY_PV08472	6685	C	T	82363	11.64%	ORF1ab	2159	CGG	R	TGG	W	Not_reported
10	NY_PV08472	6927	C	A	226025	10.03%	ORF1ab	2239	TGC	C	TGA	STOP	Not_reported
10	NY_PV08472	8414	A	G	128981	15.79%	ORF1ab	2735	AAG	K	AGG	R	Reported
10	NY_PV08472	8747	C	T	608481	29.59%	ORF1ab	2846	ACT	T	ATT	I	Reported
10	NY_PV08472	9059	C	T	47346	11.54%	ORF1ab	2950	CCT	P	CTT	L	Reported
10	NY_PV08472	9155	A	G	73617	31.11%	ORF1ab	2982	GAG	E	GGG	G	Reported
10	NY_PV08472	10704	G	A	509476	10.95%	ORF1ab	3498	ATG	M	ATA	I	Not_reported
10	NY_PV08472	19287	G	T	87207	14.91%	ORF1ab	6360	GCT	A	TCT	S	Not_reported
10	NY_PV08472	19320	T	C	104684	15.79%	ORF1ab	6371	TAT	Y	CAT	H	Not_reported
10	NY_PV08472	20764	T	C	181115	10.79%	ORF1ab	6852	TTA	L	TCA	S	Reported
10	NY_PV08472	21354	C	T	416302	21.44%	ORF1ab	7049	CCC	P	TCC	S	Not_reported
10	NY_PV08472	23933	C	T	361977	13.34%	S	809	CCA	P	CTA	L	Reported
10	NY_PV08472	24158	C	T	84836	11.68%	S	884	TCT	S	TTT	F	Reported
10	NY_PV08472	24580	G	T	217567	10.78%	S	1025	GCT	A	TCT	S	Not_reported
10	NY_PV08472	25508	T	G	256610	99.82%	ORF3a	57	CAT	H	CAG	Q	Not_reported
10	NY_PV08472	27977	A	T	286397	99.95%	ORF8	47	ATT	I	TTT	F	Not_reported
10	NY_PV08472	28826	G	A	95068	99.96%	N	203	AGG	R	AAG	K	Reported
10	NY_PV08472	28828	G	C	95044	99.93%	N	204	GGA	G	CGA	R	Not_reported
3	NY_PV08472	1004	T	C	95007	99.98%	ORF1ab	265	ATC	I	ACC	T	Reported
3	NY_PV08472	3598	C	T	116576	58.32%	ORF1ab	1130	CTT	L	TTT	F	Not_reported
3	NY_PV08472	9109	A	G	40404	11.51%	ORF1ab	2967	ACC	T	GCC	A	Not_reported
3	NY_PV08472	9191	C	T	60273	34.38%	ORF1ab	2994	GCT	A	GTT	V	Reported

3	NY_PV08472	13629	C	T	127062	12.19%	ORF1ab	4474	CAT	H	TAT	Y	Not_reported
3	NY_PV08472	24782	A	C	249984	98.96%	S	1092	GAA	E	GCA	A	Reported
3	NY_PV08472	25508	T	G	132536	99.84%	ORF3a	57	CAT	H	CAG	Q	Not_reported
3	NY_PV08472	26044	T	C	42433	99.96%	ORF3a	236	ATT	I	ACT	T	Reported
3	NY_PV08472	28826	G	A	81877	99.92%	N	203	AGG	R	AAG	K	Reported
3	NY_PV08472	28828	G	C	81870	99.95%	N	204	GGA	G	CGA	R	Not_reported
4	NY_PV08472	1004	T	C	1690	100%	ORF1ab	265	ATC	I	ACC	T	Reported
4	NY_PV08472	14253	T	A	2123	78.20%	ORF1ab	4682	TGG	W	AGG	R	Not_reported
4	NY_PV08472	22094	A	G	21577	33.47%	S	196	AAT	N	AGT	S	Reported
4	NY_PV08472	22321	C	T	7059	97.27%	S	272	CCT	P	TCT	S	Not_reported
4	NY_PV08472	23389	C	T	124581	97.78%	S	628	CAA	Q	TAA	STOP	Not_reported
4	NY_PV08472	23737	G	T	10761	10.49%	S	744	GGT	G	TGT	C	Not_reported

*SNP call criteria: site coverage > 100 reads; Q value >25; variant base > 10%; variant base pass strand filter; SNP call p-value < 0.01

Table S8. Summary of detected SNPs in all the samples

Sample	ORF1ab	S	ORF3a	E	M	ORF6	ORF7a	ORF8	N	NotInCDS	Syn	Total
1	9	2								1	2	14
2	5					1					1	7
3	5	1	2						2		7	17
4	2	4									6	12
7	17	3			1			1	2	1	11	36
8												0
9	25	3	1				1				15	45
10	16	3	1					1	2		10	33
11	4	3							1	1	2	11
12	27		3								15	45
14											2	2
15		1										1
16												0

Table S9. Shared SNPs/SNP positions among samples

Ref. nucleotide position	Ref. nt	SNP_ nt	ORF	ORF AA position	Ref. codon	Ref. AA	SNP Codon	SNP AA	Samples
1004	T	C	ORF1ab	265	ATC	I	ACC	T	3, 4, 10
11861	C	T	ORF1ab	3884	TCA	S	TTA	L	1, 7
14252	T	C	ORF1ab	4681	TAT	Y	TAC	Y	3, 7, 9, 12
14253	T	C,A	ORF1ab	4682	TGG	W	CGG,,AGG	R	4, 9, 11
14261	G	A	ORF1ab	4684	CAG	Q	CAA	Q	9, 12
16814	C	T	ORF1ab	5535	TAC	Y	TAT	Y	9, 12
18402	C	T	ORF1ab	6065	CCA	P	TCA	S	9, 12
18943	C	T	ORF1ab	6245	GCA	A	GTA	V	2, 7, 12
20764	T	C	ORF1ab	6852	TTA	L	TCA	S	10, 12
22618	T	C	S	371	TCC	S	CCC	P	9, 15
24465	G	A	S	986	AAG	K	AAA	K	1, 3, 7, 9, 10, 12
25508	T	G	ORF3a	57	CAT	H	CAG	Q	3, 10
28826	G	A	N	203	AGG	R	AAA	K	3, 10

28827	G	A	N	203	AGG	R	AAA	K	3, 10
28828	G	C	N	204	GGA	G	CGA	R	3, 10
29485	G	A	NotInCDS						1, 7

Table S10. D614G mutation survey

Sample	23348 position coverage	Number of As
1	876	0
2	169	0
3-1		
3-2	535014	40
4-1	70187	1
4-2		
8-1	77	0
8-2		
9	191674	12
10-1		
10-2	464520	37
11	3199	0
12-1	422978	28
12-2	16435	1
14-1	1	0
15	0	
16-1	59	0
16-2		

S protein amino acid 614 position in Wuhan strain (MN975262_01/2020_China) is at 23403 and is A (GAT D) and it is G (GGT G in frame 1) at 23348 in NY strain (NY_PV08472, reference in this study).

Table S11. Histopathological grading of DAD stages in COVID-19 cases

Case No.	Time of Symptom Onset to Death, SOTD (days)	Acute DAD*	Organizing DAD*	Fibrotic DAD*
1	3	3.0 (0.0)	1.1 (0.4)	0.0 (0.0)
2	7	2.9 (0.4)	1.6 (0.5)	0.0 (0.0)
3	7	1.1 (0.4)	2.3 (0.7)	2.6 (0.5)
4	7	1.9 (0.4)	2.9 (0.4)	1.9 (0.6)
5	7	1.1 (0.4)	2.9 (0.4)	1.6 (0.5)
6	8	2.6 (0.5)	2.6 (0.5)	0.0 (0.0)
7	9	2.9 (0.4)	2.6 (0.5)	0.0 (0.0)
8	11	0.1 (0.4)	2.3 (0.9)	0.5 (0.8)
9†	13	3.0 (0.0)	0.0 (0.0)	0.0 (0.0)
10	14	1.5 (0.5)	2.8 (0.5)	1.8 (0.5)
11	16	3.0 (0.0)	1.6 (0.5)	0.1 (0.4)
12	17	2.6 (0.5)	2.3 (0.7)	0.5 (0.5)
13	19	0.0 (0.0)	1.4 (0.5)	3.0 (0.0)
14	20	0.4 (0.5)	3.0 (0.0)	0.3 (0.5)
15	25	2.9 (0.4)	0.4 (0.5)	0.0 (0.0)
16	40	0.9 (0.4)	3.0 (0.0)	2.6 (0.5)
17	40	0.0 (0.0)	1.8 (0.5)	3.0 (0.0)
18	47	0.0 (0.0)	1.0 (0.0)	3.0 (0.0)

* Average histopathological score (± SD) for the specified DAD stage evaluated for each COVID-19 case (see Supplementary Methods).

† Widespread tissue necrosis and edema with large a pulmonary clot observed in this patient lung.

Table S12. List of primary antibodies for immunohistochemistry and immunofluorescence analyses.

Primary Antibodies	Company	Species	Type	Reference#
Alpha-SMA	Abcam	Mouse	Monoclonal	ab7817
CD14	Abcam	Rabbit	Monoclonal	ab183322
CD15	Biocare Medical	Mouse	Monoclonal	CM073
CD163	Abcam	Rabbit	Monoclonal	ab182422
CD20	Abcam	Rabbit	Monoclonal	ab78237
CD3	Abcam	Rabbit	Monoclonal	ab16669
CD31	Santa Cruz	Rabbit	Polyclonal	sc1506-R
CD42b	Abcam	Rabbit	Monoclonal	ab183345
CD61	Vector Labs	Mouse	Monoclonal	VP-C362
Claudin-5	ThermoFisher	Mouse	Monoclonal	35-2500
Claudin-5	Abcam	Rabbit	Monoclonal	ab131259
Cleaved caspase 3	Cell Signaling	Rabbit	Polyclonal	9661
Collagen Type I	Abcam	Rabbit	Polyclonal	ab34710
Collagen Type IV	Abcam	Rabbit	Polyclonal	ab6586
pan-Cytokeratin	Abcam	Mouse	Monoclonal	ab27988
E-cadherin	Abcam	Mouse	Monoclonal	ab219332
ERG	Biocare Medical	Mouse	Monoclonal	CM421
Fibrin	EMD Millipore	Mouse	Monoclonal	MABS2155
Histone H3, citrullinated	Abcam	Rabbit	Polyclonal	ab5103
IL-6	Abcam	Mouse	Monoclonal	ab9324
Ki67	Abcam	Rabbit	Monoclonal	ab16667
Ki67	Cell Signaling	Mouse	Monoclonal	9449
MPO	Abcam	Rabbit	Polyclonal	ab9535
MPO	Novus Biologicals	Mouse	Monoclonal	MAB3174
Neutrophil elastase	Abcam	Rabbit	Polyclonal	ab68672
p21	Proteintech	Rabbit	Polyclonal	10355-1-AP
PAI-1	ThermoFisher	Mouse	Monoclonal	MA5-17171
Pro-SPC	EMD Millipore	Rabbit	Polyclonal	AB3786
SARS-CoV-2 NP	Sino Biological	Mouse	Monoclonal	40143-MM05
TGFβ1	ThermoFisher	Mouse	Monoclonal	MA5-16949
Tissue Factor	ThermoFisher	Mouse	Monoclonal	MA1-83495
VWF	Abcam	Rabbit	Polyclonal	ab9378
γH2AX	ThermoFisher	Rabbit	Polyclonal	A300-081A
Mouse IgG isotype controls	ThermoFisher	-	-	02-6502, 14-4714-82
Rabbit IgG isotype control	ThermoFisher	-	-	02-6102
